# Thioctic Acid Derivatives as Building Blocks to Incorporate DNA Oligonucleotides onto Gold Nanoparticles

**DOI:** 10.3390/molecules190710495

**Published:** 2014-07-18

**Authors:** Sónia Pérez-Rentero, Santiago Grijalvo, Guillem Peñuelas, Carme Fàbrega, Ramon Eritja

**Affiliations:** 1 Institute for Advanced Chemistry of Catalonia (IQAC), Consejo Superior de Investigaciones Científicas (CSIC), Jordi Girona 18-26, E-08034 Barcelona, Spain; E-Mails: sonia.perez@iqac.csic.es (S.P.-R.); santiago.grijalvo@iqac.csic.es (S.G.); guillemph90@gmail.com (G.P.); carme.fabrega@iqac.csic.es (C.F.); 2 Networking Center on Bioengineering, Biomaterials and Nanomedicine (CIBER-BBN), Barcelona, 08034, Spain

**Keywords:** thioctic acid, threoninol, DNA, DNA-gold nanoparticle conjugates, stability, surface coverage, cathepsin

## Abstract

Oligonucleotide gold nanoparticle conjugates are being used as diagnostic tools and gene silencing experiments. Thiol-chemistry is mostly used to functionalize gold nanoparticles with oligonucleotides and to incorporate DNA or RNA molecules onto gold surfaces. However, the stability of such nucleic acid–gold nanoparticle conjugates in certain conditions may be a limitation due to premature break of the thiol-gold bonds followed by aggregation processes. Here, we describe a straightforward synthesis of oligonucleotides carrying thioctic acid moiety based on the use of several thioctic acid-l-threoninol derivatives containing different spacers, including triglycine, short polyethyleneglycol, or aliphatic spacers. The novel thioctic-oligonucleotides were used for the functionalization of gold nanoparticles and the surface coverage and stability of the resulting thioctic-oligonucleotide gold nanoparticles were assessed. In all cases gold nanoparticles functionalized with thioctic-oligonucleotides had higher loadings and higher stability in the presence of thiols than gold nanoparticles prepared with commercially available thiol-oligonucleotides. Furthermore, the thioctic derivative carrying the triglycine linker is sensitive to cathepsin B present in endosomes. In this way this derivative may be interesting for the cellular delivery of therapeutic oligonucleotides as these results provides the basis for a potential endosomal escape.

## 1. Introduction

During the past decade colloidal gold nanoparticles (AuNPs) have become attractive materials due to an increasing interest in their potential applications in biotechnology, molecular diagnostics and biomedicine [1,2,3,4,5,6]. Because of their unique properties such as size and shape-dependent optical and electronic features, good biocompatibility and the ability to bind ligands containing thiols, phosphines and amines, they have emerged as useful tools for the design of biosensors [3], the development of methods for cancer detection [7,8] and potential vehicles for drug delivery [9]. The use of AuNPs requires an appropriate colloidal stability. The immobilization of organic molecules or biomolecules onto AuNps surface significantly promotes their dispersion in aqueous solutions and improves the stability of the colloidal suspensions [10].

There are numerous examples of biomolecules that have been immobilized onto AuNPs such as peptides [11], proteins [12,13] or nucleic acids [14]. Specifically, there are many examples reported in the literature on the use of nucleic acid-functionalized gold nanoparticles. For instance, they have been applied as a scaffold to construct higher ordered materials [15] or in the field of biodiagnostics [6,15] and bioimaging [16] and as well used for therapeutic applications such as a potent cell transfection and gene regulation material [6].

The conjugation of AuNps with biomolecules such as oligonucleotides, proteins and antibodies can be carried out through chemisorption of the biomolecule to the particle surface or electrostatic interactions between the biomolecule. The use of heterobifunctional ligands allows the functionalization of AuNps with biomolecules through covalent attachments, or specific biomacromolecular interactions such as streptavidin/biotin [17,18,19,20].

Due to the strong affinity of thiolated species for gold surfaces and the ease of modification of nucleic acids with thiolated derivatives, thiolated oligonucleotides have been extensively used in the preparation of DNA-functionalized AuNps since the pioneering publication of Mirkin and co-workers in 1996 [21].

Although such oligonucleotide-AuNP conjugates have been demonstrated to be very stable under most DNA detection conditions, limited stability has been observed in certain biological media due to a partial desorption of thiolated oligonucleotides by ligand exchange in the presence of some additives (e.g., dithiothreitol (DTT), mercaptoethanol or biogenic thiols) [22,23,24].

Many efforts have been carried out in order to increase the colloidal stability by designing surface ligands that keep AuNPs from aggregating. For instance, Taton *et al.* functionalized AuNPs with thiolated systems like poly(L-lysine)-graft-poly(ethyleneglycol (PLL-g-PEG graft copolymers). The conjugates were stable under extreme conditions, making this polymer a promising ligand to use in biotechnological applications [24]. There are also several examples reported in the literature to enhance the stability in systems containing oligonucleotide-AuNP conjugates. Mirkin *et al.* described a few years ago the use of modified steroid cyclic disulfide or a novel trithiol-capped oligonucleotide that exhibited higher stabilities than the conventional acyclic monothiol-capped oligonucleotide-AuNp conjugates toward DTT treatment. These stable conjugates were studied as useful tools for diagnostic probes in colorimetric assays and detection methods [21].

Thioctic acid (TA) has been extensively used for the functionalization of gold surfaces [18]. In most cases, TA has been conjugated with polyethyleneglycol (PEG) units giving decorated TA-appended-polyethyleneglycol functionalized AuNPs, thereby showing better stabilities than their acyclic disulfide and single thiol counterparts [18,25,26,27]. The conjugation of oligonucleotides with TA has been recently reported by Graham and co-workers using two different methodologies [28,29]. These include: (I) the use of a TA H-phosphonate derivative to introduce the resultant disulfide unit at 5'-termini of oligonucleotides [28] and (II) the use of the *N*-hydroxy-succimidyl ester of TA to incorporate the TA molecule in a solid support having an amino-linker followed by the assembly of the oligonucleotide by the stepwise approach [29]. 

Another important factor to consider AuNPs as promising vehicles for drug delivery, apart from their systemic stability, is the selective cargo release at the target tissue [30,31]. In these terms, the conjugation of oligonucleotides with TA derivatives could facilitate the incorporation of spacers with potential breaking points that would allow oligonucleotides to be released at the cellular target site. It is known that several classes of proteinases are overexpressed by many tumor cells, including cathepsins. In particular, cathepsin B plays a critical role in tumor progression [32]. In addition, several works has reported that polypeptides linker has been used for the endosomal escape of several drugs [33,34,35,36] because of the lability to cathepsin. Thus, we thought the possibility to consider the incorporation of linkers susceptible to cathepsin B in our oligonucleotide-TA conjugates in order to release the therapeutic oligonucleotide by the action of this protease.

**Figure 1:**
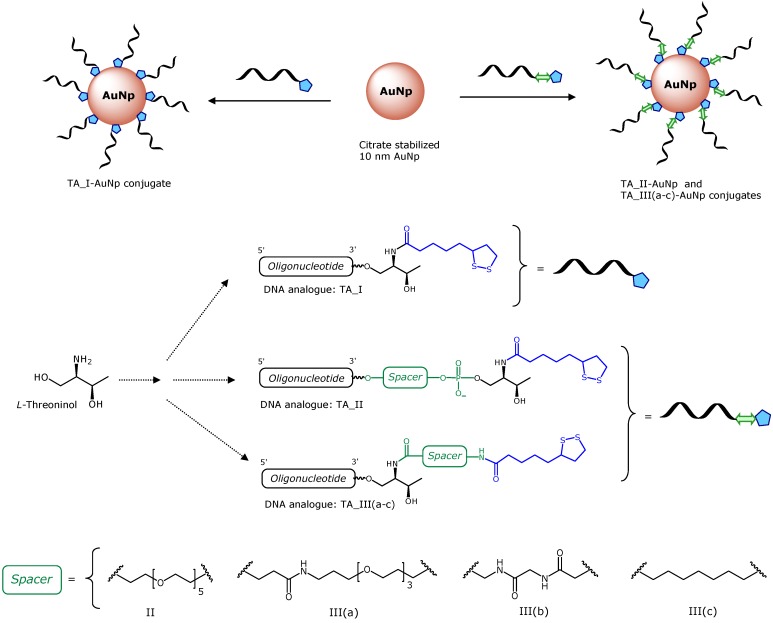
Preparation of gold nanoparticles functionalized with TA-terminated oligonucleotides.

In this work, we investigate the effect of introducing several spacers between the oligonucleotide and the TA moiety (Figure 1). It has been reported that the introduction of spacers between the AuNPs surface and biomolecules not only leads to an increased immobilization efficiency [37,38], but also to functional immobilized biomolecules [38,39]. Moreover, spacers have been employed to protect the gold surface from thiolated compounds by providing a dense and ordered coating layer [27,40,41,42]. Then, the contribution of a spacer, in addition of moving the recognition tracts of oligonucleotides from the particle surface, may be crucial to the stability of the resulting AuNP conjugates.

Here, L-threoninol was selected as a building block to incorporate the TA residue tethered with different spacers into DNA (Figure 1). We synthesized the thioctic-DNA analogue (**TA_I**) without any spacer, which was used as a reference and four different thioctic-DNA analogues containing a spacer. In particular, **TA_II** conjugate containing an ethylene glycol-like (hexaethylene glycol) unit between the oligonucleotide and the threoninol moiety was obtained via phosphate linkages. Finally, the **TA_III** series containing an oligo(ethylene glycol)-like molecule, a triglycine peptide and an alkyl group as spacers. These three spacers were introduced via amide groups in order to separate the threoninol unit from the TA residue. The resulting oligonucleotides were then successfully loaded onto AuNPs. The ability of these TA-terminated oligonucleotides to improve the stability of the corresponding DNA-AuNP conjugates in aqueous solution in the presence of DTT was investigated. For comparison purposes, the same experiments were performed with AuNP conjugates prepared with oligonucleotides modified with commercially available thiol- and acyclic disulfide-oligonucleotides. Consequently, this synthetic strategy may be useful to prepare TA-terminated oligonucleotides modified at the 3'-end by the incorporation of several spacers with different size and properties. To the best of our knowledge, the use of this strategy in synthesizing such modified oligonucleotides to functionalize AuNPs has not been reported yet. In addition, the effect promoted by some proteases like Cathepsin B on our TA-terminated oligonucleotides was evaluated. These preliminary results could offer a strategy in the search of new drug delivery vehicles containing oligonucleotides.

## 2. Results and Discussion

### 2.1. Synthesis of Solid Supports Functionalized with TA Derivatives

In order to connect TA to the oligonucleotides we used the readily available aminodiol L-threoninol (Figure 1). The amino group of L-threoninol allows the selective addition of the TA leaving the primary and secondary alcohol functions for the preparation of DMT building blocks needed for oligonucleotide synthesis. L-Threoninol has been extensively used to introduce all kinds of interesting molecules containing a carboxylic acid function into oligonucleotides [43] including a thiol group protected with the *S*-*tert*-butylsulfanyl group [44]. It was envisaged that the threoninol unit would allow us to directly connect the TA molecule with the oligonucleotide chain (**TA_I**, Figure 1) or after a commercially available hexaethylene glycol spacer (**TA_II**, Figure 1). In addition, the threoninol linker will allow the introduction of several spacers between the threoninol molecule and the oligonucleotide. We selected the following molecules as spacers: (i) an oligo(ethylene glycol) molecule (**TA_III(a)**, Figure 1); (ii) a short peptide (triglycine, **TA_III(b)**, Figure 1) and (iii) an aliphatic chain (**TA_III(c)**, Figure 1).

**Scheme 1:**
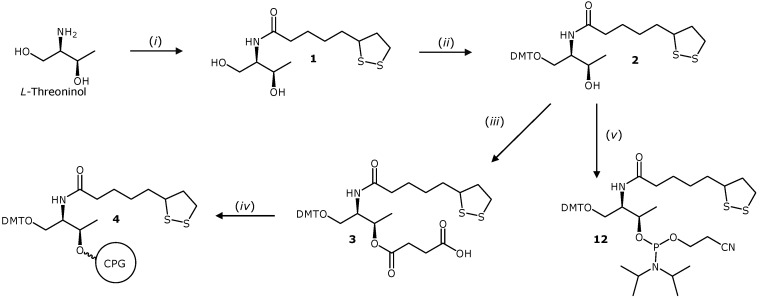
Synthesis of solid support **4** and phosphoramidite **12** used for the preparation of 3'-and 5'-TA oligonucleotides **TA_I**, **TA_II** and **5'TA_I**, respectively.

The preparation of the appropriate solid supports carrying the TA derivatives was undertaken in order to obtain 3'-TA oligonucleotides (**TA_I**, **TA_II** and **TA_III** respectively). First, the TA threoninol derivative (compound **1**, Scheme 1) was prepared. L-threoninol was reacted with TA via amide bond formation by activating the carboxylic acid with HOBt and EDCI. Next, the primary alcohol was protected with the 4,4'-dimethoxytrityl (DMT) group to give the compound **2** and subsequently, reacting the secondary alcohol with succinic anhydride yielded the derivative **3**. This compound was used to functionalize the amino-controlled pore glass support (long chain amino alkyl-controlled pore glass, LCAA-CPG) to yield the CPG solid support **4**. This solid support was used to prepare oligonucleotides labeled as **TA_I** (see below) and **TA_II** with the hexaethylene glycol spacer using the commercially available phosphoramidite (Figure 1).

*Reagents and conditions*: (i) Thioctic acid, EDCI, HOBt, DIEA, DMF; (ii) dimethoxytrityl chloride, pyr; (iii) succinic anhydride, DMAP, pyr; LCAA CPG (long chain aminoalkyl controlled pore glass), TBTU, TEA, ACN; (v) 2-cyanoethyl-*N*,*N*-diisopropylaminohlorophosphine, DIEA, DCM.

**Scheme 2:**
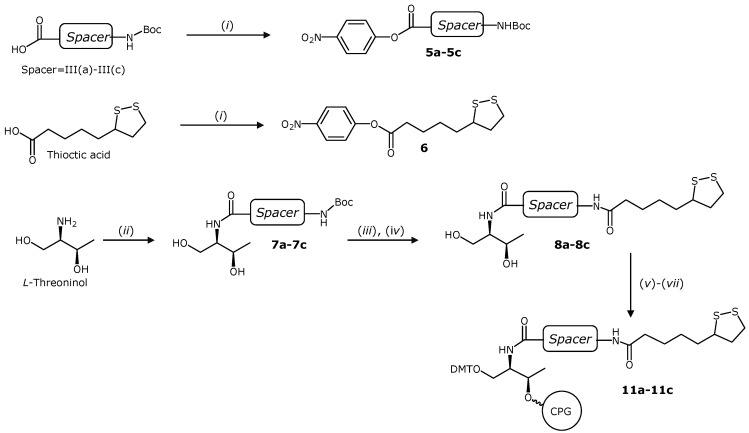
Synthesis of the solid supports needed for the preparation of 3'-TA- terminated oligonucleotides **TA_III(a)**, **TA_III(b)** and **TA_III(c)**.

Next, three different TA-terminated oligonucleotides (**TA_III(a)**, **TA_III(b)** and **TA_III(c)**), containing different spacers such as an oligo(ethylene glycol)-like molecule, a triglycine peptide and an alkyl group respectively were prepared. The synthesis of the solid supports **11a**-**11c** required for the preparation of the corresponding DNA analogues is depicted in Scheme 2. The corresponding carboxylic acids *N*-Boc-*N*'-succinyl-4,7,10-trioxa-1,13-tridecanediamine, Boc-Gly-Gly-Gly-OH and *N*-Boc-8-aminooctanoic acid were first transformed into their 4-nitrophenyl esters (**5a**–**5c**) and subsequently reacted with L-threoninol to obtain the corresponding *N*-Boc-compounds **7a**, **7b** and **7c** in good yield (69%, 78% and 96%, respectively). Then, the Boc group was removed under acidic conditions yielding the corresponding amines as trifluoroacetate salts, which were used in the next step without further purification. Next, the reaction of the resulting free amines analogues with TA, using the same “active-ester” methodology described before but this time using the 4-nitrophenyl ester **6** yielded compounds **8a**, **8b** and **8c**. Finally, the primary alcohol was protected with a DMT group and the corresponding 3'-hemisuccinate derivatives were synthesized to prepare the corresponding functionalized solid supports **11a**–**11c** as described above.

*Reagents and conditions*: (i) Carboxylic acids III(a)-III(c) or thioctic acid, DCC, *p*-nitrophenol, pyr.; (ii) **5a**–**5c**, DMF; (iii) TFA 10% DCM; (iv) **6**, DMF. (v) dimethoxytrityl chloride, pyr.; (vi) succinic anhydride, DMAP, pyr.; (vii) LCAA CPG (long chain aminoalkyl controlled pore glass), TBTU, TEA, ACN.

Furthermore, as a proof of concept about the versatility of the present study, we also derivatized the DMT-protected alcohol **2** to synthesize the corresponding phosphoramidite **12** (Scheme 1), which was obtained in good yield (77%) after purification by flash chromatography.

### 2.2. Synthesis of Novel TA Terminated Oligonucleotides and Alkylthiolated Modified Oligonucleotides

**Figure 2:**
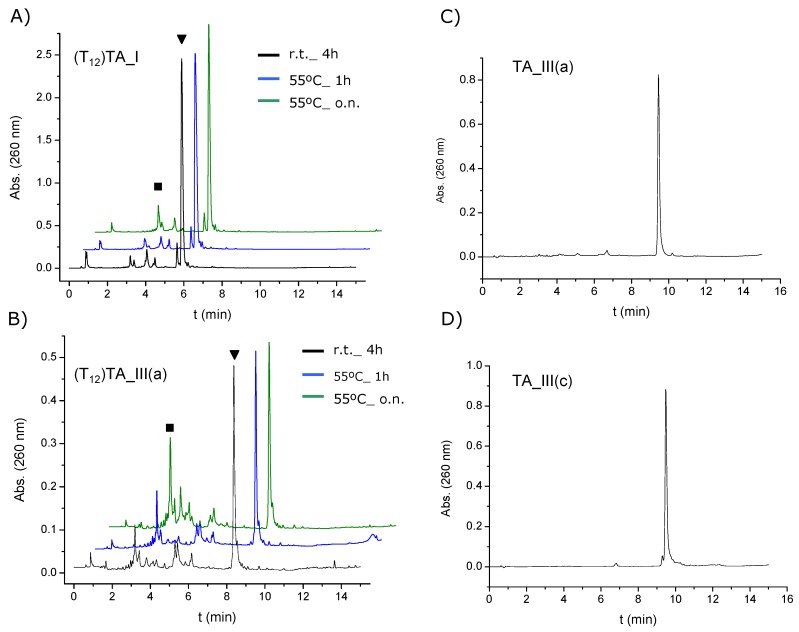
HPLC profiles of model oligonucleotides (XBridge OST C_18_ column). (**A**) **(T_12_)TA_I** and (**B**) **(T_12_)TA_III(a)** after different treatments with aqueous concentrated ammonia. Example of HPLC profiles of purified oligonucleotides; (**C**) **TA_III(a)** and (**D**) **TA_III(c)**. ▼ stands for TA-terminated oligonucleotides and ■ stands for a β-elimination side product.

Prior to the synthesis of modified oligonucleotides to functionalize AuNps, we prepared the dodecathymidine oligonucleotide sequence in order to study their stability under synthesis and cleavage conditions. Then we used the solid supports **4** and **11a**–**11c** to synthesize oligonucleotides **(T_12_)TA_I**, and **(T_12_)TA_III(a)**–**(c)** (5'TTT TTT TTT TTT-X3', X being the position of the TA residue). Each oligonucleotide was treated with an ammonia solution under three different cleavage conditions: (i) 4 h at room temperature; (ii) 1 h at 55 °C and (iii) overnight at 55 °C. The resulting samples were analyzed by HPLC. Examples of the HPLC profiles of **(T_12_)TA_I** and **(T_12_)TA_III(a)** synthesis crudes are depicted in Figure 2A,B. HPLC profiles of the rest of the samples are shown in the SI (Figure S1). We observed that treatments (i) and (ii) lead to similar HPLC profiles in all cases and the amount of side compound was increased with the prolonged treatment with ammonia at 55 °C (treatment iii). We observed a small increase of side compounds (14% to 17%) in the case of **(T_12_)TA_I**, however, with the other oligonucleotides we detected higher increases of side compounds (25% to 41% for **(T_12_)TA_III(a)**, 33% to 51% for **(T_12_)TA_III(b)** and 24% to 36% for **(T_12_)TA_III(c)**) (Supplementary Information, Table S2). In each case, the main peaks were collected and analyzed by mass spectrometry (Supplementary Information, Table S1). The peak with the highest retention time (t_R_) corresponded with the TA-terminated oligonucleotide. In all cases we observed a compound eluting at t_R_ = 3.2 min. According to the mass analysis this compound was the result of a β-elimination process for samples **(T_12_)TA_I**, **(T_12_)TA_III(a)** and **(T_12_)TA_III(c)** (Figure S1, SI). In contrast, for **(T_12_)TA_III(b)** this same side compound resulted from the amide bond-cleavage. In addition to the hydrolysis products we also observed small amounts of side products eluting between t_R_ = 3.2 min and the main peak. Mass spectrometry analyses of these side compounds (Supplementary Information, Table S1) indicate that they may correspond to different oxidation products of the disulfide group.

Next, we prepared the TA-oligonucleotides required for functionalized AuNPs taking some precautions with **TA_III(a)**–**(c)** analogues. We used the dimethylformamidino group (dmf) to protect guanine in order to minimize the formation of side compounds due to prolonged treatments with ammonia. The DNA analogues **TA_I**, **TA_II** and **TA_III(a)**–**(c)** (5'CGG AGG TAC ATT CGA CTT GAT-X3', X stands for 3'-modifications containing the TA residue) (Figure 1) were prepared using the appropriate solid supports (Scheme 1 and Scheme 2). Finally, compound **12** incorporated into the same sequence at the 5'-end. The coupling efficiency was >90% proving the efficiency of these phosphoramidite (Supplementary Information, Table S3).

After HPLC analysis, all modified oligonucleotides presented a major peak with some impurities, that were around 12%–14% for **TA_I** and **TA_II** and 25%–31% for **TA_III(a)**–**(c)**. The main products were analyzed by mass spectrometry giving the expected mass (Supplementary Information, Table S3). For the AuNPs surface coverage studies of AuNPs a fluorescein was introduced at the 5'-termini of the same sequence obtaining the corresponding fluorescently labeled oligonucleotides **(F)TA_I**, **(F)TA_II** and **(F)TA_III(a)**–**(c)**. All products were purified by HPLC obtaining the desired oligonucleotide as main product (Supplementary Information, Table S3).

We also prepared two additional 3'-thiolated DNA derivatives **ALK_DS** and **ALK_SH**, (5'-CGG AGG TAC ATT CGA CTT GA-Y-3', being Y 3'-linear alkyldisulfide or alkylthiol modifications), which were used as controls and their corresponding fluorescently labeled versions **(F)ALK_DS** and **(F)ALK_SH**. A commercially available solid support, containing the linear alkyldisulfide modification (3'-Thiol modifier C3 S-S CPG; Link Technologies) was used to prepare the oligonucleotide carrying an acyclic disulfide unit at 3'-termini (**ALK_DS**). The fluorescent dye was attached at the 5'-end following the procedure described previously to obtain **(F)ALK_DS**. After HPLC analysis, we observed in both cases a single peak that had the expected mass (Supplementary Information, Table S3). Both oligonucleotides were desalted and used in the next step without further purification. The alkylthiol oligonucleotides **ALK_SH** and **(F)ALK_SH** were obtained from the corresponding alkyldisulfide oligonucleotides by reduction of the disulfide moiety to thiol in the presence of TCEP·HCl following a procedure described in the literature [44,45].

### 2.3. Surface Coverage Quantification and Stability Tests of Oligonucleotide-Gold Nanoparticle Conjugates

In order to study the effect caused by the previously synthesized TA-terminated oligonucleotides on the coverage and stability of gold nanoparticles, all modified oligonucleotides at their 3'-termini along with their fluorescent labeled versions were loaded onto colloidal gold nanoparticles (9.7 nm) by a slow increase of the NaCl concentration and sonication after each saline incremental addition to reach a total salt concentration of 0.15 M (10 mM phosphate, pH 7.04, 0.15 M NaCl, 0.01% NaN_3_) [37,46]. Previously the use of higher salt concentrations (0.3 M NaCl) was described [28,29]. In our hands these protocols are fine with regular alkylthiol group but are not suitable for oligonucleotides carrying lipoic acid moieties as the resulting nanoparticles have a strong tendency to aggregate. Then the number of oligonucleotides loaded on each AuNp was calculated by displacement with a 1 M DTT solution of the corresponding fluorescently labeled oligonucleotides [29,47,48]. By measuring the concentration of oligonucleotides and gold nanoparticle conjugates of each sample, an average number of oligonucleotides per particle was calculated. Then, the average area occupied by an oligonucleotide (footprint) was calculated by dividing the calculated surface area (nm^2^) by the average number of oligonucleotides per particle. Results are summarized in Table 1.

**Table 1:** Surface coverage and half-lives data for oligonucleotide-gold nanoparticle conjugates.

Conjugate	Strands/Particle	Footprint (nm^2^)	t_1/2_ (min)
**AuNP-TA_I**	94 ± 7	3.2 ± 0.2	344 ± 12
**AuNP-TA_II**	93 ± 13	3.2 ± 0.4	282 ± 11
**AuNP-TA_III(a)**	141 ± 10	2.1 ± 0.3	444 ± 8
**AuNP-TA_III(b)**	103 ± 13	2.9 ± 0.4	354 ± 10
**AuNP-TA_III(c)**	102 ± 12	2.8 ± 0.3	450 ± 15
**AuNP-ALK_DS**	40 ± 2	7.4 ± 0.1	4 ± 1
**AuNP_ALK_SH**	97 ± 7	3.0 ± 0.2	12 ± 2

We found similar surface coverage results with **AuNP-TA_I** without any spacer (94 strands/particle) and our control **ALK_SH** (97 strands/particle). However, the functionalization was less efficient for AuNP-**ALK_DS** (40 strands/particle). A possible explanation of this result could be the larger anchoring group present in **ALK_DS** oligonucleotide compared to **ALK_SH**. Then, the ability of the dialkyldisulfide group to form dense layers would be lower than the alkylthiol group. In addition, it is described that the adsorption of a dialkyldisulfide is possible, but thermodinamically not stable [49]. The process implies the cleavage of the disulfide bond, which generates two monoalkylthiolated branches, that do not adsorb adjacent to each other [50]. Maybe the monolayer obtained in this case is not so uniform compared with the monolayer formed with **ALK_SH** oligonucleotide in terms of thiolated species adsorbed onto the surface explaining the reduction in the coverage efficiency.

We observed different trends depending on the linker nature and how it has been incorporated. **AuNP-TA_I** and **AuNP-TA_II** showed similar surface coverage results. In this case, the introduction of a hexaethylene glycol spacer did not lead to a higher coverage. Maybe, the threoninol and the phosphate groups, which are placed very close to the surface, are preventing to achieve a higher loading due to steric and electrostatic effects. In the case of the TA_III series, a slight increase in the ligand density was observed when a spacer similar in length (alkyl or a triglycine peptide) was introduced. A higher density of ligand was observed when an oligo(ethylene glycol) was introduced between the TA group and the oligonucleotide (141 strands/particle), that is an increase of around 50% on DNA loading compared with **AuNP-TA_I** conjugates. This result indicates the beneficial effect of the spacer and is in good agreement with other results published in the literature [37], where thiolated oligonucleotides with and without an oligo(ethylene glycol) spacer were used to functionalize AuNP. The authors reasoned that the spacer moves the DNA tract away from the gold surface thus reducing the interstrand repulsion between negatively charged phosphate/sugar backbones and as well the tendency of DNA nucleobases to interact with the gold surface. An important characteristic to take into account regarding monolayers formed on the surface of AuNPs is the radius of curvature. A consequence of this curvature is a decrease in the chain density moving away from the surface of the core. The decreasing density translates into an enhanced mobility of the terminal tracts of a ligand chain [51]. For the TA-terminated DNA oligonucleotides the decreasing density seems to minimize the interstrand repulsions of the negatively charged tract. It could be a plausible explanation for the higher density of packing obtained with all three **AuNp-TA_III** conjugates. 

**Figure 3:**
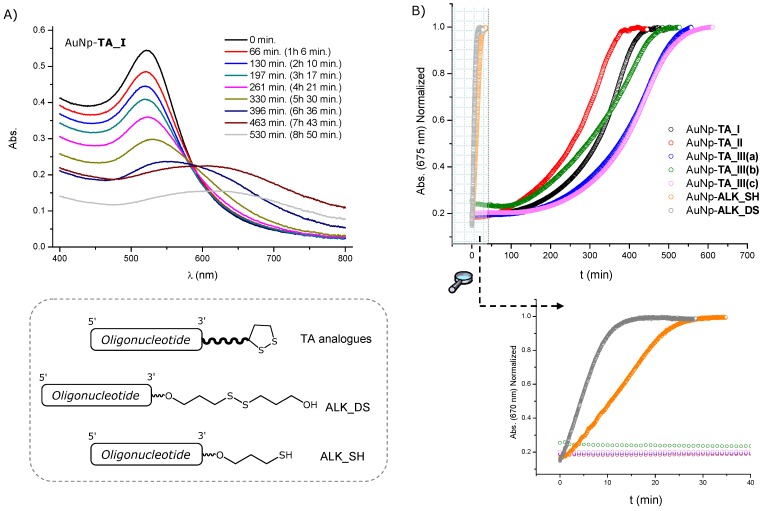
(**A**) Time evolution of UV-Vis spectra of **AuNp-TA_I** in 100 mM DTT; (**B**) Displacement of modified TA-appended oligonucleotides from AuNPs by measuring the changes in signal intensity at 675 nm with time upon treatment with a 100 mM DTT solution.

Next, we studied the stability of our AuNP-conjugates against ligand exchange in the presence of DTT. At high concentration, DTT can displace thiolated ligands, resulting in progressive aggregation of the AuNPs. Then, the ability of the spacers to provide additional shielding of the inorganic core was evaluated by assessing the aggregate formation in the presence of DTT (100 mM) at 40 °C. Figure 3A shows the UV-Vis spectra taken at different times for **AuNP-TA_I**. During time evolution of the UV-Vis spectra, a decay of the plasmon peak of colloidal gold (522 nm) was observed, while the intensity between 600 and 700 nm increased. Then, the displacement of oligonucleotides from AuNPs was monitored by measuring the changes in signal intensity at 675 nm with time (Figure 3B). The time required to reach half of the maximum intensity (t_1/2_) was used to compare the stability of each AuNP-conjugate. Table 1 summarizes the results obtained in each case. Comparing the conjugates formed with the TA series with the conjugates formed with oligonucleotides containing an alkylthiol or an acyclic alkyldisulfide a great difference in the stability was observed. The complete aggregation occurred at 15 min and 30 min for **AuNp-ALK_DS** and **AuNp_ALK_SH** respectively. This is in good agreement with other results published in the literature [22,29,52], where oligonucleotides were modified with cyclic disulfides to functionalize AuNPs. In these cases a large increase in the stability was observed in comparison with AuNPs functionalized with monoalkylthiol- or acyclic disulfide-oligonucleotides, suggesting that those differences might be due to different binding affinities of the different thiolated groups to gold surfaces. 

Focusing on the results obtained with conjugates formed with TA-terminated oligonucleotides, we found that conjugate formed with **TA_I** (without a spacer) showed a high stabilization in the presence of DTT (t_1/2_ 344 min.). The conjugates formed with **TA_III(a)** and **TA_III(c)**, (containing an oligo(ethylene glycol)-like molecule and an alkyl group as spacer respectively) were very similar and showed a clear increase of about 100 min in t_1/2_ compared with conjugates formed with **TA_I**. On the other hand, when the spacer was a triglycine peptide (**TA_III(b)**), the t_1/2_ value obtained was very similar to conjugates formed with **TA_I**. Finally, the conjugates formed with **TA_II** containing hexaethylene glycol as a spacer) showed a clear decrease (62 min) in t_1/2_ when compared with **TA_I**.

The replacement rate of thiolated ligands depends on a number of parameters such as the packing density, the order degree or the intermolecular interactions between the ligands [53]. In our case, a high packing density not necessarily provides an enhanced stability towards thiol replacement. Interestingly, **AuNP-TA_I** and **AuNP_TA_III(b)** showed comparable t_1/2_ values whereas **AuNP-TA_I** resulted to be more stable than **AuNP-TA_II**. The results obtained could be rationalized as follows: the stability of the AuNP conjugates functionalized with TA-terminated oligonucleotides towards thiol replacement is driven by the thermodynamic stability of the anchoring group and by the shielding that the peripheral groups provide to the gold core. In the case of **AuNP-TA_I** the negatively charged and bulky oligonucleotide chain lays very close to the cluster surface, thus providing steric hindrance to the approaching thiol. When a spacer is added, the oligonucleotide chain is moved further away, then reducing the interstrand repulsions due to the radial distribution of the molecules attached to AuNPs. Then the shielding efficiency towards ligand exchange relies on the packing quality of the spacer.

**Figure 4:**
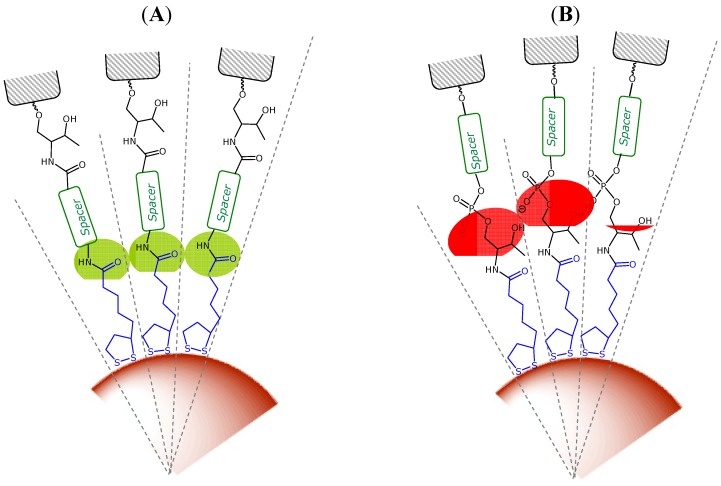
Scheme showing (**A**) favourable interactions (in green) between amide groups and (**B**) disfavourable interactions (in red) between bulky or negatively charged substituents.

A characteristic of AuNP cores is that they have faceted surfaces, which generate a radial structure for the covering monolayer [51]. These authors functionalized AuNPs with different alkylthiols containing an amide group at different positions. They found that the interactions between amide groups were highly dependent on the distance to the AuNP core [54]. In light of our results, **AuNp-TA_III(c)** and **AuNp-TA_III(a)** conjugates gave the highest rates of half-lives compared to the rest of the series, showing practically the same stability pattern. It can be reasoned that the amide bond could play an important role in this higher stability by providing an increase in monolayer order through hydrogen bonding interactions that leads t_1/2_ values to enhanced interactions between the neighboring alkyl or ethylene glycol chains (Figure 4). In the case of the **AuNP-TA_III(b)** conjugate, we observed similar t_1/2_ values compared with **AuNP-TA_I**. The effect on the stability of this spacer could be explained as the ability of amide groups to participate in hydrogen-bonding interactions is decreased when the distance to the surface increases. Finally, the lower stability observed with the **AuNP-TA_II** could be attributed to the bulky groups near the amide bond (threoninol group and phosphate linkage) (Figure 4). These groups may prevent effective hydrogen bonding interactions due to steric and electrostatic factors that could affect the packing quality of the spacer leading to a decrease in the interactions between the oligo(ethylene glycol) chains.

### 2.4. Stability of the Thioctic Acid Derivatives to Cathepsin

AuNPs are potential vehicles for drug delivery [55]. One of the potential applications of the oligonucleotide-AuNPs described in this work is their use in gene silencing. In addition of a dense and stable functionalization of the nanoparticles, it will be important to be able to break the linker between oligonucleotide and nanoparticle to release the cargo inside the cells. It is reported that the triglycine linker has been used for the endosomal escape of several drugs such as DOTA, doxorubicin and T-2513 [33,34,35] because of the lability to cathepsin. Then, we decided to analyze if the same lability was present in the TA-oligonucleotides described in this work. 

**Figure 5:**
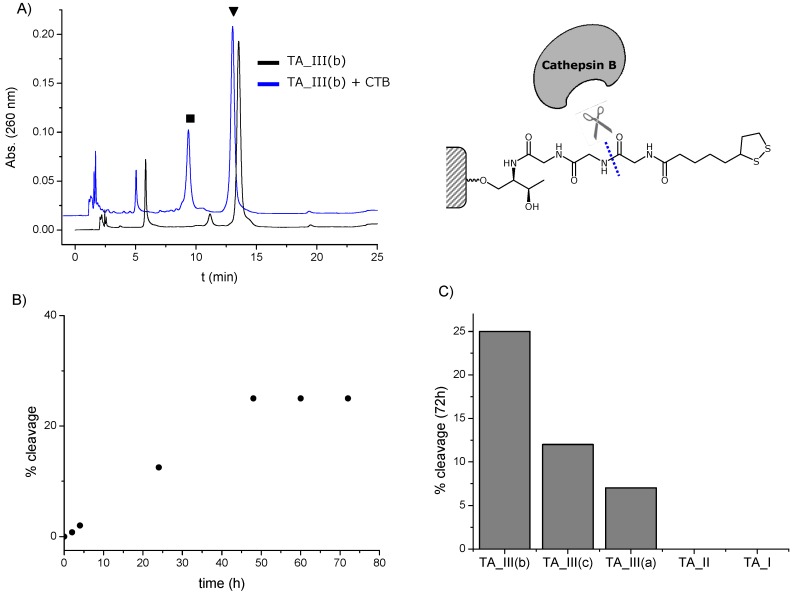
(**A**) HPLC profiles obtained with **TA_III(b)** after 72 h in the presence (blue line) and in the absence (black line) of cathepsin B (CTB). ▼ stands for **TA_III(b)** and ■ stands for the product resulting from the amide-bond cleavage; (**B**) % amide-bond cleavage of **TA_III(b)** with time; (**C**) % amide-bond cleavage after 72 h of different TA-terminated oligonucleotides.

Our study was focused in the determination the specificity of cathepsin B for the triglycine TA spacer (**TA_III**) *versus* the other TA modified oligonucleotides (**TA_I** and **TA_II**). The TA conjugates were incubated with Cathepsin B for 72 h and analyzed by HPLC to elucidate the enzymatic response (Figure 5A and Figure Supplementary Information, S5). The HPLC profiles for the **TA_III** conjugates showed the appearance of a peak with shorter retention time, which corresponded to the cleavage product once analyzed by mass spectrometry (Figure 5A blue line, Supplementary Information, Table S4), whereas in the case of the **TA_I** and **TA_II** this peak was absent (Supplementary Information, Figure S5). These results confirmed that cathepsin B was able to cleavage the amide bonds oligonucleotides from the **TA_III** conjugates, however it was unable to liberate the oligonucleotide from **TA_I** and **TA_II** conjugates. That indicates that the activity of the enzyme is affected by the topology around the amide bond. To consolidate these results we investigated the effect of the proteinase over time with the **TA_III(b)**. Figure 5B shows the increase of product release over time until a plateau was reached at 72 h as expected. Moreover, we could observe a different cleavage efficiency of cathepsin B between the three spacers present in the **TA_III** series (Figure 5C). We obtained the higher cleavage efficiency with the triglycine spacer (25%) followed by the alkyl chain and the lower cleavage was for the oligo(ethylene glycol) molecule. These results are consistent with the fact that the triglycine spacer has a peptide-like nature compared with the other two linkers. 

## 3. Experimental Section

### General Methods and Materials

All reagents were purchased from Sigma-Aldrich Spain (Tres cantos, Spain) or Fluka (Sigma-Aldrich Química S.A., Spain) and were used without further purification. Dry solvents were purchased as well from Sigma-Aldrich or Fluka and used as supplied. All reactions were carried out under inert atmosphere of argon. All the standard phosphoramidites and ancillary reagents used for oligonucleotide synthesis were purchased from Applied Biosystems (Foster City, CA, USA) or Link Technologies (Glasgow, Scotland, UK). The benzoyl (Bz) group was used for the protection of the amino group of C and A. For the protection of G we used either the isobutyryl (^i^Bu) group in the case of **TA_I** and **TA_II** and the dimethylformamidino group (dmf) in the case of **TA_III(a)**–**(c)**. The hexaethylene glycol phosphoramidite (spacer phosphoramidite C18) was purchased from Glen Research (Sterling, VA, USA). The 6'-Fluorescein-CE-phosphoramidite and the 3'-thiol modifier C3 S-S CPG were purchased from Link Technologies. The oligonucleotides were desalted with Sephadex G-25 (NAP-10 or NAP-5 column, from GE Healthcare Spain (Barcelona, Spain). Cathepsin B was purchased from Sigma-Aldrich. Aqueous solution of citrate stabilized gold nanoparticles (9.7 nm) were purchased from BBI Life Sciences (Cardiff, UK) and used as received. Flash column chromatography was carried out on silica gel SDS 0.063–0.2 mm/70–230 mesh. ^1^H- and ^13^C-NMR spectra were recorded at 25 °C on a Varian Mercury 400 MHz spectrometer (Agilent Tehcnologies, Santa Clara, CA, USA). ^31^P-NMR spectra were recorded at room temperature on a Varian Mercury 400 MHz, operating at 162 MHz, using an internal reference using deuterated solvents. Tetramethylsilane (TMS) was used as an internal reference (0 ppm) for ^1^H spectra recorded in CDCl_3_ and the residual signal of the solvent (77.16 ppm) for ^13^C spectra. For CD_3_OD or d_6_-DMSO the residual signal of the solvent was used as a reference. Chemical shifts are reported in part per million (ppm) in the δ scale, coupling constants (J) in Hz and multiplicity as follows: s (singlet), d (doublet), t (triplet), q (quadruplet), quint (quintuplet), m (multiplet) and br (broad signal). IR Spectra were recorded on a BOMEM MB-120 spectrometer (ABB, Frankfurt am Main, Germany). HPLC analysis and separations were performed using a Waters 2695 Separations Module (Milford, MA, USA) equipped with a Waters 2998 Photodiode Array Detector. The HPLC solvent used where: A: 5% ACN in 100 mM triethylammonium acetate (TEAA) (pH = 7) and solvent B: 70% ACN in 100 mM TEAA (pH = 7). Oligonucleotides synthesis was done in a DNA/RNA synthesizer (Applied Biosystems, Foster City, CA, USA) and characterized by mass spectrometry (MALDI-TOF). UV analyses were performed using a Jasco V-650 instrument (Easton, MD, USA) equipped with a thermoregulated cell holder. Fluorescent measures were recorded on a Jasco FP-6200 spectrofluorometer equipped as well with a thermoregulated cell holder. Electrospray ionization mass spectra (ESI-MS) were recorded on a Micromass ZQ instrument (Milford, MA, USA) with single quadrupole detector coupled to an HPLC, and high-resolution (HR) ESI-MS on an Agilent 1100 LC/MS-TOF instrument (Santa Clara, CA, USA). Matrix-assisted laser desorption ionization time-of-flight (MALDI-TOF) mass spectra were recorded on a Voyager-DETMRP spectrometer (Applied Biosystems) in negative mode (2,4,6-trihidroxyacetophenone matrix with ammonium citrate as an additive).

### 3.1. 4-(Dithiolan-3-yl)-N-[(1R,2R)-2-hydroxy-1-(hydroxymethyl)propyl]butanamide (**1**)

The compound was obtained as a mixture of two diastereoisomers. A solution of (±)-α-lipoic acid (344 mg, 1.67 mmol), (2*R*,3*R*)-2-aminobutane-1,3-diol (175 mg, 1.67 mmol), 1-ethyl-3-(3-dimethylaminopropyl)carbodiimide (EDCI, 447 mg, 2.50 mmol), hydroxybenzotriazole (HOBt, 339 mg; 2.50 mmol) and N,N-diisopropylethylamine (DIEA, 433 µL, 2.50 mmol) in anhydrous dimethylformamide (DMF, 15 mL) was prepared under argon. After stirring the reaction mixture at room temperature overnight, the reaction was complete as judged by TLC and then concentrated to dryness under reduced pressure. In order to remove the remaining DMF the residue was dissolved in toluene and concentrated to dryness under reduced pressure (×3). The residue was dissolved in 100 mL of dichloromethane (DCM) and the organic phase was washed with 50 mL 10% NaHCO3 aqueous solution (×2) and 50 mL of a saturated NaCl aqueous solution. The organic layer was dried over anhydrous magnesium sulfate and concentrated to dryness under reduced pressure. The product was purified by flash chromatography using as eluent pure DCM to DCM with 4% MeOH to give compound **1** (371 mg, 76% yield). IR (film): 3320, 2927, 1643, 1539 cm^−1^. ^1^H-NMR (400 MHz, CDCl_3_): δ_H_ = 6.21 (br, 1H, NH), 4.19 (qd, *J* = 6.3 Hz, 1.57 Hz; 1H, OH-CH-CH_3_), 3.84 (m, 2H, CH-CH_2_-OH), 3.57 (quint, *J* = 6.5 Hz, 1H, N-CH-CH_2_), 3.14 (m, 2H, S-CH_2_-CH_2_-), 2.46 (m, 1H, S-CH-CH_2_-), 2.28 (t, *J* = 7.4 Hz, 2H, CO-CH_2_-), 1.92 (m, 2H, -CH_2_-CH_2_-), 1.70 (m, 4H, -CH_2_-CH_2_-), 1.49 (m, 2H, CH-CH_2_-CH_2_-), 1.21 (d, *J* = 7.1 Hz, 3H, CH_3_-CH) ppm.^13^C-NMR (125 MHz, CDCl_3_): δ_C_ = 173.92 (NH-CO-), 69.41 (CH-O-), 65.62 (CH_2_-O), 56.61 (N-CH-CH_2_-), 54.63 (CH_2_-CO), 40.47 (CH_2_-S), 38.70 (CH_2_-CH-S), 36.71, 34.82, 29.01, 25.63 (alkyl chain), 20.94 (CH_3_-CH) ppm. HRMS (ESI+): *m/z* calcd for C_12_H_24_NO_3_S_2_ ([M + H]^+^) 294.1199 found 294.1192; *m/z* calcd for C_24_H_46_N_2_NaO_6_S_4_ ([2M + Na]^+^) 609.2138 found 609.2131.

### 3.2. General Procedure for the Synthesis of Amides **7a**–**7c**

The corresponding carboxylic acid (2.11 mmol) along with 4-nitrophenol (353 mg, 2.54 mmol) were dissolved in anhydrous pyridine (15 mL) under argon and the solution was cooled at 0 °C. Then, 524 mg (2.54 mmol) of 1,3-dicyclohexylcarbodiimide (DCC) were added under argon and the reaction was stirred at 0 °C for 30 min and at room temperature overnight. The precipitate was filtered out and the solvent was removed under reduced pressure. The residue was dissolved in toluene and concentrated to dryness under reduced pressure (×3). The resulting p-nitrophenyl esters **5a**–**5c** were used in the next step without further purification. Then, the activated acids along with (2*R*,3*R*)-2-aminobutane-1,3-diol (L-threoninol) (244 mg, 2.32 mmol) were dissolved in anhydrous DMF (15 mL) under argon and allowed to react at room temperature for 3 h. The solvent was removed under reduced pressure and the residue was dissolved in toluene and concentrated to dryness (×3). Crude compounds **7a** and **7c** were dissolved in 100 mL of DCM and the organic phase was washed with 50 mL 10% NaHCO_3_ aqueous solution (×2) and 50 mL of a saturated NaCl aqueous solution. The organic layers were dried with anhydrous MgSO_4_ and the corresponding crudes were purified by flash chromatography on silica gel using as solvent pure DCM to DCM with 4% MeOH. Crude of compound **7b** once dried with toluene was directly purified by flash chromatography on silica gel (crude adsorbed onto the silica, DCM/MeOH 100:5→100:15).

#### 3.2.1. *tert-Butyl-N-[3-[2-[2-[3-[[4-[[(1R,2R)-2-hydroxy-1-(hydroxymethyl)propyl]amino]-4-oxo-butanoyl]amino]propoxy]ethoxy]ethoxy]propyl] carbamate* (**7a**)

Yield: 738 mg (69%). IR (film): 3325, 2930, 2871, 1690, 1654, 1365, 1170, 1103 cm^−1^. ^1^H-NMR (400 MHz, CDCl_3_): δ_H_= 6.75 (br, 1H, N*H*), 5.07 (br, 1*H*, O*H*), 4.14 (dq, *J* = 6.3 Hz, 2.5 Hz; 1H, OH-C*H*-CH_3_), 3.84 (dd, *J* = 11.5 Hz, 3.5 Hz, 1H, CH-C*H*-OH), 3.75 (m, 2H, C*H*-C*H*-OH), 3.65 (m, 4H, O-C*H_2_*-C*H_2_*-O), 3.59 (m, 4H, O-C*H_2_*-C*H_2_*-O), 3.55–3.47 (m, 4H, CH_2_-C*H_2_*-O and O-C*H_2_*-CH_2_), 3.35 (m, 2H, NH-C*H_2_*-), 3.21 (m, 2H, NH-C*H_2_*), 2.61–2.54 (m, 4H, NH-CH_2_-C*H_2_*-CH_2_-O), 1.75 (2t, 4H, CO-C*H**_2_*-C*H_2_*-CO), 1.43 (s, 9H, (C*H_3_*)_3_-C), 1.19 (d, *J* = 6.4 Hz, 3H, C*H_3_*-CH) ppm.^13^C-NMR (125 MHz, CDCl_3_): δ_C_ = 173.54 (*C*O), 172.72 (*C*O), 172.62 (*C*O), 77.43 (CH_3_)_3_-*C*-O), 70.62 (O-*C*H_2_-CH_2_-O), 70.30 (O-CH_2_-*C*H_2_-O), 70.29 (O-CH_2_-*C*H_2_-O), 69.63 (O-*C*H_2_-CH_2_-O), 69.59 (*C*H_2_-O), 69.49 (*C*H-O-), 64.93 (*C*H_2_-O), 64.86 (*C*H_2_-O), 55.63 (N-*C*H-CH_2_), 55.62 (*C*H_2_-N), 51.03 (*C*H_2_-N), 32.44 (*C*H_2_-CO), 32.16 (*C*H_2_-CO), 29.93 (*C*H_2_-CH_2_), 28.98 (CH_2_-*C*H_2_), 28.62 (*C*H_3_-C), 20.57 (*C*H_3_-CH) ppm. HRMS (ESI+): *m/z* calcd for C_23_H_46_N_3_O_9_ ([M + H]^+^) 508.3229 found 508.3231; *m/z* calcd for C_23_H_45_N_3_NaO_9_ ([M + Na]^+^) 530.3048 found 530.3050.

#### 3.2.2. *N-[N'-(tert-Butoxycarbonyl)glycylglycylglycyl]-l-threoninol* (**7b**)

Yield: 612 mg (78%). ^1^H-NMR (400 MHz, DMSO-*d*_6_): δ_H_ = 8.09–8.03 (m, 2H, N*H*), 7.27 (d, *J* = 8.8 Hz, 1H, N*H*), 6.97 (t, *J* = 5.6 Hz, 1H, N*H*), 4.61–4.54 (br, 2H, O*H*), 3.85–3.81 (m, 1H, OH-C*H*-CH_3_), 3.72–3.70 (m, 4H, NH-C*H_2_*-CO), 3.63–3.58 (m, 2H, NH-C*H*-CH_2_), 3.56 (d, *J*=6.0 Hz, 2H, NH-C*H_2_*-CO), 3.44–3.40 (m, 1H, OH-C*H_(A)_*H_(B)_-CH)), 3.33–3.28 (m, 1H, OH-CH_(A)_*H_(B)_*-CH)),1.36 (s, 9H, (C*H_3_*)_3_-C), 0.97 (d, *J* = 6.4 Hz, 3H, C*H_3_*-CH-CH) ppm.^13^C-NMR (125 MHz, DMSO-d_6_): δ_C_ = 170.58 (NH-*C*O), 169.67 (NH-*C*O), 169.37 (NH-*C*O), 156.50 (O-*C*O-NH), 78.83 (CH_3_-*C*), 64.88 (CH_3_-*C*H), 61.12 (OH-*C*H_2_-CH), 56.43 (NH-*C*H-CH), 43.99 (NH-*C*H_2_-CO), 42.77 (NH-*C*H_2_-CO), 42.70 (NH-*C*H_2_-CO), 28.87 (*C*H_3_-C), 20.73 (*C*H_3_-CH). HRMS (ESI+): *m/z*: calcd. for C_15_H_29_N_4_O_7_([M + H]^+^) 377.2036 found 377.2031; *m/z*: calcd. for C_15_H_28_N_4_NaO_7_ ([M + Na]^+^) 399.1856 found 399.1856.

#### 3.2.3. *tert-Butyl-N-[8-[[(1R,2R)-2-hydroxy-1-(hydroxymethyl)propyl]amino]-8-oxo-octyl]carbamate* (**7c**)

Yield: 701 mg (96%). IR (film): 3323, 2974, 2930, 2858, 1689, 1652, 1525, 1366, 1251, 1171 cm^−1^. ^1^H-NMR (400 MHz, CDCl_3_): δ_H_ = 6.30 (br, 1H, N*H*), 4.56 (br, 1H, O*H*), 4.19 (qd, *J* = 6.4 Hz, 1.9 Hz, 1H, OH-C*H*-CH_3_), 3.83 (m, 3H, C*H*-C*H_2_*-OH), 3.07 (m, 2H, C*H_2_*-NH), 2.26 (t, *J* = 7.3 Hz, 2H, C*H_2_*-CH_2_), 1.66 (m, 2H, alkyl chain), 1.44 (s, 9H, (C*H_3_*)_3_-C), 1.33 (m, 6H, alkyl chain), 1.21 (d, *J* = 6.3 Hz, 3H, C*H_3_*-CH) ppm. ^13^C-NMR (125 MHz, CDCl_3_): δ_C_ = 174.31 (*C*O-NH), 77.43 (CH_3_-*C*-O), 69.43 (*C*H-O), 65.62 (*C*H_2_-O), 54.61 (N-*C*H-CH_2_), 40.63 (*C*H_2_-CO), 36.86, 29.94, 29.04, 28.62, 26.54, 25.57 (alkyl chain), 20.82 (*C*H_3_-CH-) ppm. HRMS (ESI+): *m/z* calcd for C_17_H_35_N_2_O_5_ ([M + H]^+^) 347.2540 found 347.2543; *m/z* calcd for C_34_H_68_N_4_NaO_10_ ([2M + Na]^+^) 715.4828 found 715.4824.

### 3.3. General Procedure for the Introduction of Lipoic Acid Residue: Synthesis of Compounds **8a**–**8c**

Using the same experimental procedure as described above for the activation of carboxylic acids, a solution of (±)-α-lipoic acid (322 mg, 1.56 mmol) and *p*-nitrophenol (239 mg, 1.72 mmol) in anhydrous pyridine (15 mL) was prepared under argon. Then, the solution was cooled to 0 °C and DCC (354 mg, 1.72 mmol) was added under argon. The reaction was stirred at 0 °C for 30 min and at room temperature overnight. The precipitate was filtered out and the solvent was removed under reduced pressure. The residue was dissolved in toluene and concentrated to dryness under reduced pressure (×3). Then the crude was dissolved in DCM (75 mL) and the organic phase was washed with 5% aqueous NaHCO_3_ (2 × 30 mL) and saturated aqueous NaCl (30 mL). The organic phase was dried (MgSO_4_) and concentrated to dryness under reduced pressure and then dried in a desiccator. The *p*-nitrophenyl ester **6** (yellow solid) was used in the next step without further purification. Then, the corresponding protected amines **7a**–**7c** (1.30 mmol) were subjected to acidic conditions (DCM/TFA 10%) in order to obtain the respective free amines as trifluoroacetate salts. Each protected amine was dissolved in DCM (20mL) and TFA (2 mL) was added slowly at room temperature. Stirring was continued until TLC showed the consumption of all starting material (usually 1–1.5 h), then the mixture was concentrated to dryness under reduced pressure. The residue was dissolved in toluene and concentrated to dryness under reduced pressure. This operation was repeated three times to remove traces of TFA to yield the TFA salt of the deprotected amino compound (colorless oil). The residue was dried *in vacuo* and used in the next step without further purification. Then, each deprotected amino compound was dissolved in anhydrous DMF (10 mL) and anhydrous DIEA (0.94 mL, 5.20 mmol) was added under argon. The solution was added dropwise under argon to a prepared solution (anhydrous DMF, 10 mL) of the activated lipoic acid derivative. The mixture was allowed to react at room temperature and under argon overnight. The solvent was removed under reduced pressure and the residue was dissolved in toluene (3 × 10 mL) and concentrated to dryness. Crudes of compounds **8a** and **8c** were dissolved into 75 mL of dichloromethane and were washed with 5% NaHCO_3_ (3 × 30 mL) and with a saturated aqueous solution NaCl (30 mL). The organic layers were dried with anhydrous MgSO_4_ and the corresponding crudes were purified by flash chromatography on silica gel using as solvent pure DCM to DCM with 4% MeOH. Crude of **8b** once dried with toluene was directly purified by flash chromatography on silica gel (crude adsorbed onto the silica, DCM/MeOH 100:5→100:15).

#### 3.3.1. *N'-[3-[2-[2-[3-[4-(Dithiolan-3-yl)butanoylamino]propoxy]ethoxy]ethoxy]propyl]-N-[(1R,2R)-2-hydroxy-1-(hydroxymethyl)propyl]butanediamide* (**8a**)

The compound was obtained as a mixture of two diastereoisomers. Yield: 565 mg (73%). IR (film): 3320, 2928, 1652, 1539, 1456, 1216, 1130 cm^−1^. ^1^H-NMR (400 MHz, CDCl_3_): δ_H_ = 6.73 (br, 1H, N*H*), 6.65 (br, 1H, O*H*), 6.43 (br, 1H), 4.16 (dq, *J* = 6.3 Hz, 2.6 Hz, 1H, OH-C*H*-CH_3_), 3.88 (dd, *J* = 11.3 Hz, 3.4 Hz, 1H, CH-C*H*-OH), 3.75 (m, 2H, C*H*-C*H*-OH), 3.65 (m, 4H, O-C*H_2_*-C*H_2_*-O), 3.61 (m, 4H, O-C*H_2_*-C*H_2_*-O), 3.55 (m, 4H, CH_2_-C*H_2_*-O and O-C*H_2_*-CH_2_), 3.35 (m, 4H, 2 NH-C*H_2_*-), 3.18 (m, 2H, S-C*H_2_*-CH_2_), 3.14 (m, 2H, C*H_2_*-NHCO), 2.64 (m, 1H, S-C*H*-CH_2_), 2.49 (m, 4H, NH-CH_2_-C*H_2_*-CH_2_-O), 2.17 (t, *J* = 7.6 Hz, 2H, C*H_2_*-CH_2_-), 1.74 (m, 8H, CO-C*H_2_*-C*H_2_*-CO and alkyl residue), 1.44 (d, *J* = 6.9 Hz, 3H, C*H_3_*-CH), 1.38 (d, *J* = 6.7 Hz, 3H, C*H_3_*-CH) ppm.^13^C-NMR (125 MHz, CDCl_3_): δ_C_ = 173.72 (*C*O), 173.35 (*C*O), 172.51 (*C*O), 70.73 (O-*C*H_2_-CH_2_-O), 70.62 (O-CH_2_-*C*H_2_-O), 70.20 (O-CH_2_-*C*H_2_-O), 70.19 (O-CH_2_-*C*H_2_-O), 70.18 (*C*H_2_-O), 70.08 (*C*H_2_-O), 68.89 (*C*H-O), 64.54 (*C*H_2_-O), 56.75 (CH_2_-CO), 55.71 (CH_2_-CO), 41.86 (CH_2_-*C*H-N), 40.43 (*C*H_2_-S), 38.62 (CH_2_-N), 38.16 (CH_2_-N), 37.82 (CH_2_-*C*H-S), 36.67 (CH_2_-CO), 34.88 (S-CH_2_-*C*H_2_-CH-S) 37.85 (S-CH-*C*H_2_), 36.63 (-*C*H_2_-CH_2_-), 34.85 (-CH_2_-*C*H_2_-), 32.31 (NH-CH_2_-*C*H_2_-), 32.21 (-CH2-*C*H_2_-CH_2_-O), 32.09, 29.34, 29.05 (*C*H_3_-CH) ppm. HRMS (ESI+): *m/z* calcd for C_26_H_50_N_3_O_8_S_2_ ([M + H]^+^) 596.3034, found 596.3038.

#### 3.3.2. *N-[N'-{5-(1,2-Dithiolan-3-yl)pentanoyl}glycylglycylglycyl]-l-threoninol* (**8b**)

The compound was obtained as a mixture of two diastereoisomers. Yield: 459 mg (76%). ^1^H-NMR (400 MHz, CD_3_OD): δ_H_ = 3.91–3.86 (m, 1H, OH-C*H*-CH_3_), 3.82–3.81 (m, 2H, NH-C*H_2_*-CO), 3.79 (d, *J* = 2.8 Hz, 4H, NH-C*H_2_*-CO), 3.74–3.70 (m, 1H, NH-C*H*-CH_2_), 3.58–3.53 (m, 1H, OH-C*H_(A)_*H_(B)_-CH), 3.51–3.46 (m, 1H, OH-CH_(A)_*H_(B)_*-CH), 3.13–2.97 (m, 3H, S-C*H_2_*-CH_2_ and S-C*H*-CH_2_), 2.41–2.33 (m, 1H, S-CH-C*H_(A)_*H_(B)_-CH_2_), 2.20 (t, *J* = 7.4 Hz, 2H, CH_2_-C*H_2_*-CO-NH), 1.84–1.76 (m, 1H, S-CH-CH_(A)_*H_(B)_*-CH_2_), 1.68–1.50 (m, 4H, alkyl chain), 1.42–1.35 (m, 2H, alkyl chain), 1.05 and 1.04 (2d, *J* = 6.4 Hz and *J* = 6.4 Hz, 3H, C*H_3_*-CH) ppm. ^13^C-NMR (125 MHz, DMSO-*d*_6_): δ_C_ = 173.30 (NH-*C*O), 170.36 (NH-*C*O), 169.70 (NH-*C*O), 169.41 (NH-*C*O), 64.91 (CH_3_-*C*H), 61.13 (OH-*C*H_2_-CH), 56.79 (NH-*C*H-CH_2_-), 56.45 (S-*C*H-CH_2_), 46.40 (S-*C*H_2_-CH_2_), 43.96 (NH-*C*H_2_-CO), 42.74 (NH-*C*H_2_-CO), 42.72 (NH-*C*H_2_-CO), 38.77 (S-CH-*C*H_2_), 35.63 (CH_2_-*C*H_2_-CO-NH), 34.81 (*C*H_2_ alkyl chain), 29.01 (*C*H_2_alkyl chain), 25.5 (*C*H_2_ alkyl chain), 20.72 (*C*H_3_-CH) ppm. HRMS (ESI+): *m/z* calcd for C_18_H_33_N_4_O_6_S_2_ ([M + H]^+^) 465.1842, found 465.1848; *m/z* calcd for C_18_H_32_N_4_NaO_6_S_2_ ([M + Na]^+^) 487.1661 found 487.1658.

#### 3.3.3. *8-[4-(Dithiolan-3-yl)butanoylamino]-N-[(1R,2R)-2-hydroxy-1-(hydroxymethyl)propyl]octan-amide* (**8c**)

The compound was obtained as a mixture of two diastereoisomers. Yield: 231 mg (41%). IR (film): 3304, 2927, 2855, 1646, 1557, 1540, 1457, 1201 cm^−1^. ^1^H-NMR (400 MHz, CDCl_3_): δ_H_ = 6.37 (br, 1H, N*H*), 5.59 (br, 1H; O*H*), 4.18 (qd, *J* = 6.2 Hz, 1.5 Hz, 1H, OH-C*H*-CH_3_) 3.84 (m, 2H, CH-C*H_2_*-OH), 3.57 (m, 1H, C*H*-CH_2_-OH), 3.23 (m, 2H, S-C*H_2_*-CH_2_-), 3.13 (m, 2H, C*H_2_*-NHCO), 2.47 (m, 1H, S-C*H*-CH_2_-), 2.27 (d, *J* = 7.3 Hz, 2H, -C*H_2_*-CH_2_, lipoic residue), 2.17 (t, *J* = 7.0 Hz, 2H, -C*H_2_*-CH_2_-), 1.91 (m, 2H, -C*H_2_*-CH_2_-), 1.67 (m, 6H, alkyl chain), 1.48 (m, 2H, alkyl chain), 1.33 (m, 8H, alkyl chain), 1.21 (d, *J* = 6.4 Hz, 3H, C*H_3_*-CH) ppm.^13^C-NMR (125 MHz, CDCl_3_): δ_C_ = 174.45 (*C*ONH), 173.44 (*C*ONH), 69.69 (*C*H-O-), 65.72 (*C*H_2_-O), 56.62 (NH-*C*H-CH_2_-), 54.65 (NH-*C*H_2_), 45.85 (*C*H_2_-CO), 40.47 (*C*H_2_-CO), 39.55 (*C*H_2_-S), 38.72 (CH_2_-*C*H-S), 36.81, 34.72, 29.42, 29.07, 28.76, 28.40, 28.39, 26.37, 25.72 (alkyl chain), 20.72 (*C*H_3_-CH) ppm. HRMS (ESI+): *m/z* calcd for C_20_H_39_N_2_O_4_S_2_ ([M + H]^+^) 435.2346, found 435.2351; *m/z* calcd for C_40_H_76_N_4_NaO_8_S_4_ ([2M + Na]^+^) 869.4619 found 869.4615.

### 3.4. General Procedure for the Preparation of the DMT Protected Compounds **2** and **9a**–**9c**

Each diol (0.80 mmol) was dried by evaporation of anhydrous acetonitrile (ACN) under reduced pressure (×2). Then to a solution of the corresponding diol in anhydrous pyridine (15 mL) at 0 °C, DIEA (217 µL, 1.20 mmol), 4,4'-dimethoxyltritylchloride (DMT-Cl) (297 mg, 0.88 mmol) and dimethylaminopyridine (DMAP) (catalytic amount) were added. After 15 min, the mixture was allowed to reach room temperature. Then the reaction was stirred for an additional 24 h and finally was quenched with methanol except for diol **7b**. In this case DMT-Cl (135 mg, 0.40 mmol), DIEA (109 µL 0.60 mmol) and DMAP (catalytic amount) were dissolved in dry pyridine (5 mL) and were added to the mixture. The solution was stirred at room temperature for 6 h and quenched with methanol. The solvent was evaporated under reduced pressure. The residue was dissolved in toluene (3 × 10 mL) and concentrated to dryness. Then, the resulting crude was dissolved in dichloromethane (100 mL) and the organic phase was washed with 5% aqueous NaHCO3 (50 mL) and with saturated aqueous NaCl (50 mL). The solvent was evaporated and the residue was purified by flash chromatography on silica gel. The column was packed using a 1% triethylamine (TEA) solution in DCM. Compounds **2**, **9a** and **9c** were eluted with a gradient of MeOH from 0% to 4% in DCM and compound **9b** was eluted with a gradient of MeOH from 0% to 10% in DCM.

#### 3.4.1. *N-[(1R,2R)-1-[[bis(4-Methoxyphenyl)phenylmethoxy]methyl]-2-hydroxypropyl]-5-(dithiolan-3-yl)pentanamide* (**2**)

The compound was obtained as a mixture of two diastereoisomers. Yield: 295 mg (62%). IR (film): 3176, 2927, 1646, 1508, 1249, 1175, 1033 cm^−1^. ^1^H-NMR (400 MHz, CDCl_3_): δ_H_ = 7.67 (tt, *J* = 7.56 Hz, 7.7 Hz; 1H, C*H_arom_*), 7.36 (m, 2H, (C*H_arom_*), 7.27 (m, 8H, C*H_arom_*), 6.84 (d, *J* = 8.8 Hz, 2H, C*H_arom_*), 6.7 (br, 1H, N*H*), 4.07 (m, 1H, OH-C*H*-CH_3_), 3.92 (m, 1H, CH-C*H*-OH), 3.79 (s, 6H, 2 OC*H_3_*), 3.72 (m, 1H, C*H*-CH-OH), 3.56 (m, 1H, N-C*H*-CH_2_), 3.41 (dd, *J* = 9.6 Hz, 4.2 Hz, 1H, S-C*H*-CH_2_-), 3.30 (dd, *J* = 9.4 Hz, 4.2 Hz, 1H, S-C*H*-CH_2_-), 3.17 (m, 2H, S-C*H_2_*-CH_2_-), 3.00 (br, 1H, O*H*), 2.45 (m, 1H, S-C*H*-CH_2_-), 2.23 (t, *J* = 7.4 Hz; 2H, CO-C*H_2_*), 1.90 (m,2H, -C*H_2_*-CH_2_-), 1.69 (m, 4H, alkyl residue), 1.49 (m, 2H, alkyl residue), 1.13 (d, *J* = 6.4 Hz, 3H, CH-C*H_3_*) ppm.^13^C-NMR (125 MHz, CDCl_3_): δ_C_ = 173.25 (*C*O), 158.84 (*C_arom_*), 150.06 (*C_arom_*), 144.51 (*C_arom_*), 136.14 (*C_arom_*), 135.73 (*C_arom_*), 135.66 (*C_arom_*), 135.53 (*C*H*_arom_*), 130.01 (*C*H*_arom_*), 128.20 (*C*H*_arom_*), 128.18 (*C*H*_arom_*), 127.23 (*C*H*_arom_*), 123.92 (*C*H*_arom_*), 113.57 (*C*H*_arom_*), 87.03 (*C*-C_arom_), 68.94 (*C*H-O-), 65.55 (*C*H_2_-O), 56.57 (N-*C*H-CH_2_-), 55.63 (O*C*H_3_), 55.49 (O*C*H_3_), 53.54 (CO*C*H_2_), 40.43 (*C*H_2_-S), 38.64 (CH_2_-*C*H-S), 36.73, 34.93, 29.16, 25.76 (alkyl chain), 20.18 (*C*H_3_-CH) ppm. HRMS (ESI+): *m/z* calcd for C_66_H_82_N_2_NaO_10_S_4_ ([2M + Na]^+^) 1213.4745, found 1213.4745.

#### 3.4.2. *N-[(1R,2R)-1-[[bis(4-Methoxyphenyl)phenylmethoxy]methyl]-2-hydroxypropyl]-N'-[3-[2-[2-[3-[5-(dithiolan-3-yl)pentanoylamino]propoxy]ethoxy]ethoxy]propyl]butanediamide* (**9a**)

The compound was obtained as a mixture of two diastereoisomers. Yield: 467 mg (65%). IR (film): 2929, 2662, 1653, 1608, 1545, 1508, 1463, 1393, 1249, 1178, 1131, 1095, 1033, 753 cm^−1^. ^1^H-NMR (400 MHz, CDCl_3_): δ_H_ = 7.39 (d, *J* = 7.3 Hz, 2H, C*H_arom_*), 7.29 (m, 6H, C*H_arom_*), 7.21 (d, *J* = 7.3 Hz, 1H, C*H_arom_*), 6.84 (d, *J* = 8.9 Hz, 4H, C*H_arom_*), 6.69 (br, 1H, N*H*), 6.64 (br, 1H, N*H*), 6.49 (br, 1H, N*H*), 4.07 (m, 1H, CH_3_-C*H*-OH), 3.91 (m, 3H, N-C*H*-C*H_2_*-OH), 3.79 (s, 6H, 2 OC*H_3_*), 3.64 (m, 8H, 2 O-C*H_2_*-C*H_2_*-O), 3.59–3.54(m, 4H, O-C*H_2_*-C*H_2_*-O), 3.34 (m, 4H, 2 NH-C*H_2_*-), 3.09 (m, 4H, S-C*H_2_*-CH_2_ and C*H_2_*-NHCO), 2.52 (m, 5H, S-C*H*-CH_2_- and NH-C*H_2_*-C*H_2_*-CH_2_-O), 2.14 (t, *J* = 7.2 Hz, 2H, CO-C*H_2_*), 1.85–1.49 (m, 8H, alkyl residues), 1.12 (2d, *J* = 6.4 Hz, 6H, 2 C*H_3_*-CH) ppm. ^13^C-NMR (125 MHz, CDCl_3_): δ_C_ = 172.83 (*C*O), 172.74 (*C*O), 172.06 (*C*O), 158.55 (*C_arom_*), 145.36 (*C_arom_*), 135.87 (*C_arom_*), 135.79 (*C_arom_*), 130.06 (*C*H*_arom_*), 129.89 (*C*_arom_), 127.82 (*C*H*_arom_*), 127.79 (*C*H*_arom_*), 126.43 (*C*H*_arom_*), 113.44 (*C*H*_arom_*), 86.54 (*C*-O), 70.57 (*C*H-O-), 70.49 (*C*H-O-), 70.05 (*C*H-O-), 69.98 (*C*H-O-), 69.88 (*C*H-O-), 68.26 (*C*H-O-), 64.8 (*C*H-O-), 56.44 (O*C*H_3_), 55.23 (O*C*H_3_), 54.06 (*C*H-N), 53.64 (*C*H-N), 41.93 (*C*H_2_-S), 38.46 (*C*H_2_-S), 37.83 (CH_2_-*C*H-S), 36.37(CO*C*H_2_), 34.68 (N-*C*H_2_), 31.95 (N-*C*H_2_), 31.72, 29.62, 29.08, 28.96, 28.86, 25.43, 19.82, 17.33 (alkyl residue), 17.31 (*C*H_3_CH) ppm. HRMS (ESI+): *m/z* calcd for C_26_H_50_N_3_O_8_S_2_ ([M + H-DMT]^+^) 596.3033, found 596.3032.

#### 3.4.3. *O^1^-(4, 4'-Dimetoxytriphenylmethyl)-N-[N'-{5-(1,2-dithiolan-3-yl)pentanoyl}glycylglycylglycyl]-l-threoninol* (**9b**)

The compound was obtained as a mixture of two diastereoisomers. Yield: 325 mg (53%). ^1^H-NMR (400 MHz, DMSO-*d*_6_): δ_H_ = 8.13 (t, *J* = 5.6 Hz, 1H, CH_2_-N*H*-CO), 8.07–8.04 (m, 2H, CH_2_-N*H*-CO), 7.50 (d, *J* = 7.6 Hz, CH-N*H*-CO), 7.37–7.17 (m, 9H, C*H_arom_*), 6.86 (d, *J* = 8.8 Hz, 4H, C*H_arom_*), 4.54 (d, *J* = 5.2 Hz, 1H, O*H*-CH), 3.95–3.90 (m, 1H, OH-C*H*-CH_3_), 3.89–3.82 (m, 1H, NH-C*H*-CH_2_), 3.78–3.69 (m, 12H, 2 OC*H_3_* and 3 NH-C*H_2_*-CO), 3.61–3.53 (m, 1H, S-C*H*-CH_2_), 3.18–3.02 (m, 3H, DMTO-C*H_(A)_*H_(B)_-CH and S-C*H_2_*-CH_2_), 2.86–2.82 (m, 1H, DMTO-CH_(A)_*H_(B)_*-CH), 2.41–2.34 (m, 1H, S-CH-C*H_(A)_*H_(B)_-CH_2_), 2.12 (t, *J* = 7.2 Hz, 2H, CH_2_-C*H_2_*-CO-NH), 1.88–1.79 (m, 1H, S-CH-CH_(A)_*H_(B)_*-CH_2_), 1.68–1.59 (m, 2H, alkyl chain), 1.54–1.46 (m, 2H, alkyl chain), 1.36–1.31 (m, 2H, alkyl chain), 0.94 (d, *J* = 6.0 Hz, 3H, C*H_3_*-CH) ppm. ^13^C-NMR (125 MHz, DMSO-d_6_): δ_C_ = 173.25 and 173.16 (NH-*C*O)^(*)^, 170.31, 170.29, 170.23 and 170.21 (NH-*C*O)^(*)^, 169.64, 169.62, 169.57 and 169.55 (NH-*C*O)^(*)^, 169.29, 169.23 and 169.21 (NH-*C*O)^(*)^, 158.65 (*C*_arom_), 145.76 (*C*_arom_), 136.49 (*C*_arom_), 136.45 (*C*_arom_), 130.37 (*C*H_arom_), 128.45 (*C*H_arom_), 128.39 (*C*H_arom_), 127.22 (*C*H_arom_), 113.80 (*C*H_arom_), 85.82 (*C*-O), 65.44 and 65.33 (CH_3_-*C*H)^(*)^, 63.39 (DMTO-*C*H_2_-CH), 56.79 (S-*C*H-CH_2_), 55.69 (O-*C*H_3_), 54.74, 54.72, 54.66 and 54.63 (NH-*C*H-CH)^(*)^, 46.24 (S-*C*H_2_-CH_2_), 42.77 and 42.75 (NH-*C*H_2_-CO)^(*)^, 42.66 and 42.63 (NH-*C*H_2_-CO)^(*)^, 42.47 and 42.45 (NH-*C*H_2_-CO)^(*)^, 38.77 (S-CH-*C*H_2_), 35.64 and 35.59 (CH_2_-*C*H_2_-CO-NH)^(*)^, 34.81 (*C*H_2_ alkyl chain), 29 01 (*C*H_2_ alkyl chain), 25.51 (*C*H_2_ alkyl chain), 20.87 and 20.81 (*C*H_3_-CH)^(*)^ ppm. HRMS (ESI+): *m/z* calcd for C_39_H_50_N_4_NaO_8_S_2_ ([M + Na]^+^) 789.2968, found 789.2960; *m/z* calcd for C_18_H_33_N_4_O_6_S_2_ ([M-DMT+H]^+^) 465.1842, found 465.1845; *m/z* calcd for C_18_H_32_N_4_NaO_6_S_2_ ([M-DMT + Na]^+^) 487.1661 found 487.1661. ^(*)^Multiple peaks observed due to rotamers of the amide bond.

#### 3.4.4. *N-[(1R,2R)-1-[[bis(4-Methoxyphenyl)phenylmethoxy]methyl]-2-hydroxypropyl]-8-[5-(dithiolan-3-yl)pentanoylamino]octanamide* (**9c**)

The compound was obtained as a mixture of two diastereoisomers. Yield: 277 mg (47%). IR (film): 3019, 2925, 1652, 1058, 1215 cm^−1^. ^1^H-NMR (400 MHz, CDCl_3_): δ_H_ = 7.67 (tt, *J* = 7.6 Hz, 1.8 Hz, 1H, C*H_arom_*), 7.39 (m, 2H, C*H_arom_*), 7.27 (m, 8H, C*H_arom_*), 6.83 (d, *J* = 8.2 Hz, 2H, C*H_arom_*), 6.1 (br, 1H, N*H*), 4.08 (m, 1H, OH-C*H*-CH_3_), 3.94 (m, 1H, CH-C*H*-OH), 3.78 (s, 6H, 2 OC*H_3_*), 3.56 (m, 1H, N-C*H*-CH_2_), 3.39 (dd, *J* = 9.5 Hz, 4.5 Hz, 1H, CH-C*H*-OH), 3.23 (m, 2H, S-C*H_2_*-CH_2_-), 3.10 (m, 2H, C*H_2_*-N), 2.42 (m, 1H, S-C*H*-CH_2_-), 2.21 (t, *J* = 7.5 Hz, 2H, CO-C*H_2_*), 2.14 (t, *J* = 7.5 Hz, 2H, COC*H_2_*), 1.89 (m, 2H, -C*H_2_*-CH_2_-), 1.64 (m, 6H, alkyl residue), 1.45 (m, 4H, alkyl residue), 1.32 (m, 6H, alkyl residues), 1.12 (d, *J* = 6.3 Hz, 3H, C*H_3_*-CH) ppm. ^13^C-NMR (125 MHz, CDCl_3_): δ_C_ = 173.63 (*C*O), 172.91 (*C*O), 158.82 (*C_arom_*), 149.93 (*C_arom_*), 144.66 (*C_arom_*), 136.24 (*C_arom_*), 135.84 (*C_arom_*), 135.66 (*C_arom_*), 130.21 (*C*H*_arom_*), 130.19 (*C*H*_arom_*), 128.20 (*C*H*_arom_*), 128.18 (*C*H*_arom_*), 127.21 (*C*H*_arom_*), 124.05 (*C*H*_arom_*), 113.57 (*C*H*_arom_*), 86.86 (*C*-C*_arom_*), 68.55 (*C*H-O-), 65.37 (*C*H_2_-O), 56.73 (N-*C*H-CH_2_-), 55.46 (O*C*H_3_), 53.63 (CO*C*H_2_), 40.47 (*C*H_2_-S), 39.65 (*C*H_2_-S), 38.63 (CH_2_-*C*H-S), 36.94 (CO*C*H_2_), 36.69 (N*C*H_2_), 34.83, 29.76, 29.20, 29.19, 29.08, 26.83, 25.88, 25.68 (alkyl residues), 20.25 (*C*H_3_CH) ppm. HRMS (ESI+): *m/z* calcd for C_41_H_56_N_2_NaO_6_S_2_ ([M + Na]^+^) 759.3472, found 759.3475; *m/z* calcd for C_82_H_112_N_4_NaO_12_S_4_ ([2M + Na]^+^) 1495.7052, found 1495.7032.

### 3.5. Functionalization of CPG Solid Supports **4** and **11a**–**11c**

The DMT derivatives (**2**, and **9a**–**9c**) were incorporated on a long-chain alkylamine-controlled pore glass support (LCAA-CPG) as described [56] using their corresponding hemisuccinate derivatives as follows. The DMT derivative (0.05 mmol) was dried by evaporation with anhydrous ACN under reduced pressure. The residue was dissolved in anhydrous pyridine (5 mL) under argon. Succinic anhydride (26 mg, 0.26 mmol) and DMAP (2.5 mg, 0.02 mmol) were dissolved in 1 mL of pyridine and added to the solution. The solution was stirred overnight at room temperature. The solvent was removed under reduced pressure and the residue was dissolved in toluene (3 × 10 mL) and concentrated to dryness. The resulting material was dissolved in dichloromethane (20 mL). The solution was washed with 0.1 M sodium monophosphate (15 mL) and saturated aqueous NaCl (15 mL). The organic layer was dried over anhydrous MgSO_4_, filtered and evaporated to dryness. Yellowish oils were obtained in each case. The resulting hemisuccinates **3** and derivatives**10a**–**10c** were used in the next step without further purification.

Amino-LCAA-CPG (CPG New Jersey, 73 μmol amino/g, 200 mg) was placed into a polypropylene syringe fitted with a polypropylene disc and washed sequentially with DMF, MeOH, THF, DCM and ACN (2 × 5 mL). Then, a solution of the appropriate hemisuccinate (25 µmol) and triethylamine (25 µL, 175 µmol) in anhydrous ACN (500 µL) was prepared. The solution was added to the support and then a solution of 2-(1*H*-benzotriazole-1-yl)-1,1,3,3-tetramethyluronium tetrafluoroborate (TBTU) (56 mg, 175 µmol) in anhydrous ACN (300 µL) was added. The mixture was left to react for 1 h. The support was washed with DMF, MeOH, DCM and ACN (2 × 5 mL). The coupling procedure was repeated once more if needed and the functionality of the resin was determined by DMT quantification. The functionalization obtained in each case were *f* = 19.0 μmol/g for solid support **4**, 18.5 μmol/g for **11a**, 25.3 μmol/g for **11b** and 15.1 μmol/g for **11c**. Finally, each solid support was treated with a mixture of Ac2O/DMF (1:1, 500 μL) to cap free amino groups.

### 3.6. N-[(1R,2R)-1-{[bis(4-Methoxyphenyl)phenylmethoxy]methyl}-2-[2-cyanoethoxydiisopro-pylamino)phosphanyl]oxypropyl]-4-(dithiolan-3-yl)butanamide (**12**)

Compound **2** (100 mg, 0.13 mmol) was dried by evaporation of anhydrous ACN (3 × 10 mL) under reduced pressure and dried in a desiccator for one hour. Then, compound **2** was dissolved in anhydrous DCM (2 mL) and DIEA (69 µL, 0.39 mmol) was added dropwise at room temperature. The solution was cooled at 0 °C and 2-cyanoethoxy-*N,N*-diisopropylaminochlorophosphine (46 µL, 0.19 mmol) was added dropwise with a syringe. Reaction was stirred 5 min at 0 °C and 1 h at room temperature. After this time the reaction was complete as judged by TLC. Then, DCM was added (10 mL) and organic layer was washed with a 5% NaHCO_3_ aqueous solution (1 × 5 mL) and brine (1 × 5 mL). Finally, solvent was evaporated and the crude was purified by flash chromatography (Hex/AcOEt 20% to 50%) to give compound **12** (80 mg, 77% yield). The compound was obtained as a mixture of diastereoisomers. IR (film): 2966, 2929, 2360, 2342, 1673, 1607, 1508, 1249, 1177, 1034 cm^−1^. ^1^H-NMR (400 MHz, CDCl_3_): δ_H_ = 7.38 (m, 2H), 7.26 (m, 7H), 6.29 (m, 4H), 5.80 (broad s, N*H*), 4.30 (m, 1H), 4.23 (m, 5H), 3.75 (s, 6H, 2 OC*H*_3_), 3.47 (m, 4H), 3.14 (m, 2H), 2.70 (m, 2H), 2.36 (m, 1H), 2.17 (m, 2H), 1.90 (m, 2H), 1.63 (m, 4H), 1.30–1.15 (m, 15H) ppm; ^13^C-NMR (125 MHz, CDCl_3_) δ_C_ = 173.3 (*C*O), 158.4 (*C_arom_*), 144.8 (*C_arom_*), 135.9 (*C_arom_*), 130.1 (*C_arom_*), 130.0 (*C_arom_*), 129.9 (*C_arom_*), 128.2 (*CH_arom_*), 128.0 (*CH_arom_*), 127.7 (*CH_arom_*), 126.7 (*CH_arom_*), 113.0 (*C*N), 86.0 (*C*-C_arom_), 68.7 (*C*H-O), 65.7 (*C*H_2_-O), 65.3 (P-O-*C*H_2_), 58.3 (N-*C*H-CH_2_), 58.1 (N-*C*H-CH_2_), 55.2 (O*C*H_3_), 53.3 (CO*C*H_2_), 43.0 (*C*H_2_-S), 40.2(*C*H-N), 38.4 (CH_2_-*C*H-S), 36.5, 34.6, 29.6, 24.7 (alkyl chain), 24.2 (*C*H_3_-CH), 22.6 (*C*H_3_-CH), 19.6 (*C*H_2_-CN) ppm; ^31^P-NMR (163 MHz, CDCl_3_) δ_P_ = 148.0 ppm; HRMS (ESI+): *m/z* calcd for C_42_H_59_N_3_O_6_PS_2_ ([M+H]^+^) 796.3583, found 796.3577.

### 3.7. Synthesis of the Model Oligonucleotides (T_12_)TA_I, and (T_12_)TA_III(**a**)–(**c**)

The TA terminated dodecathymidine oligonucleotides **(T_12_)TA_I**, and **(T_12_)TA_III(a)-(c)** (5'-TTT TTT TTT TTT-**X-**3', X being a TA modification) were synthesized on a DNA/RNA synthesizer (*Applied Biosystems*) on a 0.2 µmol scale using the corresponding modified CPG solid supports. The solid support **4** was used to synthesize the oligonucleotide **(T_12_)TA_I** and the corresponding solid supports **11(a)**–**(c)** for oligonucleotides **(T_12_)TA_III(a)**–**(c)**. The last DMT group was removed at the end of the synthesis. The resulting oligonucleotides were deprotected and cleaved from the solid supports with aqueous concentrated ammonia using three different conditions: (i) at room temperature for 4 h; (ii) at 55 °C for 1 h and (iii) at 55 °C overnight. The mixtures were filtered and ammonia solutions were concentrated to dryness. The resulting products were analyzed by HPLC. Solvent A: 5% ACN in 100 mM triethylammonium acetate (TEAA) (pH = 7) and solvent B: 70% ACN in 100 mM TEAA (pH = 7). Column: XBridge^TM^ OST C_18_ column; (4.6 × 50 mm, 2.5 µm). Flow rate: 1 mL/min. Conditions: 10 min of linear gradient from 5% to 35% of B. The isolated products were analyzed by mass spectrometry (MALDI-TOF) (Supplementary Information, Table S1).

### 3.8. Synthesis of 3'-TA-Modified Oligonucleotides and the 5'-Fluorescently Labeled 3'-TA-Modified Oligonucleotide Probes

The thioctic acid terminated 21mer oligonucleotides **TA_I**, **TA_II** and **TA_III(a)-(c)** (5'-CGG AGG TAC ATT CGA CTT GA-**X-**3', where X was a TA residue) were synthesized a 1 µmol scale using the corresponding modified CPG solid supports. Oligonucleotide **TA_II** was synthesized using the solid support **4** once the commercially available spacer 18-O-dimethoxytritylhexaethyleneglycol, 1-[(2-cyanoethyl)-(*N*,*N*-diisopropyl)] phosphoramidite was incorporated. Once the synthesis of **TA_I**, **TA_II** and **TA_III(a)**–**(c)** was completed, each solid support was divided in two. The last DMT group was removed at the end of the synthesis. One half was deprotected and the other half was used for the synthesis of the corresponding fluorescently labeled oligonucleotides **(F)TA_I**, **(F)TA_II** and **(F)TA_III(a)**–**(c)**. Coupling yields were >97%. **TA_I**, **TA_II** and their corresponding fluorescently labeled versions were treated with aqueous concentrated ammonia at 55 °C for 12 h to cleave the products from the supports and remove the Bz and ^i^Bu groups. The **TA_III** series were treated with aqueous concentrated ammonia at room temperature for 4 h to cleave the products from the supports and remove the Bz and dmf groups. The mixtures were filtered and ammonia solutions were concentrated to dryness. The resulting oligonucleotides were desalted and purified by HPLC using the same solvents described above. Column: XBridge^TM^ OST C_18_ semipreparative column (10 × 50 mm, 2.5 µm). Flow rate: 3 mL/min. Conditions: 10 min of linear gradient from 0% to 30% of B for non fluorescently labeled oligonucleotides and 10 min. of linear gradient from 6% to 40% for the corresponding fluorescently labeled oligonucleotides. The pure fractions were combined and evaporated to dryness. The resulting oligomers were analyzed by mass spectrometry, UV/Vis spectroscopy and HPLC as well using the conditions and gradients described above but using an analytic column (XBridge^TM^ OST C_18_ (4.6 × 50 mm, 2.5 μm). Flow rate: 1 mL/min), (Table S3, SI). The yields obtained ranged from 33% to 49%.

### 3.9. Synthesis of a 5'-TA-Modified Oligonucleotide

The thioctic acid terminated 21mer oligonucleotide **5'TA_I**, (5'-**X**-CGG AGG TAC ATT CGA CTT GA-3', where X was a TA residue) was synthesized on a 0.2 µmol scale employing LV200^®^ polystyrene supports. Phosphoramidite **12** was used to incorporate the TA derivative. The DMT determination showed that the efficiency of coupling of the phosphoramidite was >90%. The last DMT group was removed at the end of the synthesis. The solid support was treated with aqueous concentrated ammonia at 55 °C for 12 h. and oligonucleotide **5'TA_I** purification by HPLC was using the same protocol as described above. (Table S3, SI). The purified oligonucleotide was obtained in 51% yield.

### 3.10. Synthesis of 3'-Alkylthiolated-Modified Oligonucleotides and the 5'-Fluorescently Labeled 3'-Alkylthiolated Modified Oligonucleotide Probes

The 3'-disulfide-modified oligonucleotide **ALK_DS** (5'-CGG AGG TAC ATT CGA CTT GA-**Y-**3') (Y stands for 3'- linear alkyldisulfide or alkylthiol modifications) was synthesized on a 1 µmol scale using the 3'-Thiol modifier C3 S-S CPG following the procedure described previously. The last DMT group was removed at the end of the synthesis. As well, once the synthesis was completed, half of the solid support was used to incorporate the fluorescent dye at the 5'-termini to obtain the fluorescently labeled **(F)ALK_DS** analogue. Each solid support was treated with aqueous concentrated ammonia at 55 °C for 12 h. Work-up was similar as described above. The oligonucleotides were analyzed by HPLC using the conditions described above and characterized by mass spectrometry (MALDI-TOF) and UV/Vis spectroscopy (Table S3, SI) and used without further purification. The yields obtained were 74% for **ALK_DS** and 72% for **(F)ALK_DS**. The 3'-thiol-modified oligonucleotide **ALK_SH** and its fluorescently labeled version **(F)ALK_SH** were obtained from the corresponding disulfide oligonucleotides **ALK_DS** and **(F)ALK_DS** respectively by thiol deprotection [44,45]. The experimental procedure is as follows: disulfide oligonucleotides (5 OD_260_) were dissolved in 300 µL of an aqueous solution 0.1 M TEAA (pH = 7). Then, 34 L of an aqueous solution 0.1 M of tris(2-carboxyethyl)phosphine hydrochloride (TCEP·HCl) were added to the solution and allowed to react at 55 °C overnight. Under these conditions, the disulfide group was completely removed and the deprotected thiol oligonucleotides (**ALK_SH** and **(F)ALK_SH**, respectively) were purified with Sephadex G-25 (NAP-5 column) and used in the next step without further purification.

### 3.11. Functionalization of Gold Nanoparticles

Colloidal gold nanoparticles (9.7 nm diameter) were functionalized with the TA-modified oligonucleotides (**TA_I**, **TA_II**, **TA_III(a)**–**(c)**), with 3'-alkylthiolated-modified oligonucleotides (**ALK_DS** and **ALK_SH**) and with their respectively fluorescently labeled versions as well. To prepare the conjugates, 5 nmol of desalted oligonucleotide were dissolved in 1 mL of MilliQ water and were added to a 1 mL of the gold nanoparticles solution. After 24 h, the solution was brought to a final concentration of 10 mM sodium phosphate (pH = 7.2). The mixture was then allowed to equilibrate for 30 min before bringing the concentration to 0.15 M NaCl over a 7 h 30 min period in a stepwise manner (0.05 M NaCl increments each 2 h 30 min). The solutions were sonicated for 10 s. before each addition to keep the particles dispersed during the salting procedure. The salting process was followed by an incubation period of 48 h at room temperature. Finally, to remove all unbound oligonucleotides, the solution was centrifuged at 13,200 rpm (16,100 ×*g*) for 30 min. This procedure was repeated three times. As we wanted to compare stabilities of the different DNA-AuNP conjugates, we dispersed the reddish residue in 500 µL of 0.15 M NaCl, 10 mM sodium phosphate (pH = 7.2), 0.01% NaN_3_ solution. Then, each solution was analyzed by UV-visible absorption spectroscopy and the concentration of gold nanoparticle conjugates was adjusted to 6.9–7.1 nM by adding the required volume of buffer. The conjugates obtained with the fluorescent oligonucleotides were directly resuspended in 1 mL of a 0.15 M NaCl, 10 mM sodium phosphate (pH = 7.2), 0.01% NaN_3_ solution. The extinction coefficient used was the same as that used for unmodified nanoparticles (ε_520_ = 7.6 × 10^7^ M^−1^·cm^−1^, data provided by the manufacturer). Each solution was then stored in the fridge (4 °C) prior to use.

### 3.12. Stability Tests of Oligonucleotide-Gold Nanoparticle Conjugates

The stability of the different TA-terminated oligonucleotides **TA_I**, **TA_II**, **TA_III(a)**–**(c)** gold nanoparticle conjugates was assessed by treating the AuNP-conjugates with a DTT solution based on protocols described in the literature [22,37]. The colloidal stability of AuNP conjugates obtained with disulfide and thiol modified oligonucleotides (**ALK_DS** and **ALK_SH** respectively) were studied as well for comparison purposes. Then, to a 500 µL of each solution were added 12.5 µL of a 4 M DTT solution (the final concentration of DTT was 100 mM). Prior to the addition of DTT, the solutions were allowed to equilibrate at 40 °C and were sonicated for 20 s to keep the particles dispersed. To give an indication of the durability of the oligonucleotide conjugates, absorbance at 675 nm was monitored against time at 40 °C. For AuNPs conjugated with TA-terminated oligonucleotides absorbance was recorded at 1 min intervals and for AuNPs conjugated with alkylthiolated oligonucleotides absorbance was recorded at 1 s intervals. The measures were performed with 0.5 cm quartz cuvettes. The cells used had a ground hole at the top to adapt a PTFE stopper to provide a more suitable seal to reduce evaporation of the sample. Then, the half-life (time to reach half the value for complete aggregation) was calculated for each conjugate by fitting the data obtained to a Boltzmann equation using the Origin 8.0 software.

### 3.13. Oligonucleotide Loading Quantification on Gold Nanoparticles

The number of oligonucleotides loaded onto gold nanoparticles was analyzed following procedures previously described [37,47]. First, the concentration of nanoparticles and the concentration of fluorescent DNA in each sample were measured followed by the displacement of fluorescent oligonucleotides from the nanoparticle surface using a DTT solution. To this end, 200 µL of the gold nanoparticle conjugate solution were treated with 200 µL of a 1 M DTT solution in 0.18 M sodium phosphate buffer, (pH = 8) at room temperature for 2 h. The sample was centrifuged to remove the sediment from the supernatant and then the fluorescence spectra were recorded (conditions: λ_Ex_ = 495 nm and λ_Em_ = 510 − 550 nm). For each oligonucleotide two different oligonucleotide-AuNP conjugates were prepared and two different samples were prepared for each conjugate. Fluorescent measurements were done in duplicate. Standard curves were prepared with known concentrations of the fluorescently labeled TA-terminated oligonucleotide **(F)TA_I** using the same buffer, salt and DTT concentrations. The average number of oligonucleotides per particle was obtained by dividing the measured oligonucleotide molar concentration by the original oligonucleotide-AuNP conjugate concentration. The average area that each oligonucleotide occupies on the nanoparticle surface (footprint) was calculated by dividing the calculated surface area (nm^2^) by the average number of oligonucleotides per particle.

### 3.14. Stability of Thioctic Derivatives to Cathepsin

Cathepsin B was dissolved in this buffer: 50 mM NaCl, 10 mM sodium phosphate pH = 6, 1 mM EDTA and 30% glycerol and stored at −20 °C until needed. In order to find the optimal conditions for the enzymatic reaction, different assays were carried out at different pH, reaction times and also changing the proportions of the agents in the reaction. The optimal conditions were as follow: 13.8 µL (0.02 unt/µL) of Cathepsin B were added to 200 µL of a solution containing 0.25 OD’s of different modified oligonucleotides (**TA_I**, **TA_II**, **TA_III(a)**–**(c)**) and 5 mM of reduced glutathione. The reaction buffer was 100 mM NaCl, 40 mM sodium acetate, 1 mM EDTA and 1% triton-100x, pH = 5. The reaction mixture was left at 37 °C, with soft stirring during 12, 24, 48, 72 and 168 h respectively. The reaction was quenched by heating the samples at 70 °C during 20 min. The resulting products were analyzed by HPLC. Solvent A: 5% ACN in 100 mM TEAA (pH = 7) and solvent B: 70% ACN in 100 mM TEAA (pH = 7). Column: Nucleosil 120‒10 C18; (250 × 4 mm). Flow rate: 1 mL/min. Conditions: 20 min of linear gradient from 10% to 40% of B. The isolated products were analyzed by mass spectrometry.

## 4. Conclusions

l-Threoninol has been used as a building block to introduce in a covalent way to a DNA sequence four different ligands in length containing a TA moiety. All TA-appended oligonucleotide conjugates were efficiently loaded on gold nanoparticles. Stability experiments confirmed that all DNA-gold nanoparticle conjugates with TA modified at 3'-termini showed better stabilities through thiol exchange in the presence of DTT than those conjugates containing acyclic disulfides such as the commercially available 3'-thiol modifiers. On the other hand, we observed that oligonucleotides loaded onto gold nanoparticles containing a TA-alkyl ligand (**AuNP-TA_III(c)**) or a TA-oligo(ethylene glycol) ligand (**AuNP-TA_III(a)**) showed a remarkable increase in probe stability probably due to the disposal of the spacers, covering and protecting the gold nanoparticle surface. This increase in the stability of the system may be also due to the position of the amide group, which may induce favorable interactions between the neighboring alkyl or oligo(ethylene glycol) chains. Finally, the thioctic derivative carrying the triglycine linker is shown to be the most sensitive to cathepsin B. These preliminary results may open the door to an interesting application for these oligonucleotide derivatives in cellular delivery applications. 

## Supplementary Materials

Supplementary materials can be accessed at: http://www.mdpi.com/1420-3049/19/7/10495/s1.
