# Supplementary material for: Thioctic Acid Derivatives as Building Blocks to Incorporate DNA Oligonucleotides onto Gold Nanoparticles
Source: Molecules. 2014 Jul 18;19(7):10495–523. doi: 10.3390/molecules190710495 (PMC6271687; doi:10.3390/molecules190710495)
Supplement: Supplementary file 1 [file molecules-19-10495-s001.pdf]

Electronic Supporting Information (ESI)

1. HPLC and Mass Spectrometry Analysis (MALDI-TOF) of (T_{12})TA_I and (T_{12})TA_III(a)–(c) Oligonucleotides

Figure S1. HPLC profiles of the mixtures obtained after different cleavage conditions of oligonucleotides **(A)** **(T_{12})TA_I**; **(B)** **(T_{12})TA_III(a)**; **(C)** **(T_{12})TA_III(b)** and **(D)** **(T_{12})TA_III(c)**. Black lines correspond to the samples treated for 4 h at r.t., blue lines to the samples treated for 1 h at 55 ℃ and green lines to the samples treated o.n. at 55 ℃. ▼ stands for TA-terminated oligonucleotides, ■ stands for a β-elimination side product and ♦ stands for and amide bond-cleavage side product.

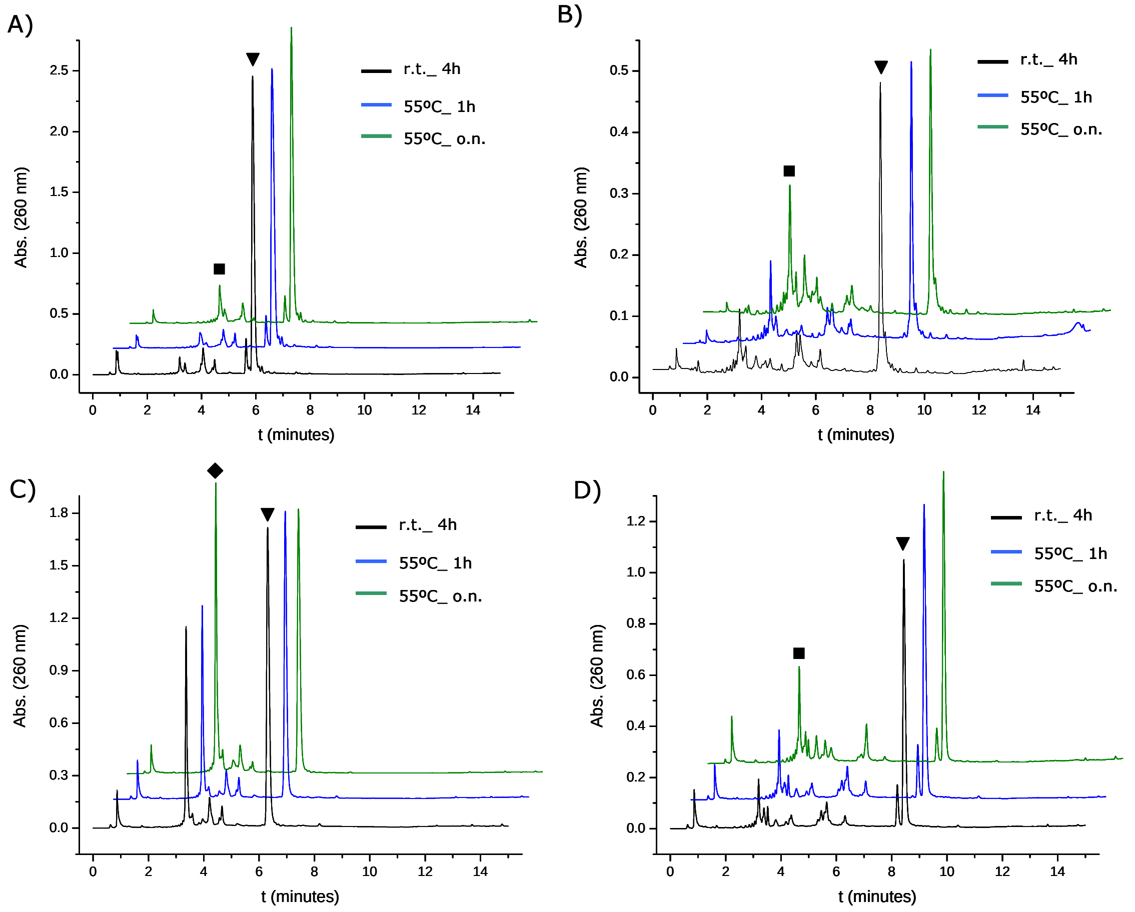

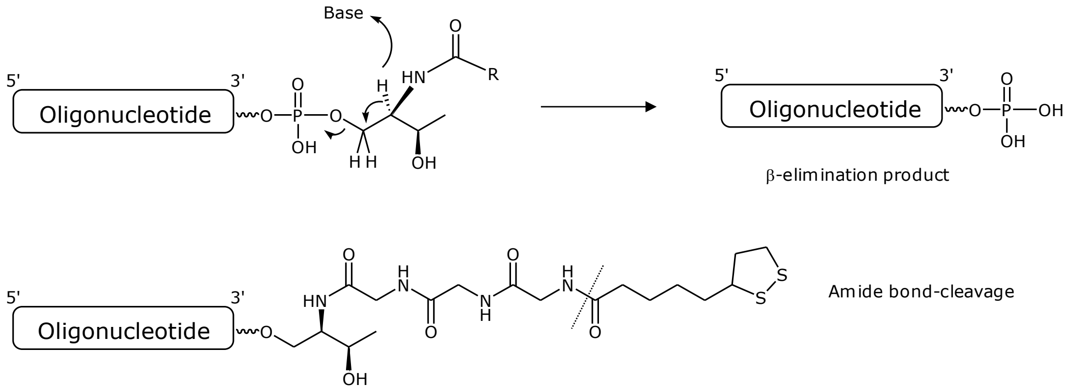

Table S1. Mass spectrometry analysis (MALDI-TOF) of the main products and the main side products resulting from the cleavage of **(T_{12})TA_I** and **(T_{12})TA_III(a)–(c)**.

	t_R (min)	Mass Found	Mass Calcdt.	Compound
**(T_{12})TA_I**	5.9	3943.8	3943.6	(T_{12})TA_I
	4.1	3960.0	3959.6 (+1 O)	oxidation product
		3975.9	3975.6 (+2 O)	
	3.4	4010.3	4009.6 (+3 O)	oxidation products
		4025.9	4025.6 (+4 O)	
	3.2	3668.5	3668.4	β-elimn. product
**(T_{12})TA_III(a)**	8.4	4246.5	4246.0	(T_{12})TA_III(a)
	5.4	4262.4	4262.0 (+1 O)	oxidation product
	4.2	4278.5	4278.0 (+2 O)	oxidation product
		4312.9	4312.0 (+4 O)	
	3.7	4329.1	4328.0 (+5 O)	oxidation products
		4345.2	4344.0 (+6 O)	
	3.2	3667.5	3668.4	β-elimn. product
**(T_{12})TA_III(b)**	6.2	4114.8	4114.8	(T_{12})TA_III(b)
	4.1	4131.3	4130.8 (+1 O)	oxidation product
	3.2	3926.4	3926.6	amide bond cleavage
**(T_{12})TA_III(c)**	8.5	4084.9	4084.8	(T_{12})TA_III(c)
	5.7	4102.3	4100.8 (+1 O)	oxidation product
	4.1	4117.8	4116.8 (+2 O)	oxidation product
		4151.8	4150.8 (+4 O)	
	3.9	4168.1	4166.8 (+5 O)	oxidation products
		4184.9	4182.8 (+6 O)	
	3.2	3668.5	3668.4	β-elimn. product

Table S2. Expected conjugates and by-products resulting from the different cleavage conditions of **(T_{12})TA_I** and **(T_{12})TA_III(a)–(c)**. The % of each compound was determined by the HPLC analysis.

	Treatment	Oligonucleotide (%)	β-elim. (%)	Oxidation (%)	Amide Bond Cleavage (%)
**(T_{12})TA_I**	4 h r.t.	86	3	11	
	1 h 55 ℃	87	4	9	
	o.n. 55 ℃	83	9	8	
**(T_{12})TA_III(a)**	4 h r.t.	75	10	15	
	1 h 55 ℃	71	13	16	
	o.n. 55 ℃	59	18	23	
**(T_{12})TA_III(b)**	4 h r.t.	67	---	8	25
	1 h 55 ℃	66	---	7	27
	o.n. 55 ℃	49	---	8	43
**(T_{12})TA_III(c)**	4 h r.t.	76	8	16	
	1 h 55 ℃	73	10	17	
	o.n. 55 ℃	64	13	23	

Figure S2. MALDI-TOFF mass spectrometry of the main products and the OH main side products resulting from the cleavage of **(T_{12})TA_I** and **(T_{12})TA_III(a)–(c)**.

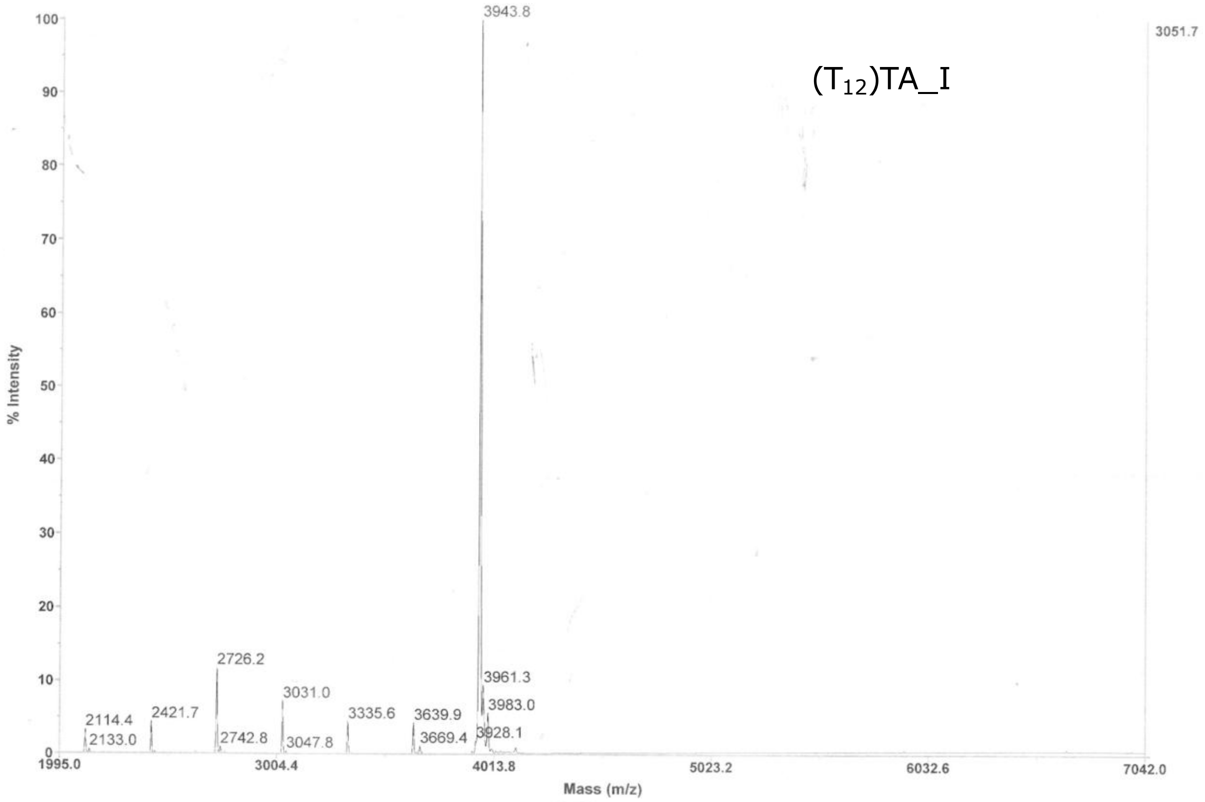

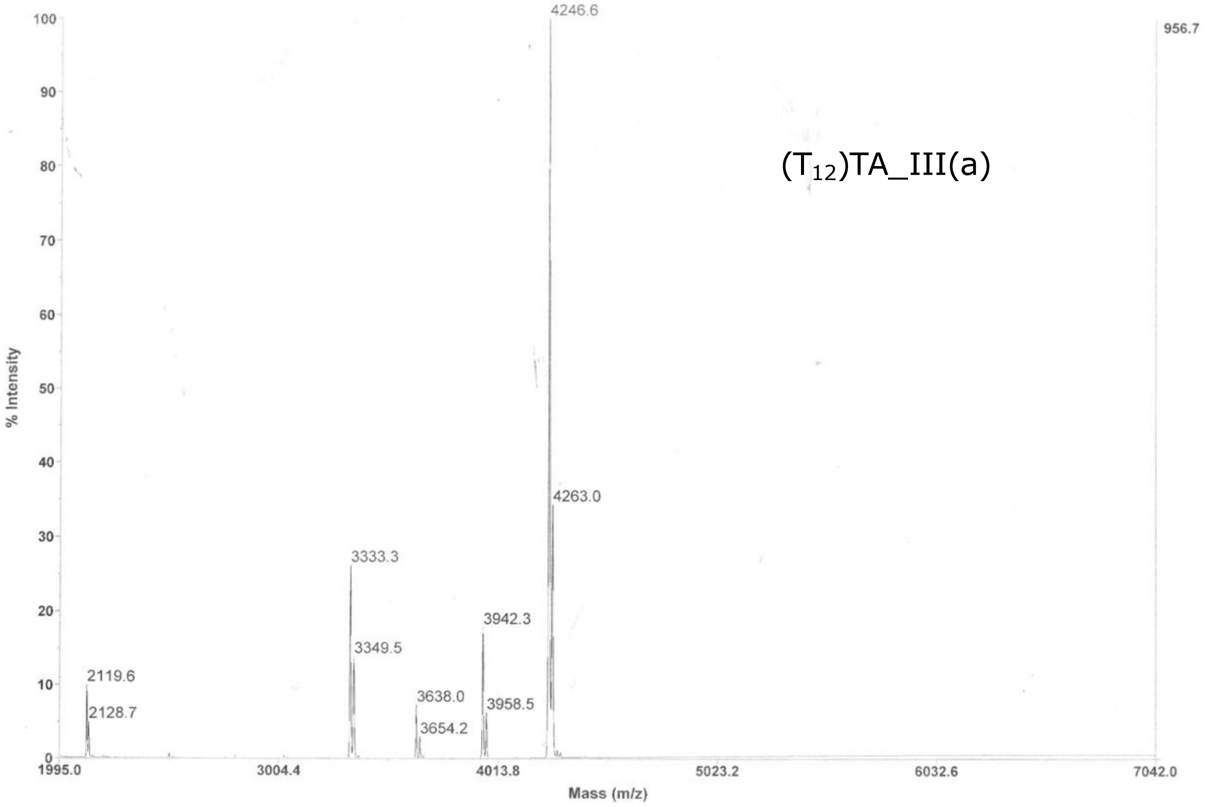

Figure S2. *Cont.*

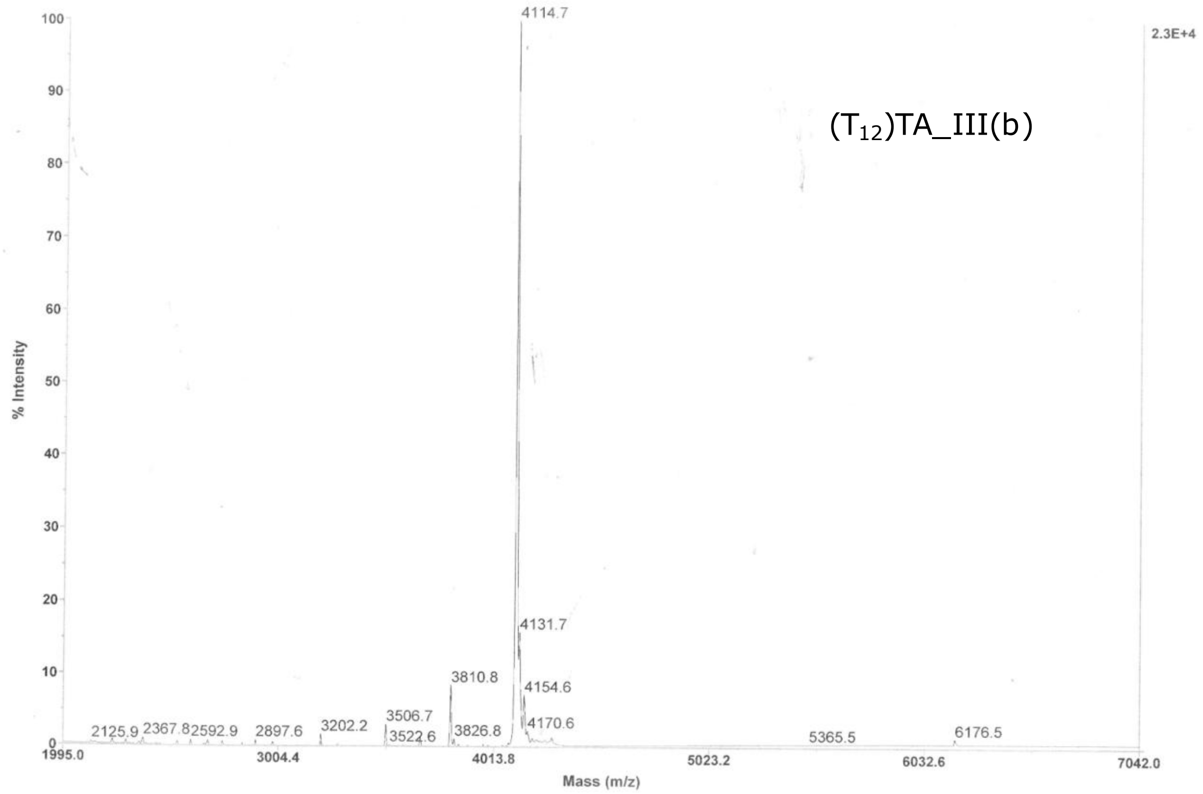

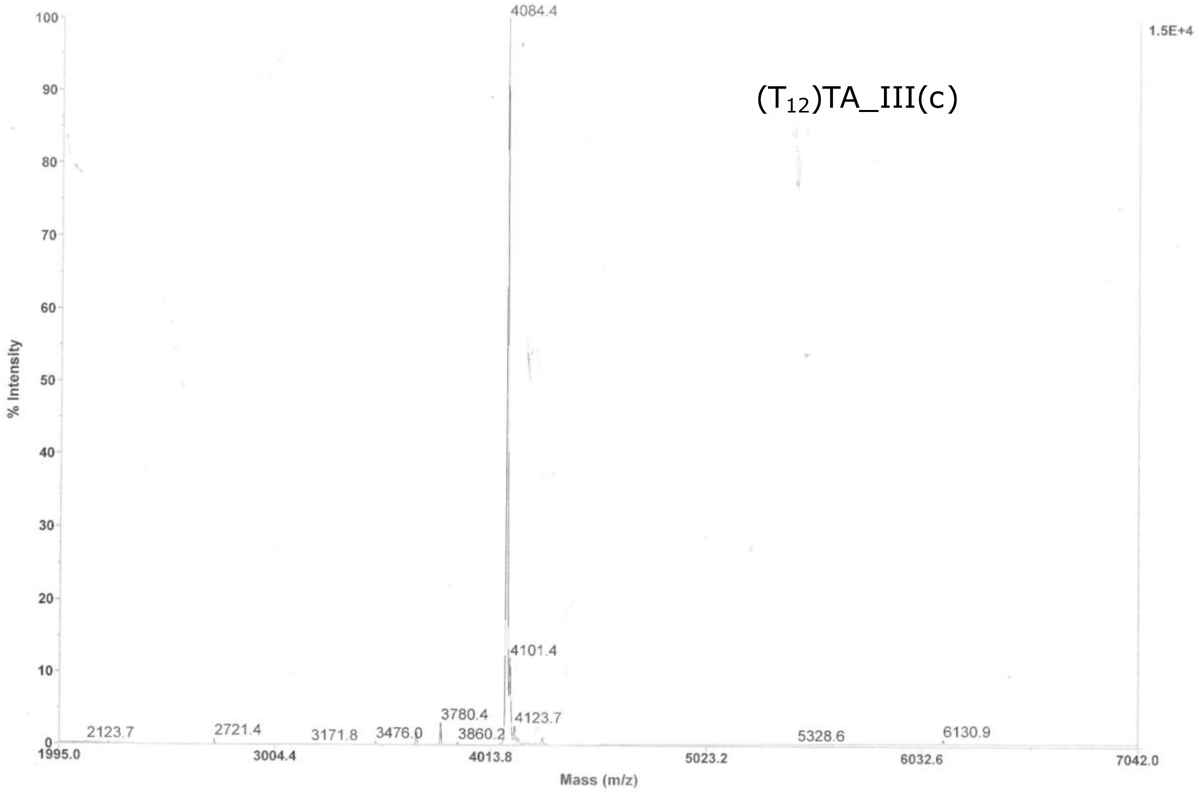

Figure S2. *Cont.*

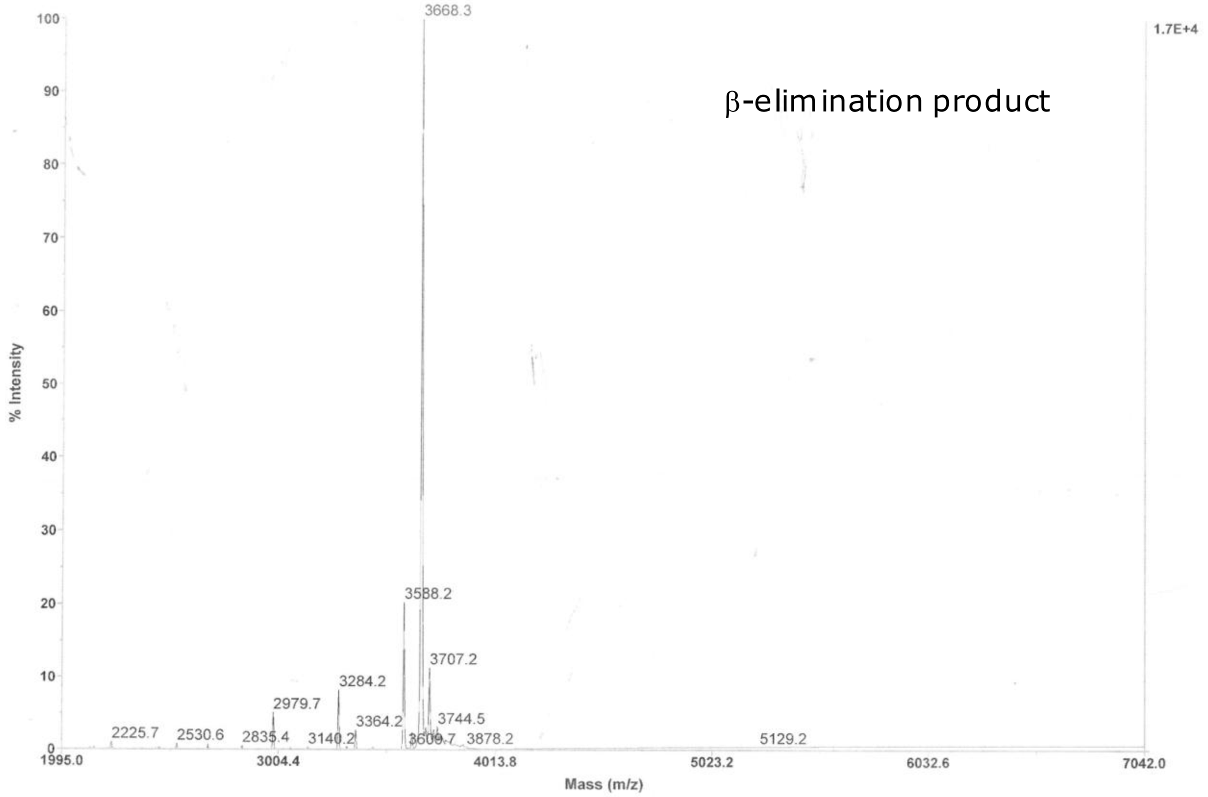

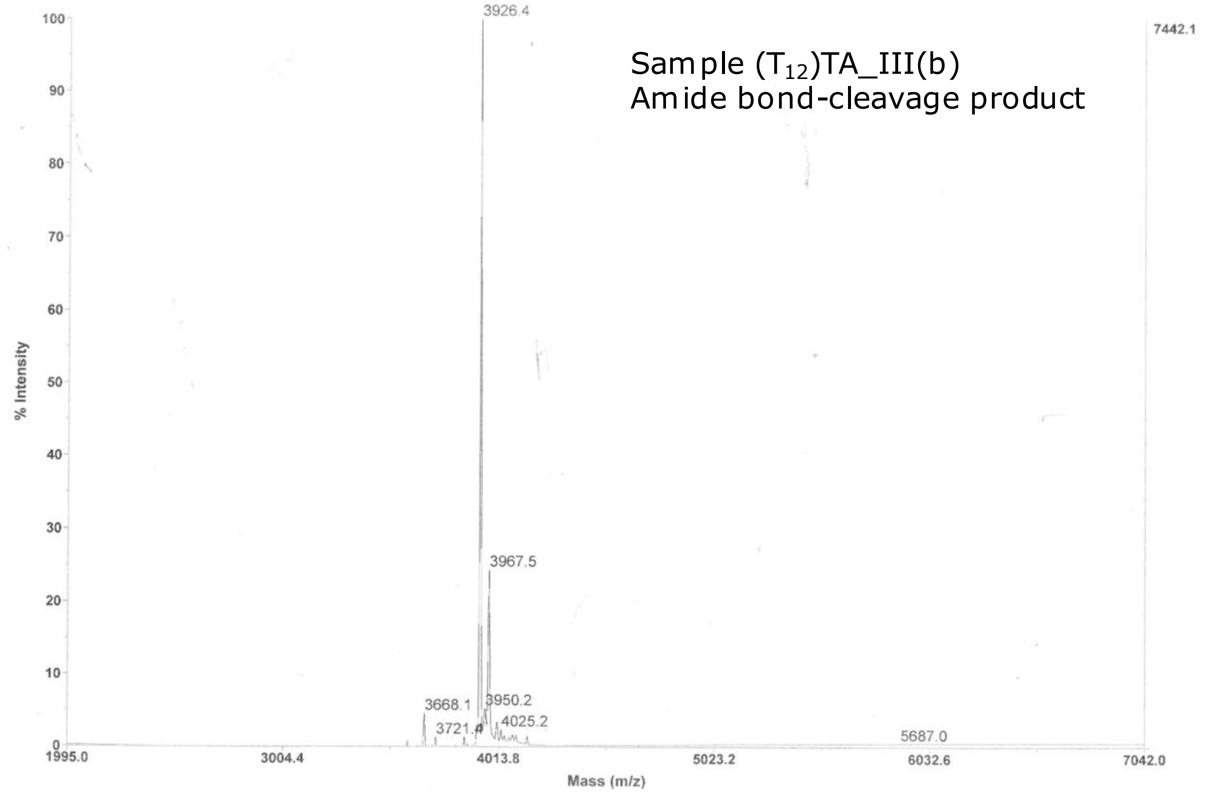

Figure S2. *Cont.*

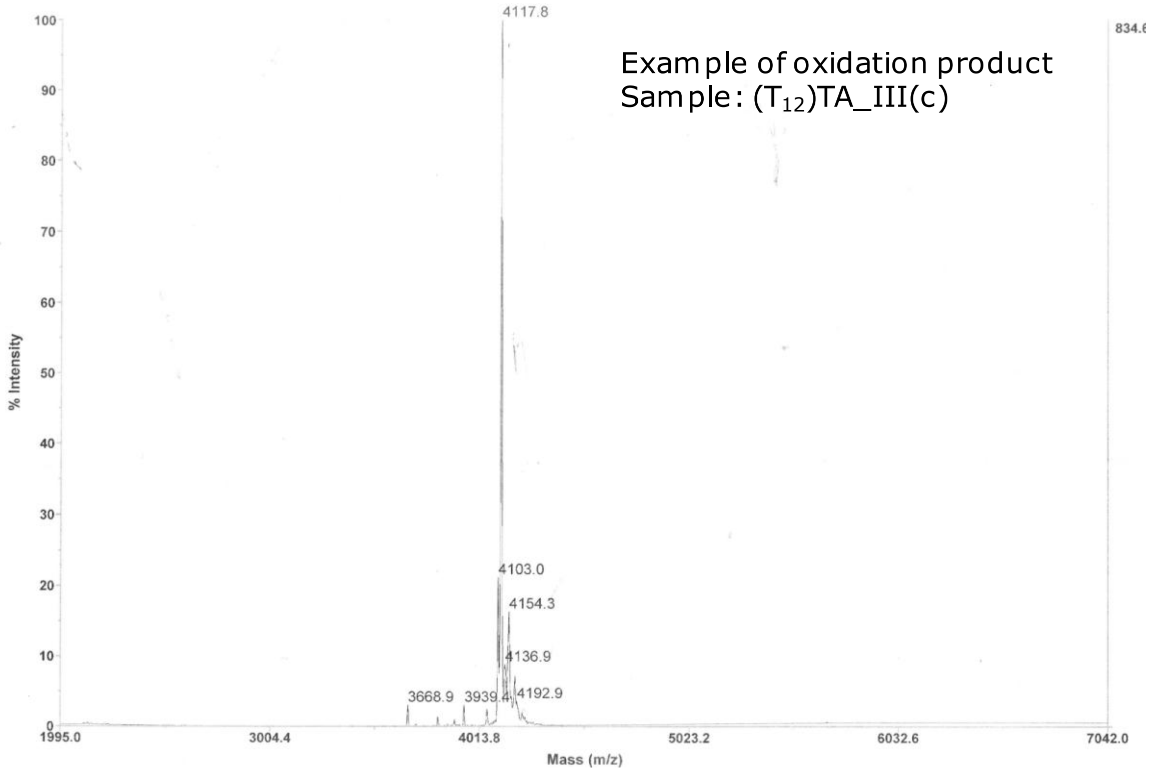

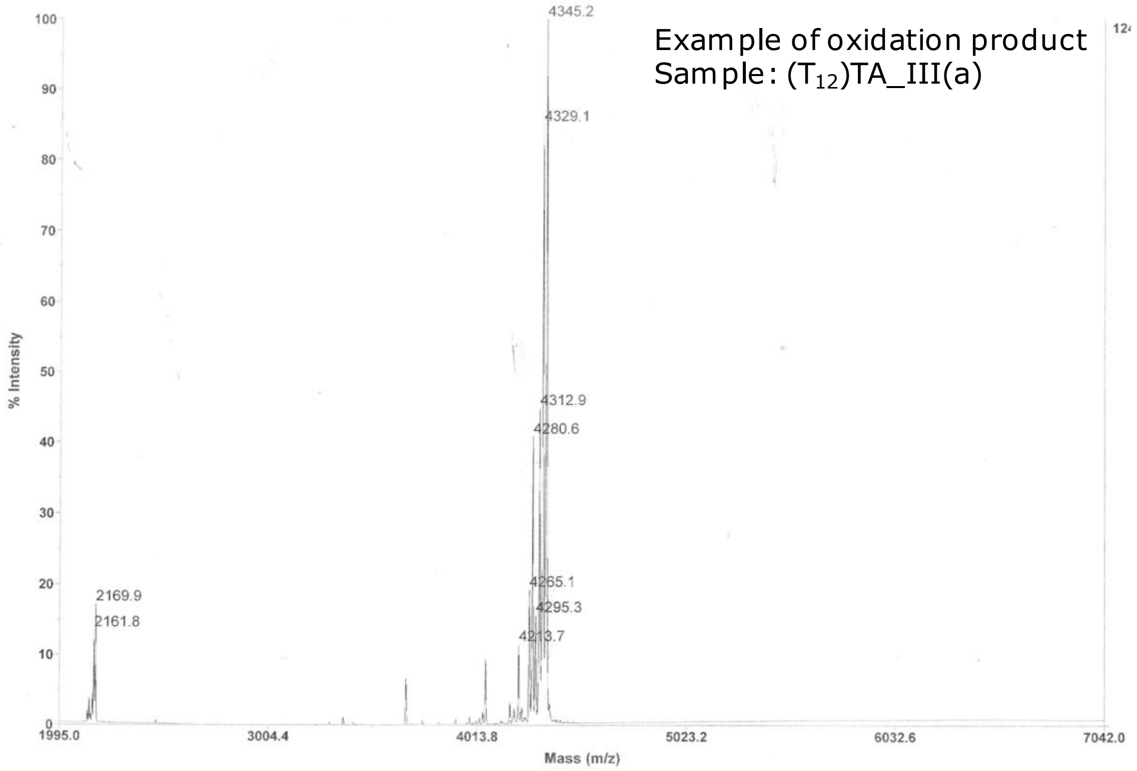

2. Mass Spectrometry Analysis (MALDI-TOF) and HPLC Chromatograms of the Purified TA-Terminated Oligonucleotides Used to Functionalize AuNp

Table S3. HPLC, Uv-vis and mass spectrometry analysis (MALDI-TOFF) of the purified TA-terminated oligonucleotides.

Oligonucleotide	Mw (calcd)	Mw (found)	t_R (min)	$\lambda_{max.}$ (nm)
**TA_I**	6508.0	6500.3	7.0	258
**TA_II**	6852.0	6848.5	8.0	258
**TA_III(a)**	6810.0	6804.5	9.4	258
**TA_III(b)**	6679.0	6672.1	7.2	258
**TA_III(c)**	6649.0	6645.6	9.5	258
**5'TA_I**	6508.0	6513.1	7.4	258
**ALK_DS**	6401.1	6402.1	5.6	258
**(F)TA_I**	7047.0	7037.1	5.4	257, 490
**(F)TA_II**	7390.9	7386.2	6.1	257, 490
**(F)TA_III(a)**	7348.9	7347.4	7.1	257, 490
**(F)TA_III(b)**	7217.9	7209.4	5.7	257, 490
**(F)TA_III(c)**	7188.0	7183.4	7.1	257, 490
**(F)ALK_DS**	6940.6	6939.8	4.1	257, 490

2.1. Threoninol-Based Oligonucleotides Containing Modified Thioctic Acid Moieties at Their 3'-Termini

TA_I

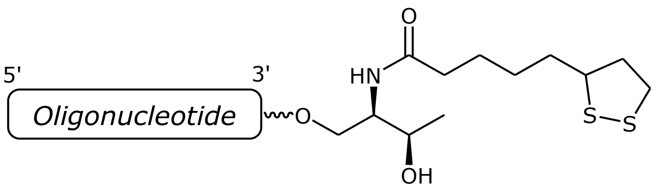

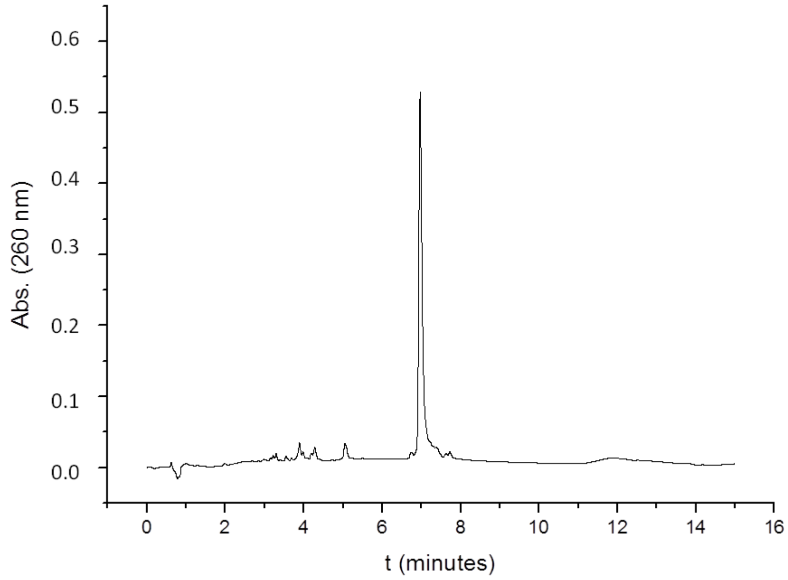

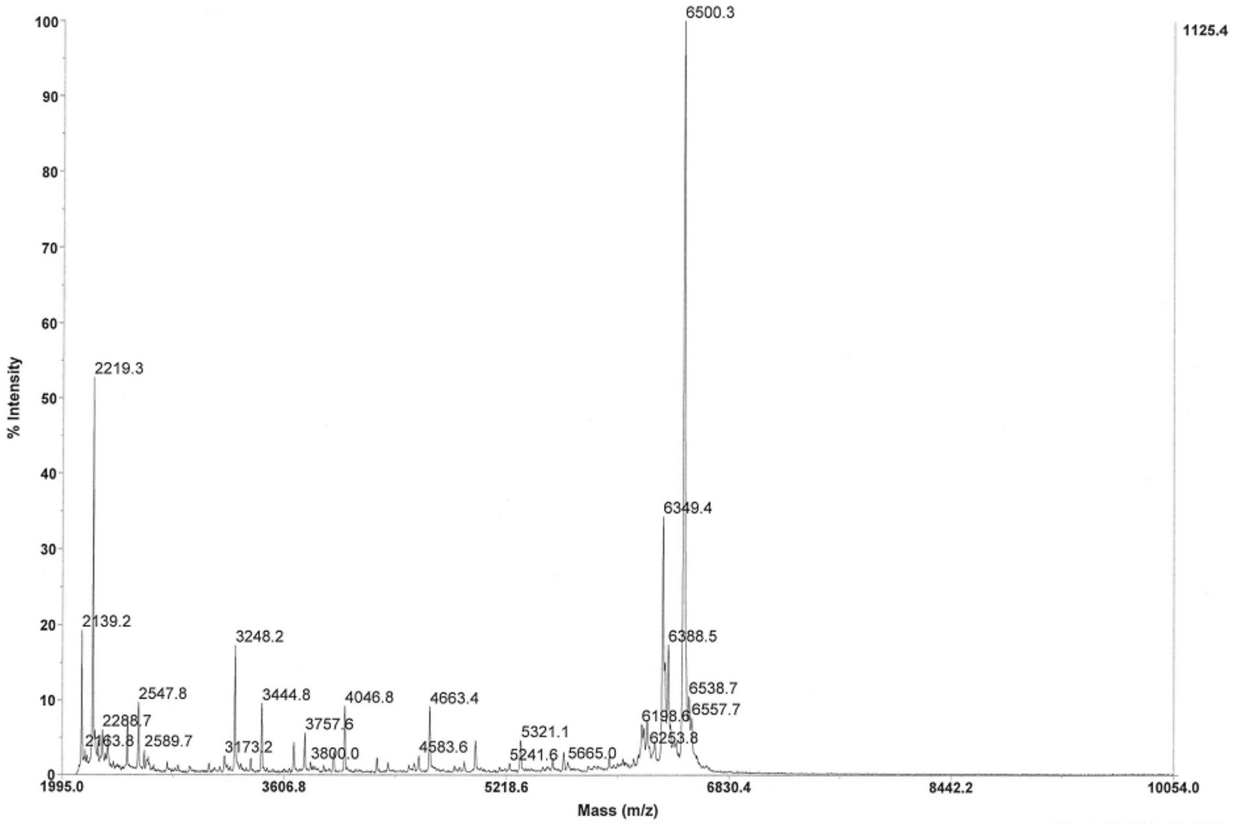

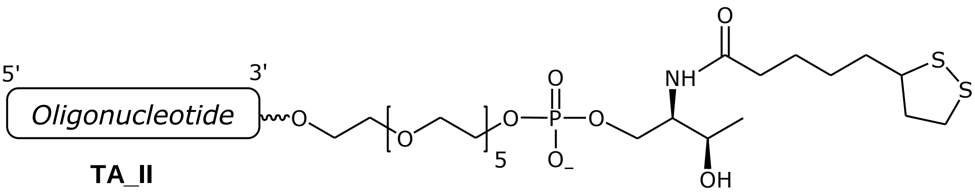
5'
3'
Oligonucleotide
O
O
5
O
P
O
O
O_
NH
O
S
S
OH

TA_II

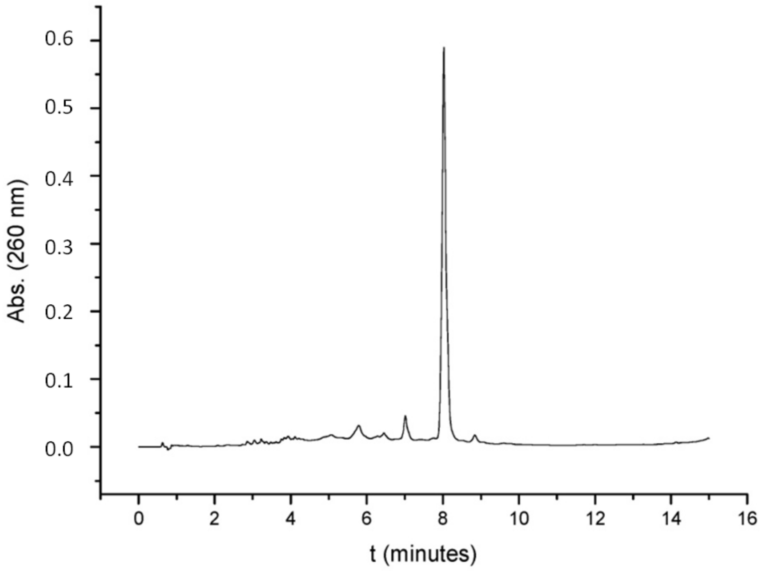
Abs. (260 nm)
t (minutes)
0.6
0.5
0.4
0.3
0.2
0.1
0.0
0
2
4
6
8
10
12
14
16

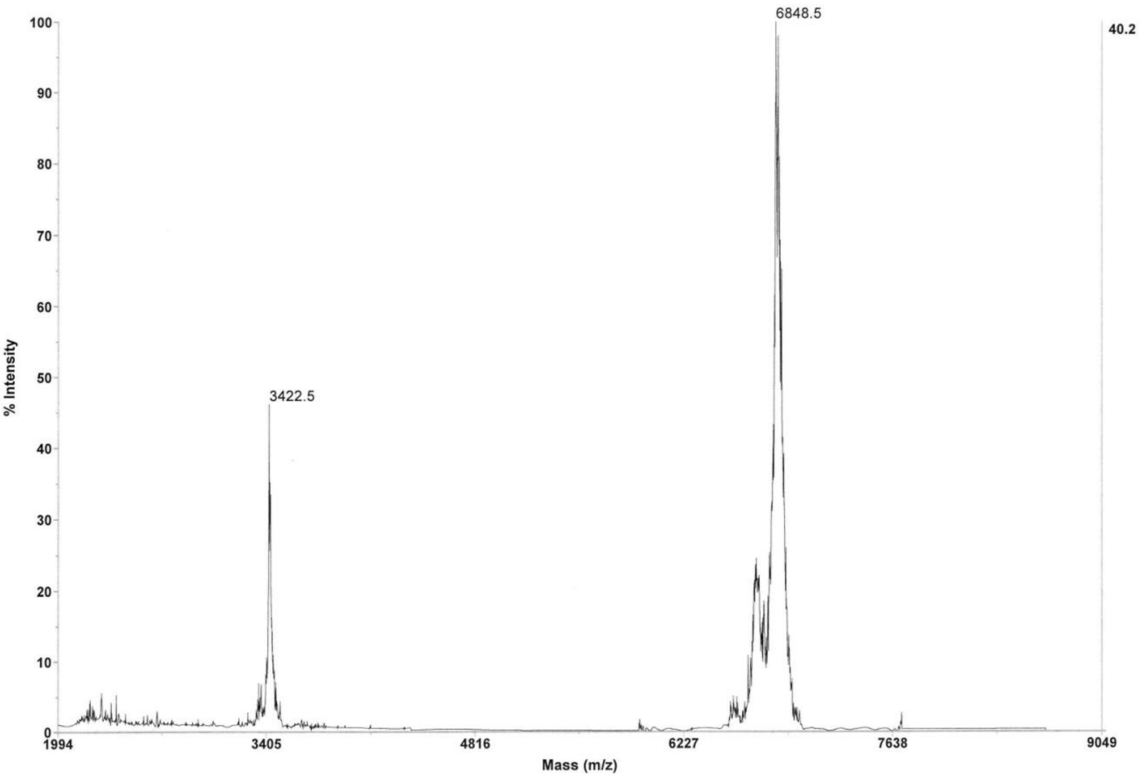
% Intensity
Mass (m/z)
6848.5
3422.5
40.2
1994
3405
4816
6227
7638
9049
100
90
80
70
60
50
40
30
20
10
0

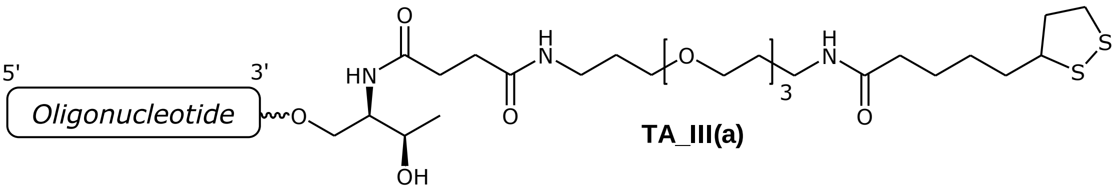
5'
3'
Oligonucleotide
TA_III(a)

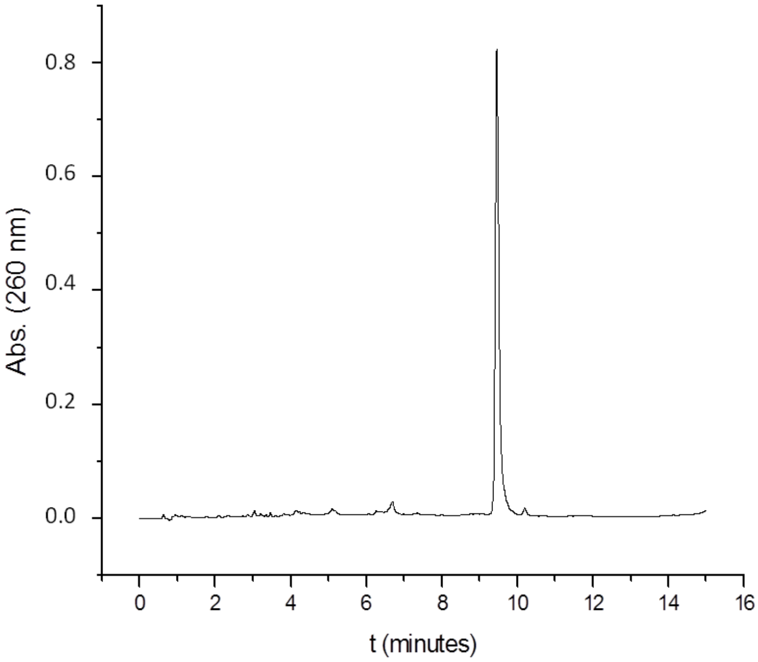
Abs. (260 nm)
t (minutes)

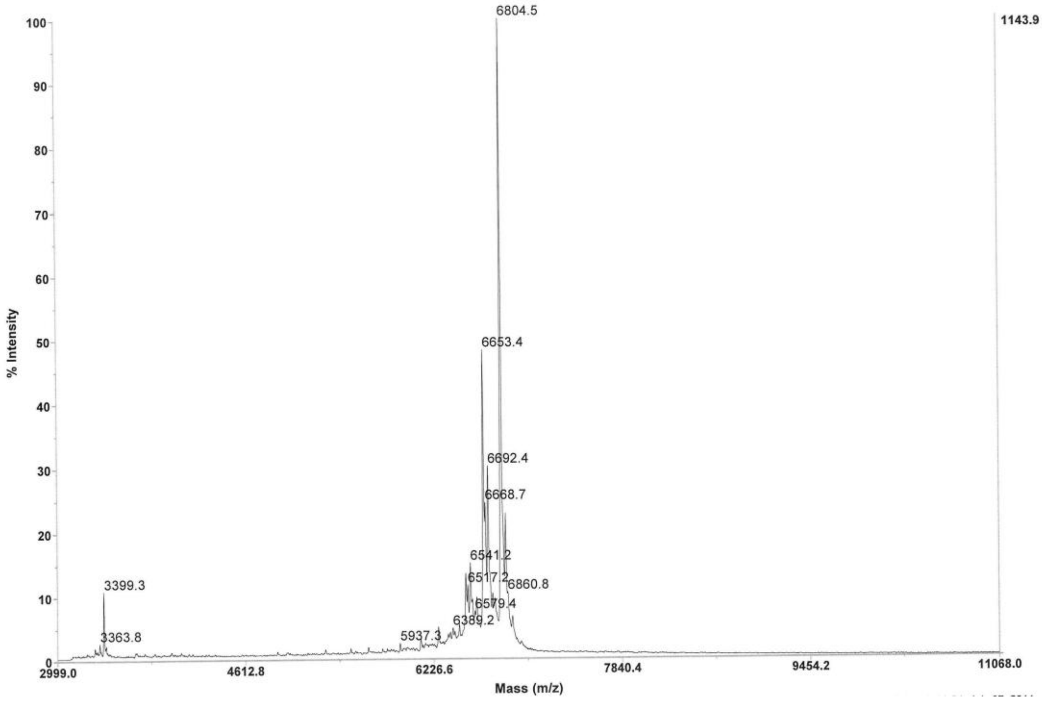
% Intensity
Mass (m/z)
6804.5
1143.9
6653.4
6692.4
6668.7
6541.2
6517.2
6860.8
6579.4
6389.2
5937.3
3399.3
3363.8

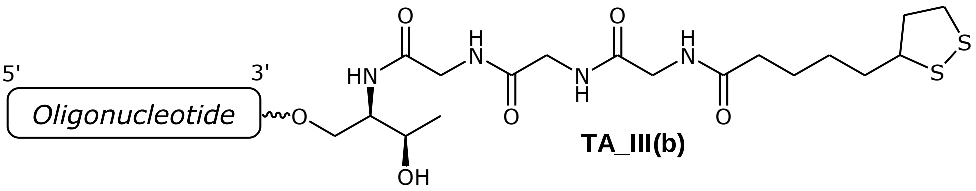
5'
3'
Oligonucleotide
HN
OH
S

TA_III(b)

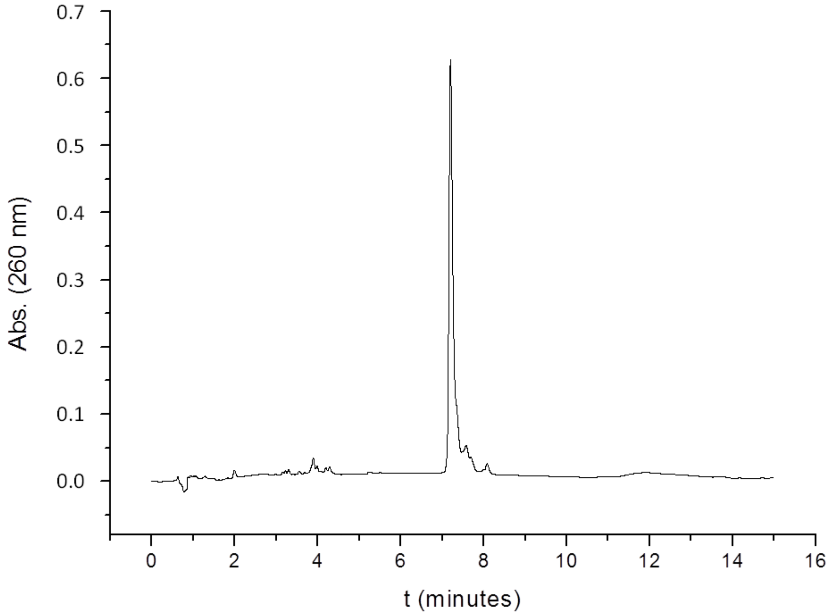
Abs. (260 nm)
t (minutes)
0.7
0.6
0.5
0.4
0.3
0.2
0.1
0.0
0
2
4
6
8
10
12
14
16

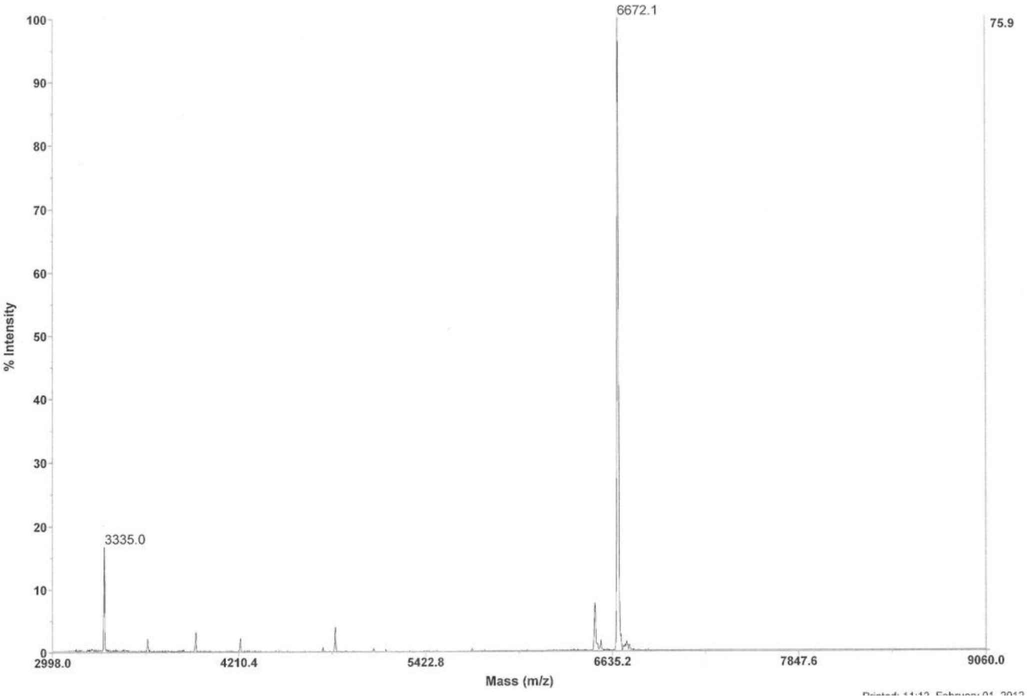
% Intensity
Mass (m/z)
6672.1
75.9
3335.0
2998.0
4210.4
5422.8
6635.2
7847.6
9060.0

TA_III(c)

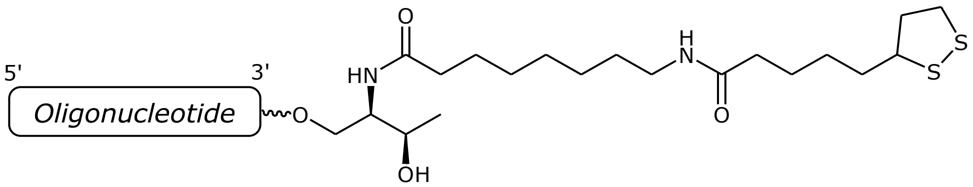
5'
3'
Oligonucleotide
O
HN
O
H
N
O
S
S
OH

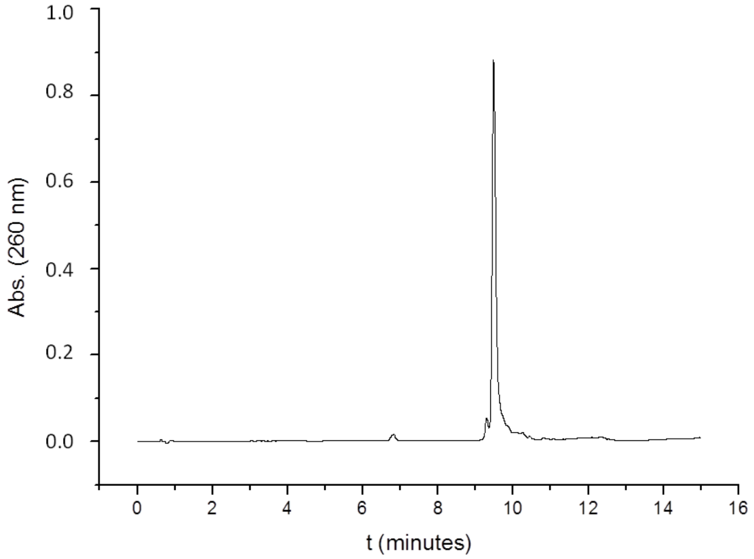
1.0
0.8
0.6
0.4
0.2
0.0
Abs. (260 nm)
0
2
4
6
8
10
12
14
16
t (minutes)

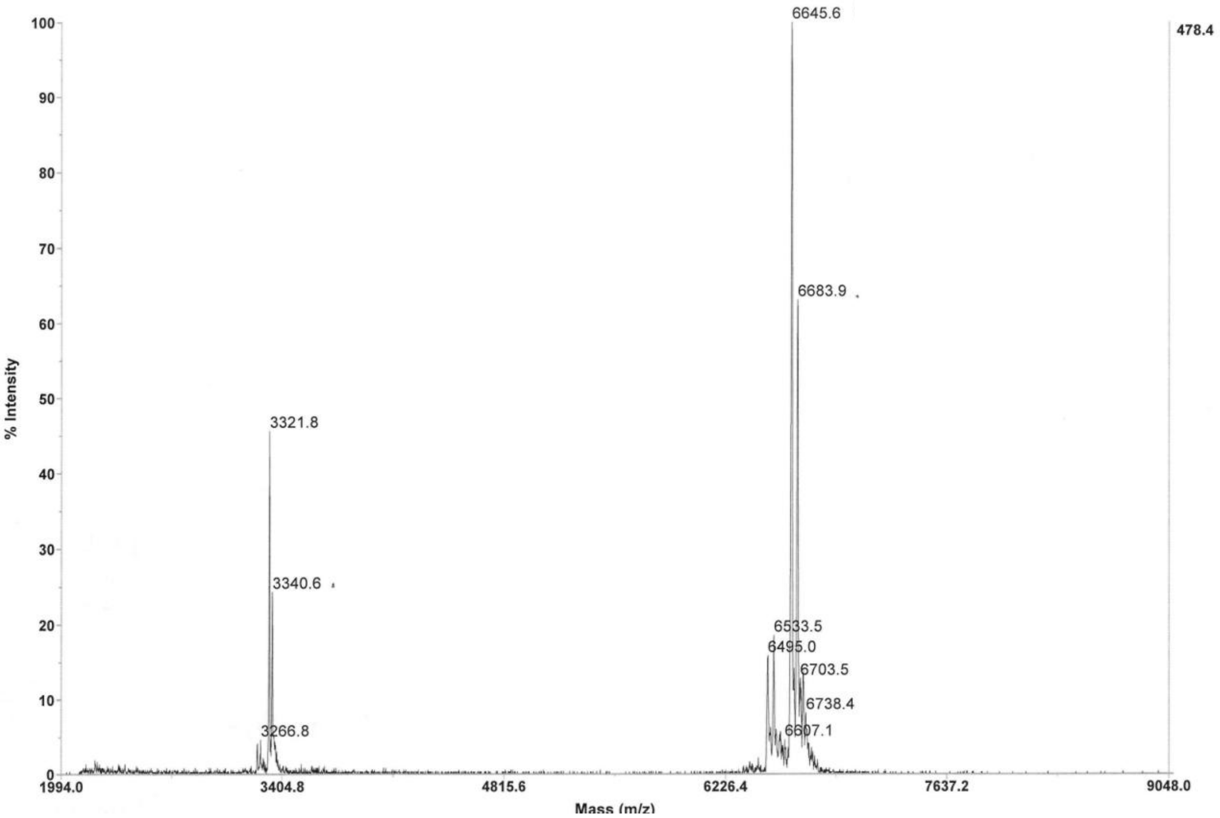
100
90
80
70
60
50
40
30
20
10
0
% Intensity
6645.6
478.4
6683.9
3321.8
3340.6
6533.5
6495.0
6703.5
6738.4
6607.1
3266.8
1994.0
3404.8
4815.6
6226.4
7637.2
9048.0
Mass (m/z)

5'TA_I

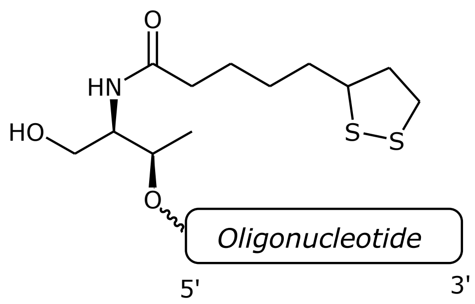
O
HN
HO
S
S
O
Oligonucleotide
5'
3'

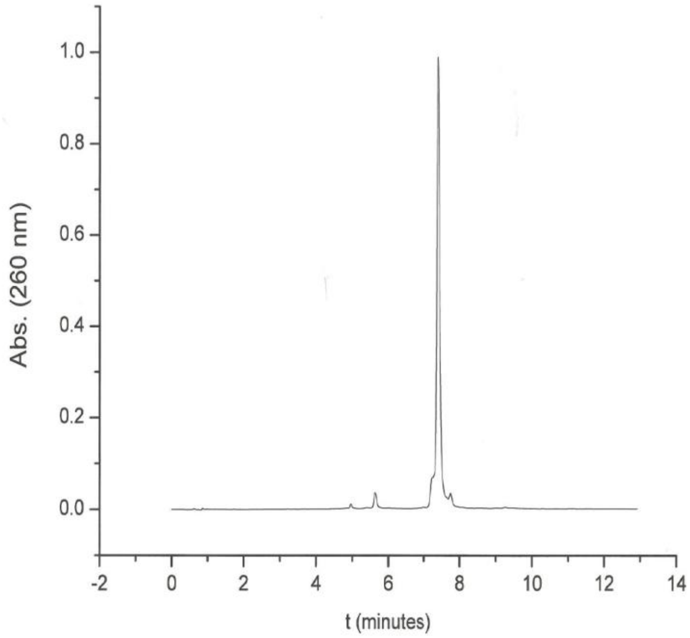
Abs. (260 nm)
1.0
0.8
0.6
0.4
0.2
0.0
-2
0
2
4
6
8
10
12
14
t (minutes)

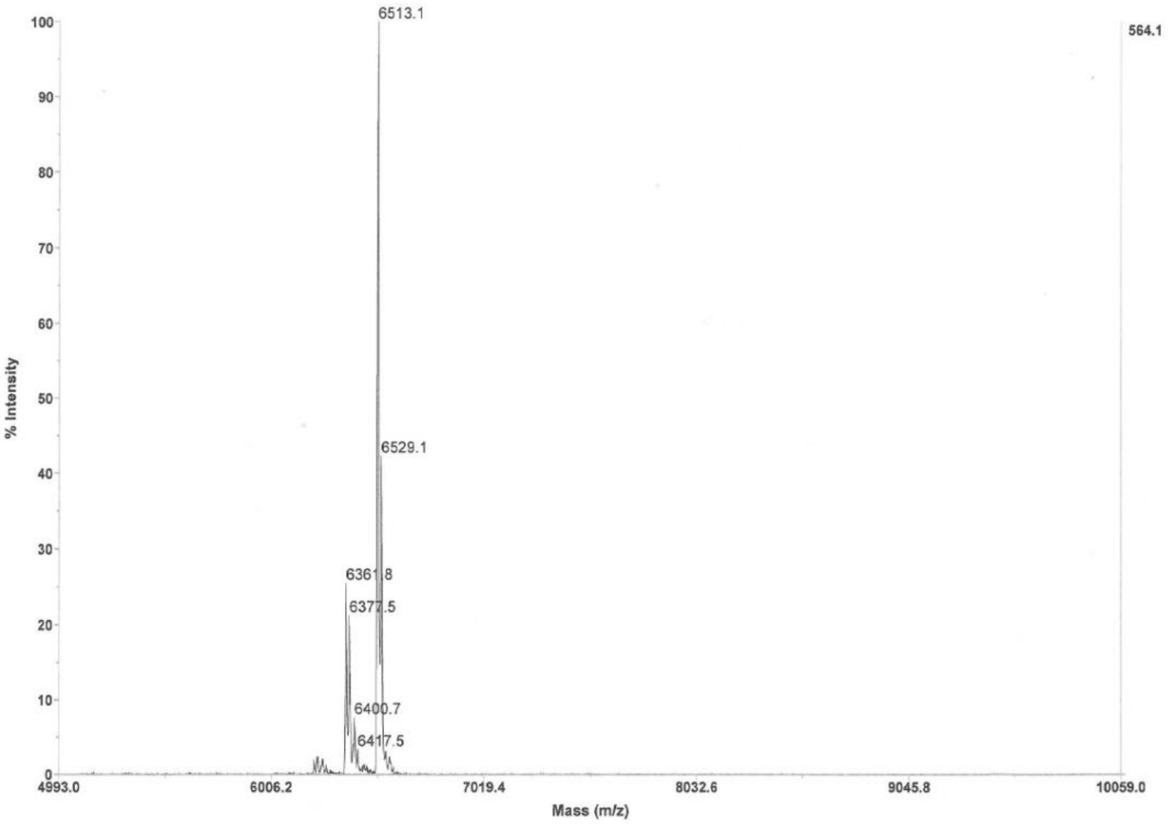
% Intensity
Mass (m/z)
6513.1
6529.1
6361.8
6377.5
6400.7
6417.5
564.1
4993.0
6006.2
7019.4
8032.6
9045.8
10059.0
100
90
80
70
60
50
40
30
20
10
0

ALK_DS

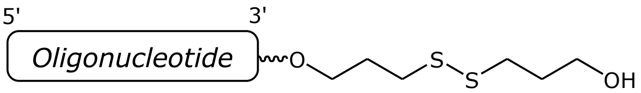
5'
3'
Oligonucleotide
O
S
S
OH

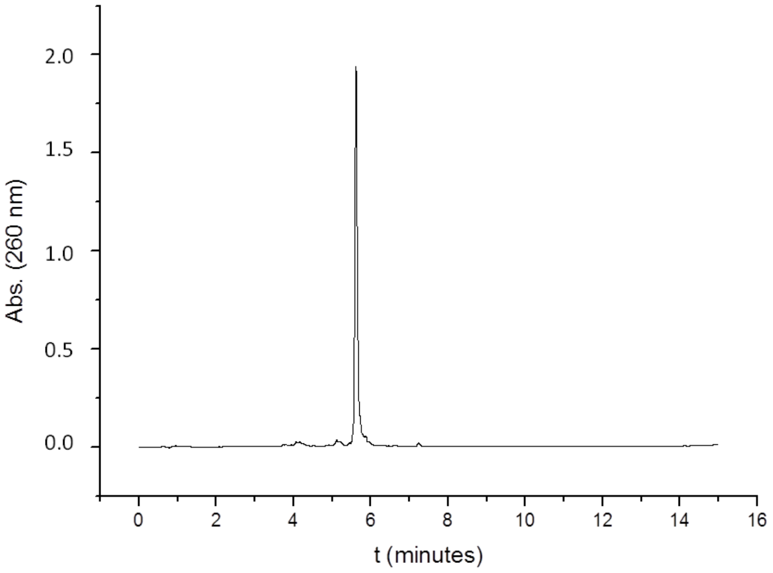
Abs. (260 nm)
2.0
1.5
1.0
0.5
0.0
0
2
4
6
8
10
12
14
16
t (minutes)

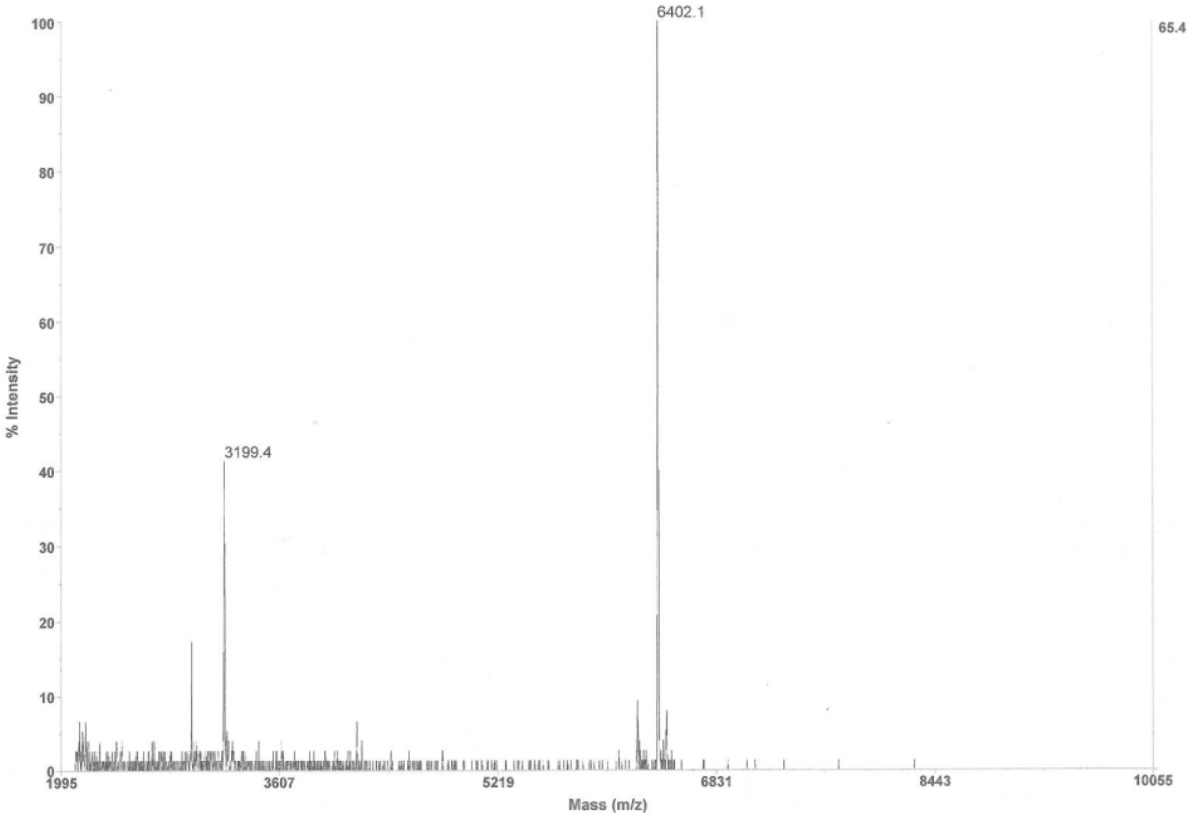
% Intensity
100
90
80
70
60
50
40
30
20
10
0
6402.1
3199.4
65.4
1995
3607
5219
6831
8443
10055
Mass (m/z)

2.2. Threoninol-Based Oligonucleotides Containing Modified Thioctic Acid Moieties at Their 3'-Termini and Fluorescein at 5'-Termini

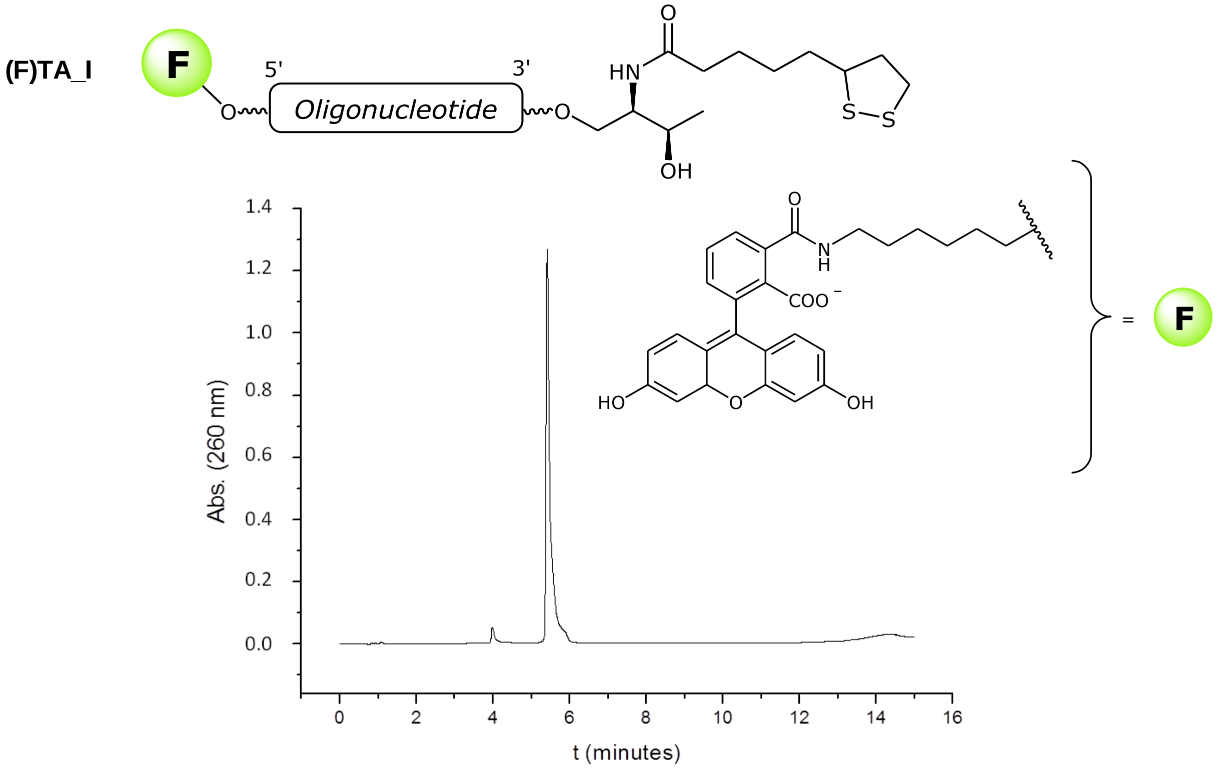

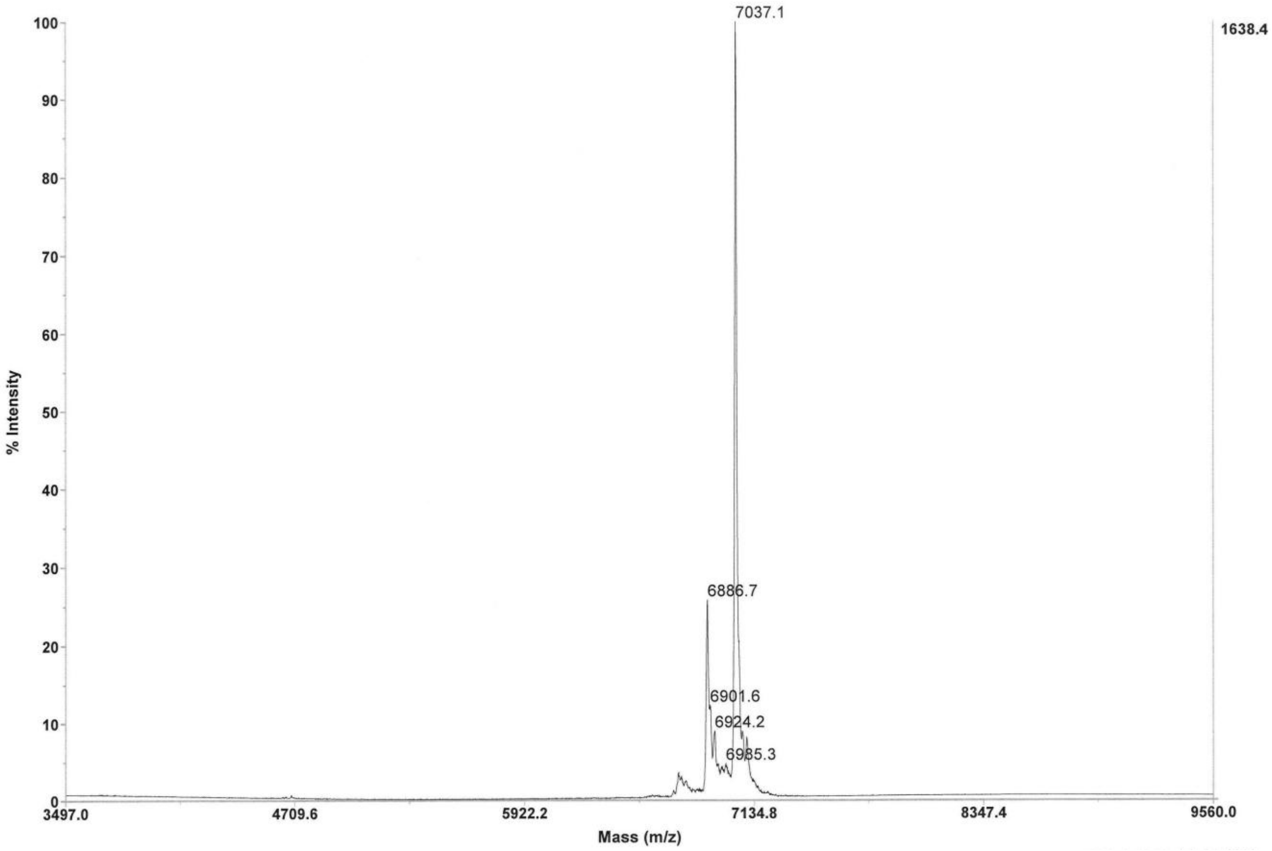

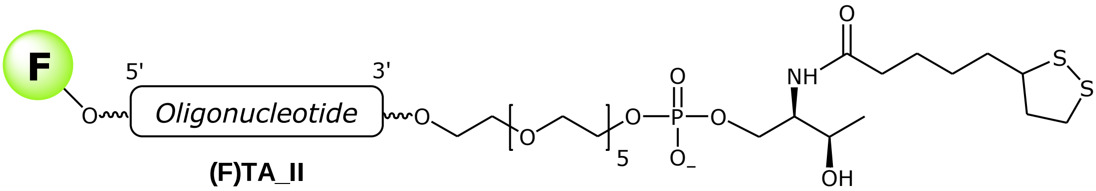
F
5'
3'
Oligonucleotide
O
NH
OH
S
S
5

(F)TA_II

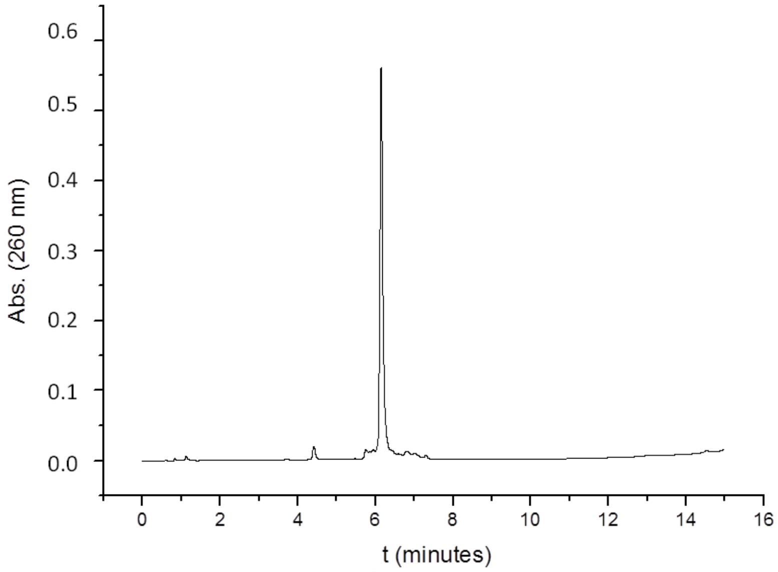
Abs. (260 nm)
0.6
0.5
0.4
0.3
0.2
0.1
0.0
0
2
4
6
8
10
12
14
16
t (minutes)

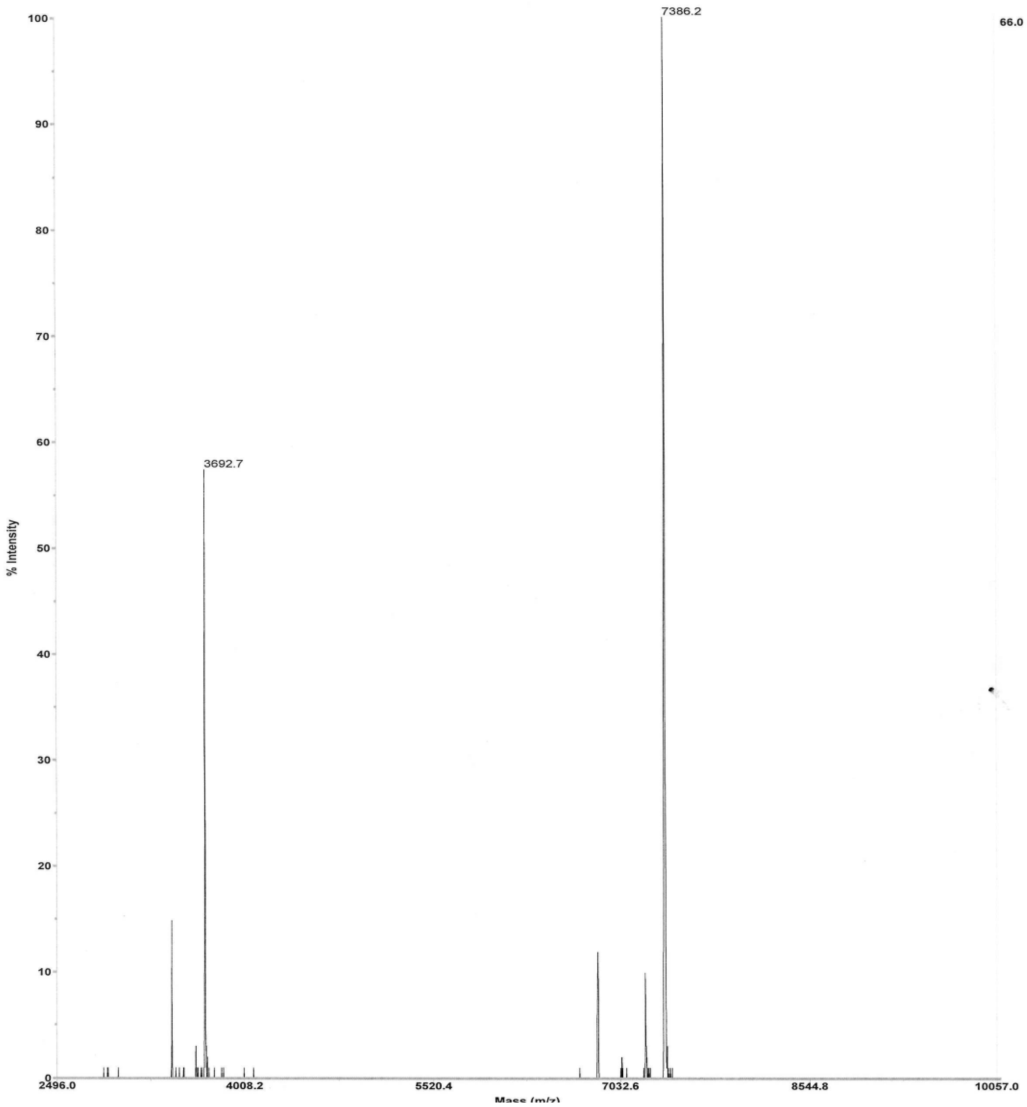
7386.2
66.0
3692.7
% Intensity
100
90
80
70
60
50
40
30
20
10
0
2496.0
4008.2
5520.4
7032.6
8544.8
10057.0
Mass (m/z)

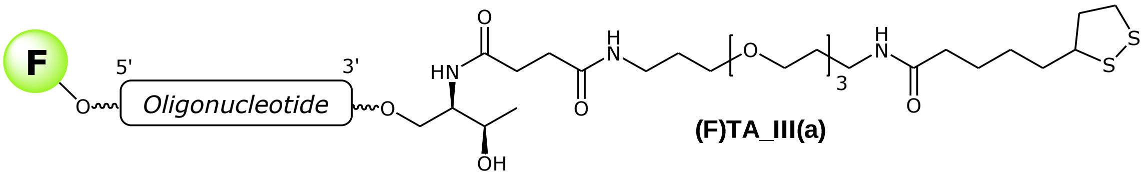
F
5'
3'
Oligonucleotide
(F)TA_III(a)

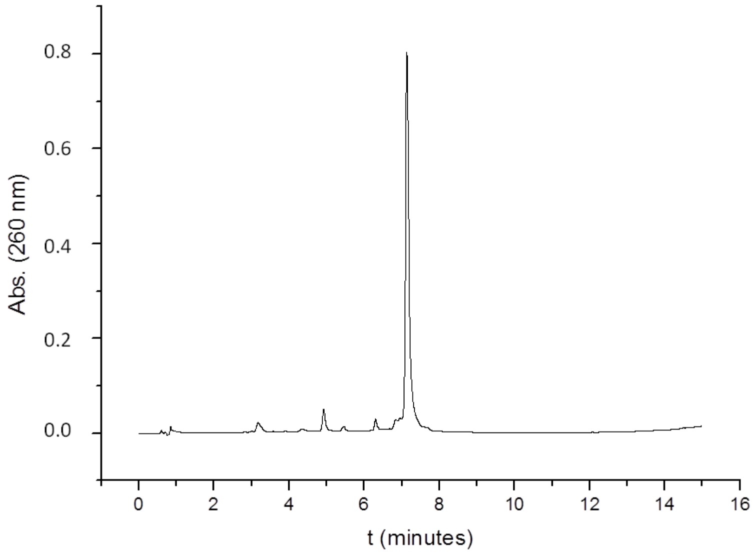
Abs. (260 nm)
t (minutes)

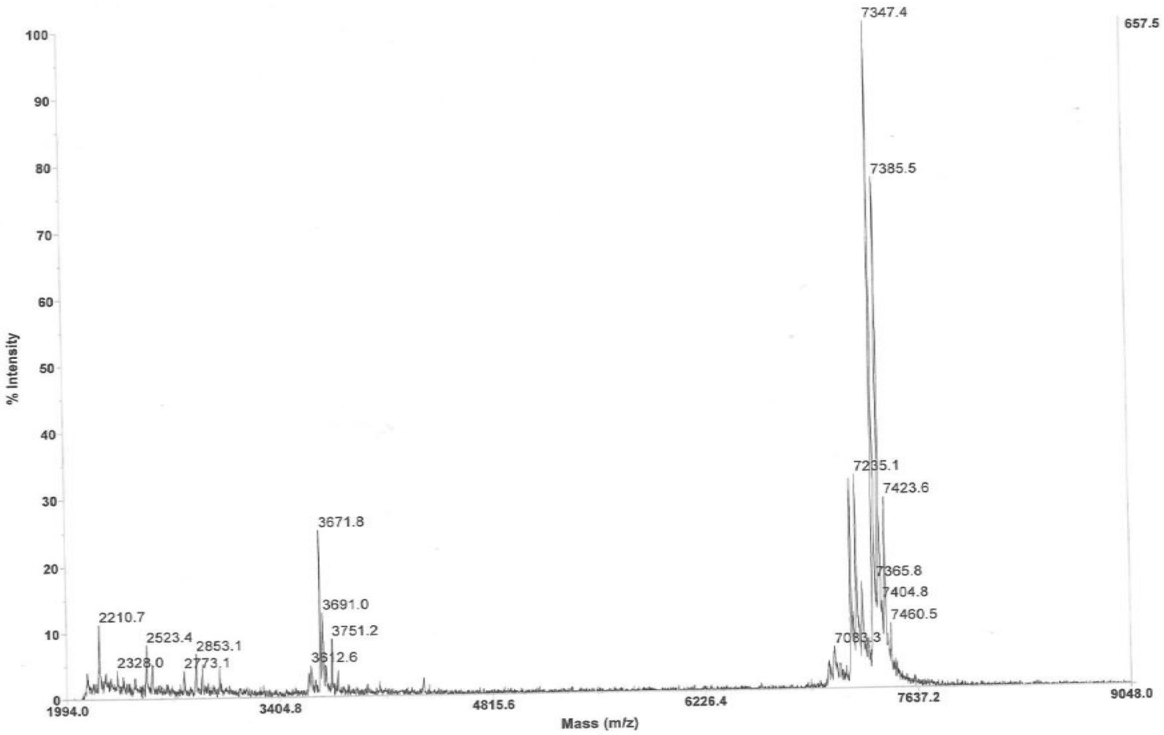
% Intensity
Mass (m/z)
7347.4
7385.5
7235.1
7423.6
3671.8
2210.7
657.5

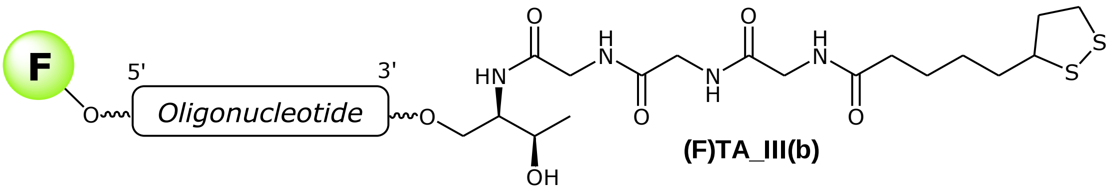
F
5'
3'
Oligonucleotide
HN
OH
O
H
N
N
H
S
S

(F)TA_III(b)

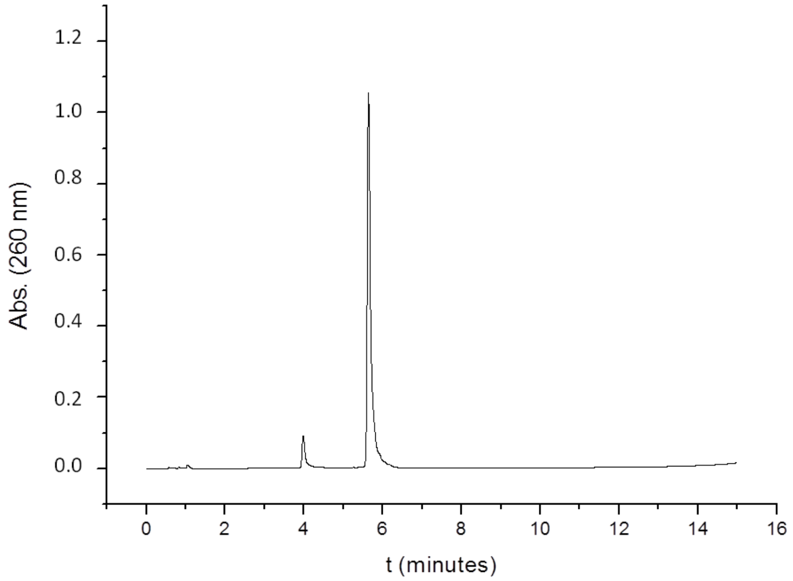
Abs. (260 nm)
1.2
1.0
0.8
0.6
0.4
0.2
0.0
0
2
4
6
8
10
12
14
16
t (minutes)

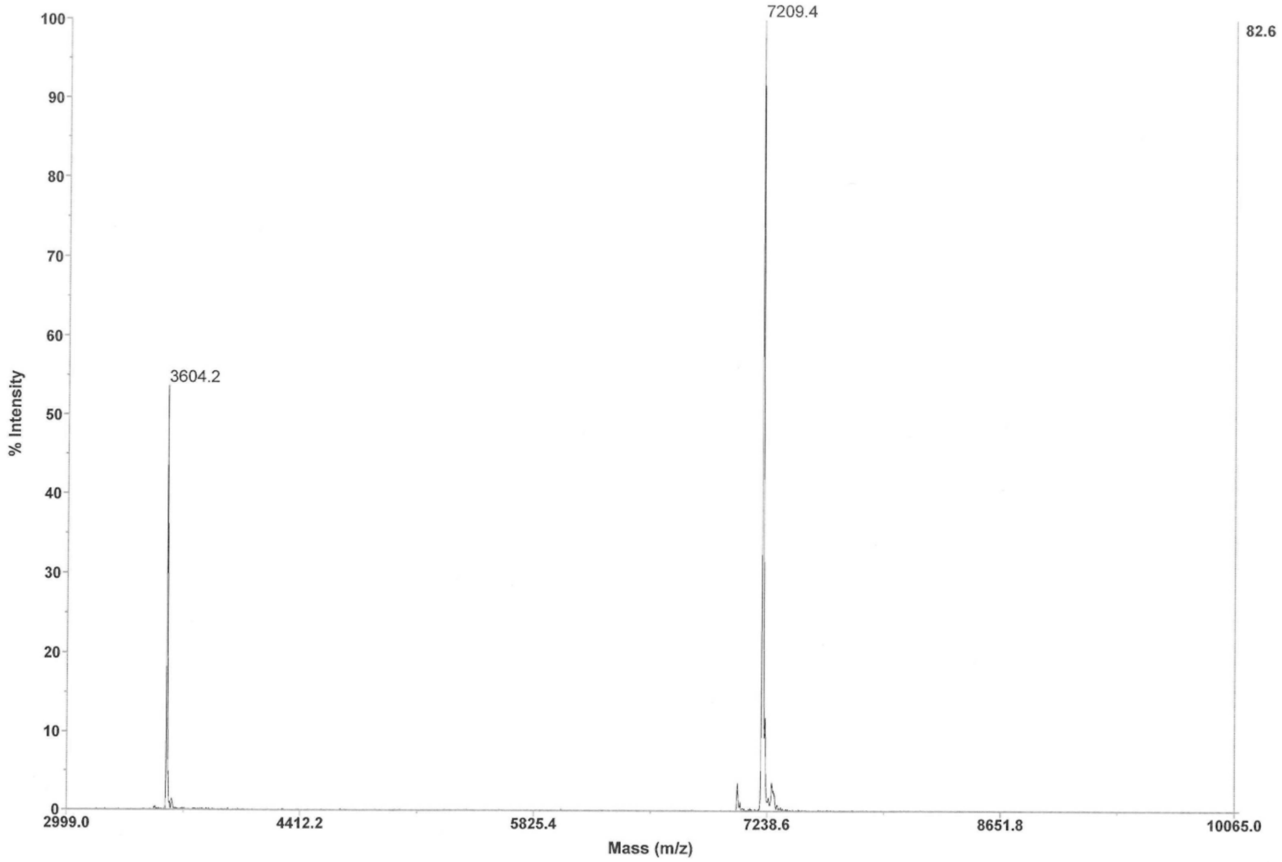
% Intensity
100
90
80
70
60
50
40
30
20
10
0
7209.4
82.6
3604.2
2999.0
4412.2
5825.4
7238.6
8651.8
10065.0
Mass (m/z)

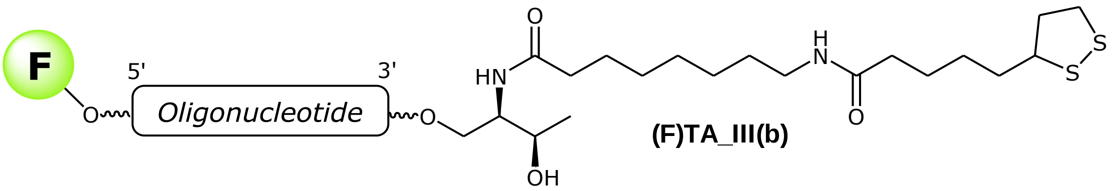
F
5'
Oligonucleotide
3'
O
HN
O
OH
H
N
O
S
S
(F)TA_III(b)

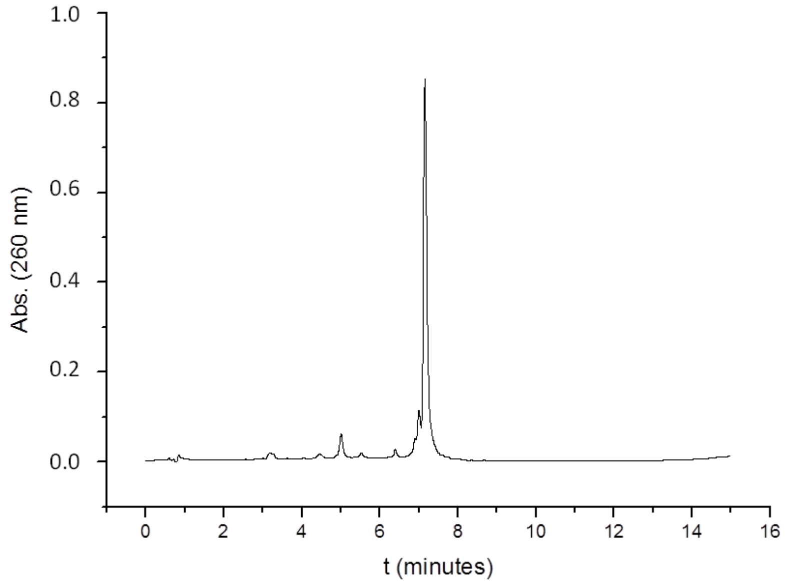
Abs. (260 nm)
t (minutes)

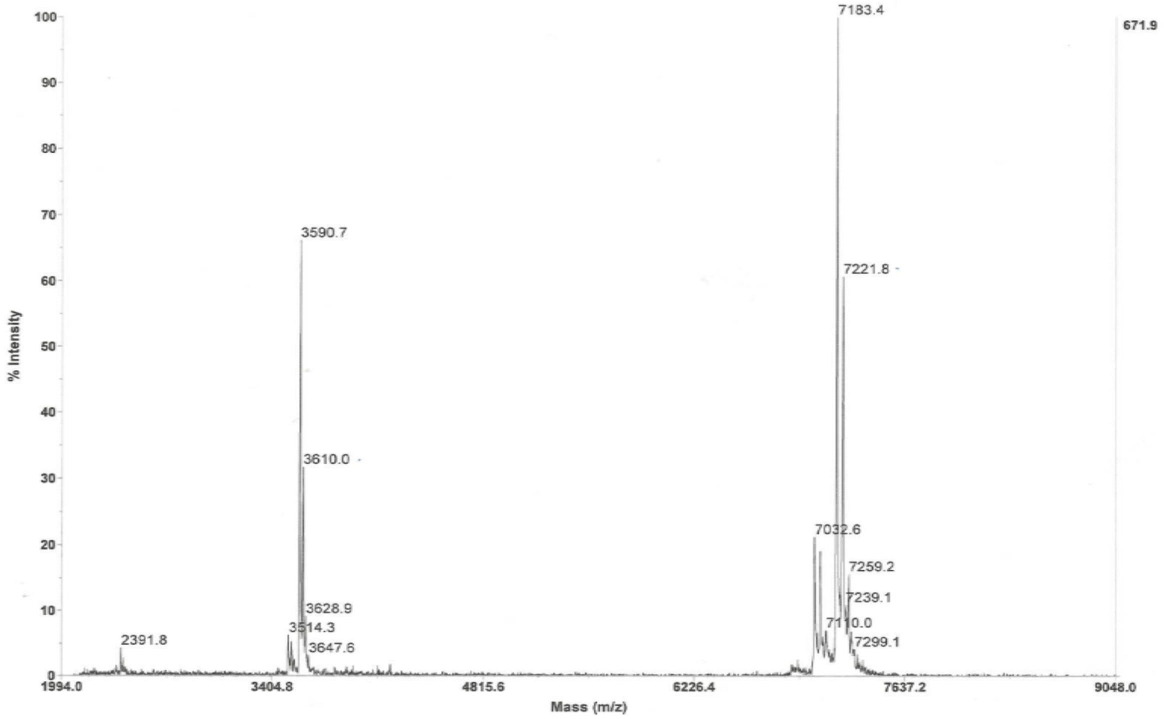
% Intensity
Mass (m/z)
7183.4
7221.8
3590.7
3610.0
7032.6
7259.2
7239.1
7110.0
7299.1
3628.9
3514.3
3647.6
2391.8
671.9

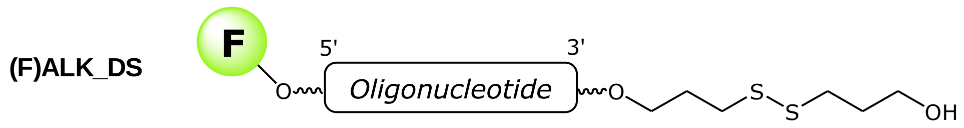
(F)ALK_DS
F
5'
3'
Oligonucleotide
O
O
S
S
OH

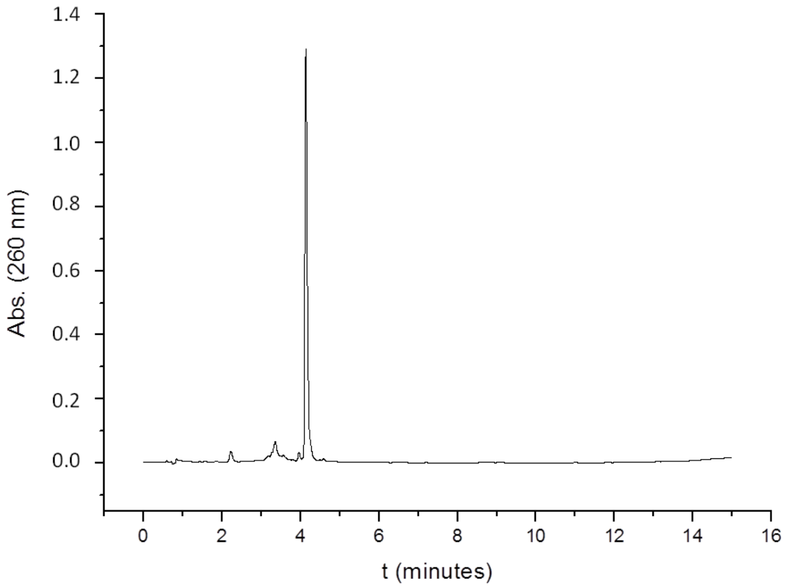
Abs. (260 nm)
t (minutes)
1.4
1.2
1.0
0.8
0.6
0.4
0.2
0.0
0
2
4
6
8
10
12
14
16

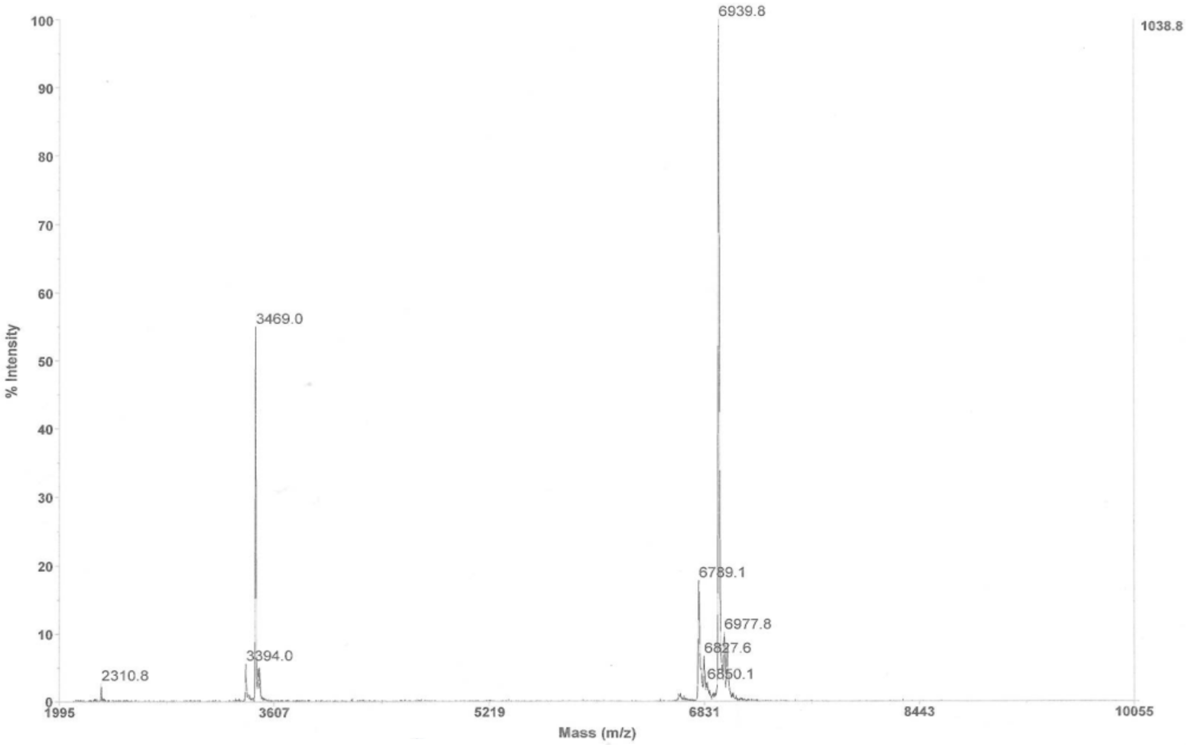
% Intensity
Mass (m/z)
6939.8
1038.8
3469.0
6789.1
6977.8
6827.6
6850.1
3394.0
2310.8
1995
3607
5219
6831
8443
10055
100
90
80
70
60
50
40
30
20
10
0

3. HPLC and Mass Spectrometry Analysis (MALDI-TOF) Obtained from the Treatment of TA_Terminated Oligonucleotides with Cathepsin B

Table S4. Results obtained with the different TA-terminated oligonucleotides in the presence of Cathepsin B and mass spectrometry analysis (MALDI-TOF).

	Reaction Time (h)	% Amide Bond Cleavage	t_R (min.)	Mass Found	Mass Calcdt.
	2	0.8			
	4	2			
**TA_III(b)**	24	15	9.9	6429.8	6433.0
	48	26			
	72	25			
**TA_III(a)**	72	7	10.4	6618.1	6621.0
**TA_III(c)**	72	12	10.3	6456.4	6460.0

Figure S5. HPLC profiles obtained after treatment the TA-terminated oligonucleotides with Cathepsin B: (**A**) **TA_III(b)** at different times; (**B**) **TA_III(c)**; (**C**) **TA_III(a)**; (**D**) **TA_II** and (**E**) **TA_I** at 72 h (red lines). In all cases the black lines stand for a negative control (the same experimental conditions but without the enzyme). ■ stands for the TA-terminated oligonucleotide and ▼ stands for the cleavage product generated in each case.

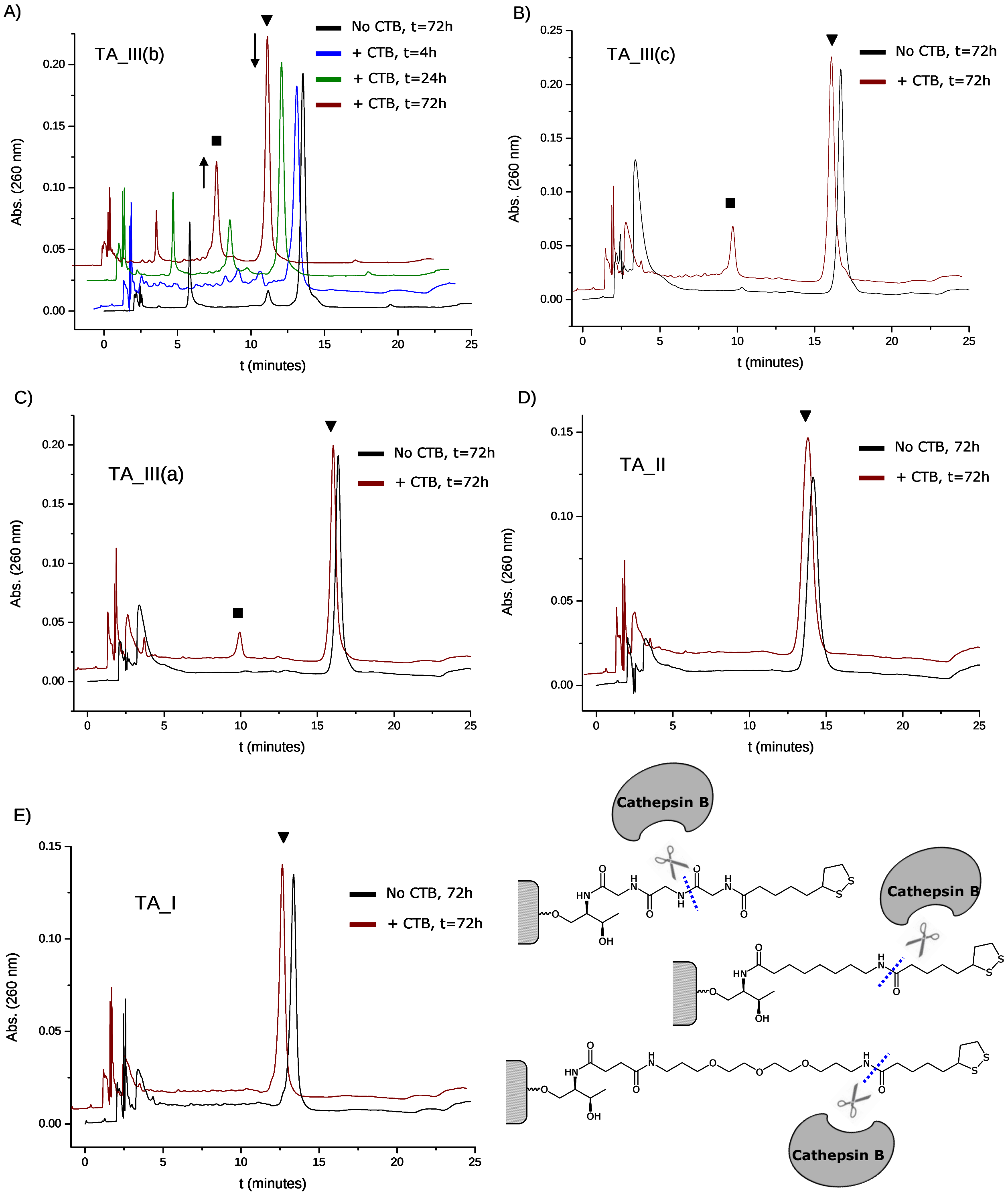

Figure S6. MALDI-TOFF mass spectrometry of the amide bond cleavage products.

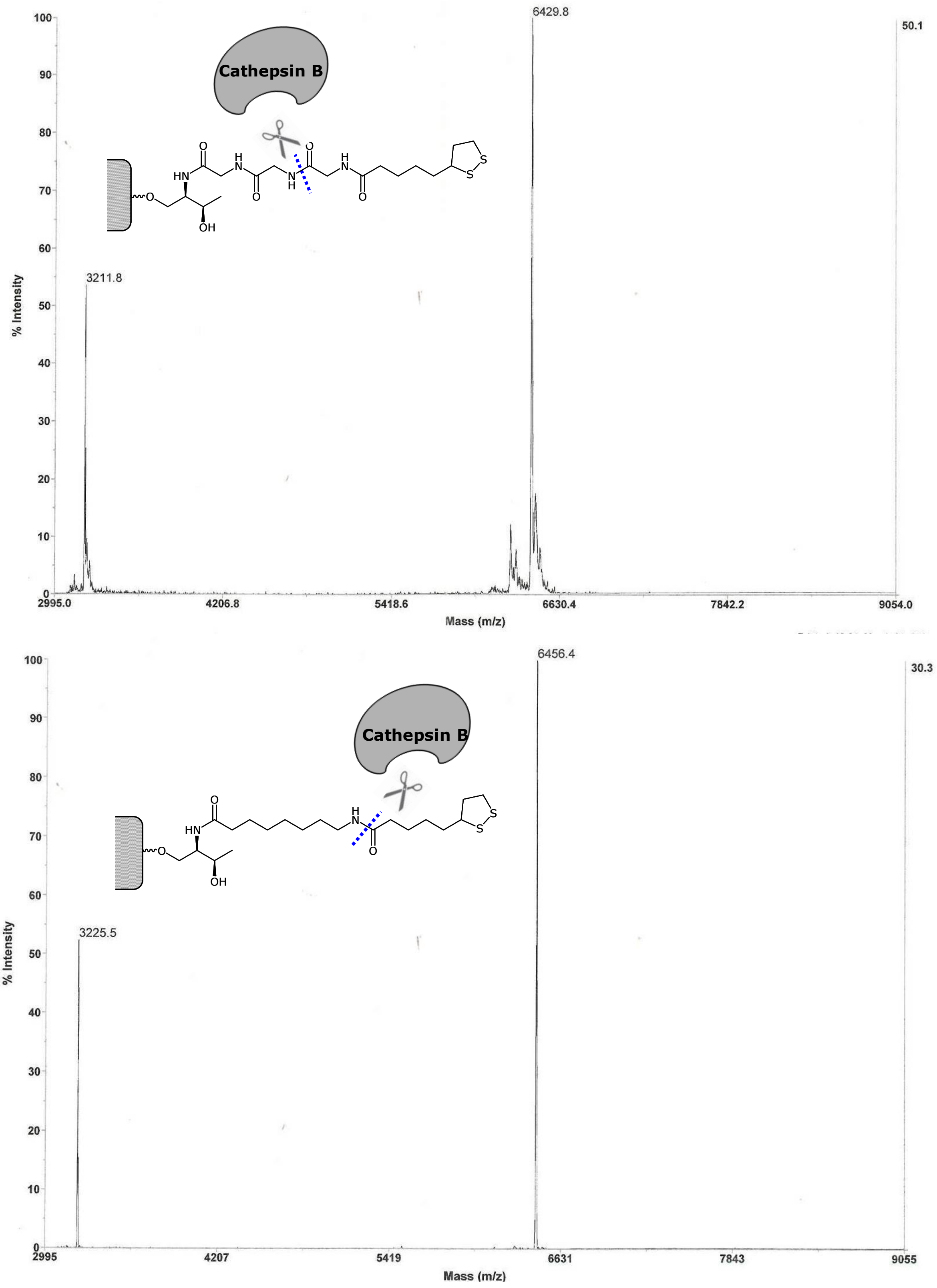

Figure S6. *Cont.*

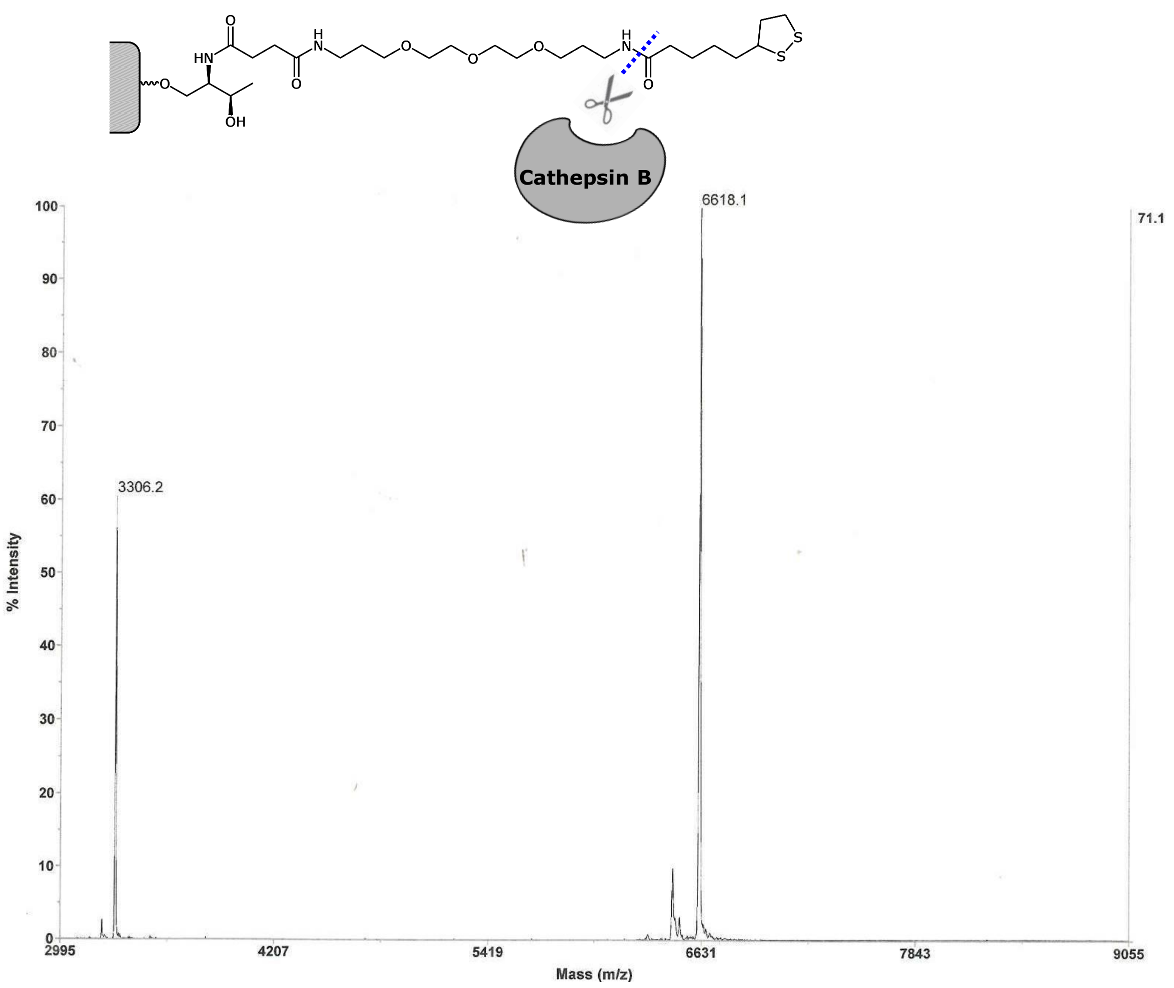

4. Characterization (^{1}H-NMR, ^{13}C-NMR and ^{31}P-NMR) of the Synthesized Threoninol-Based Analogues

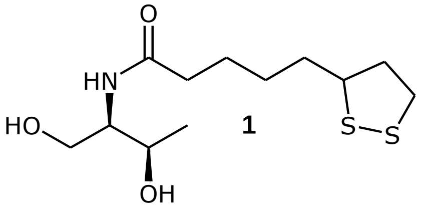

1

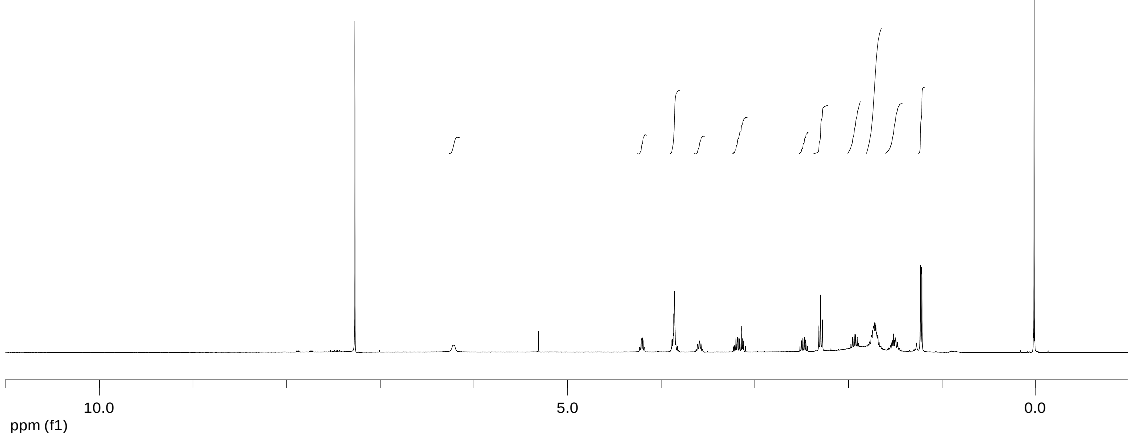

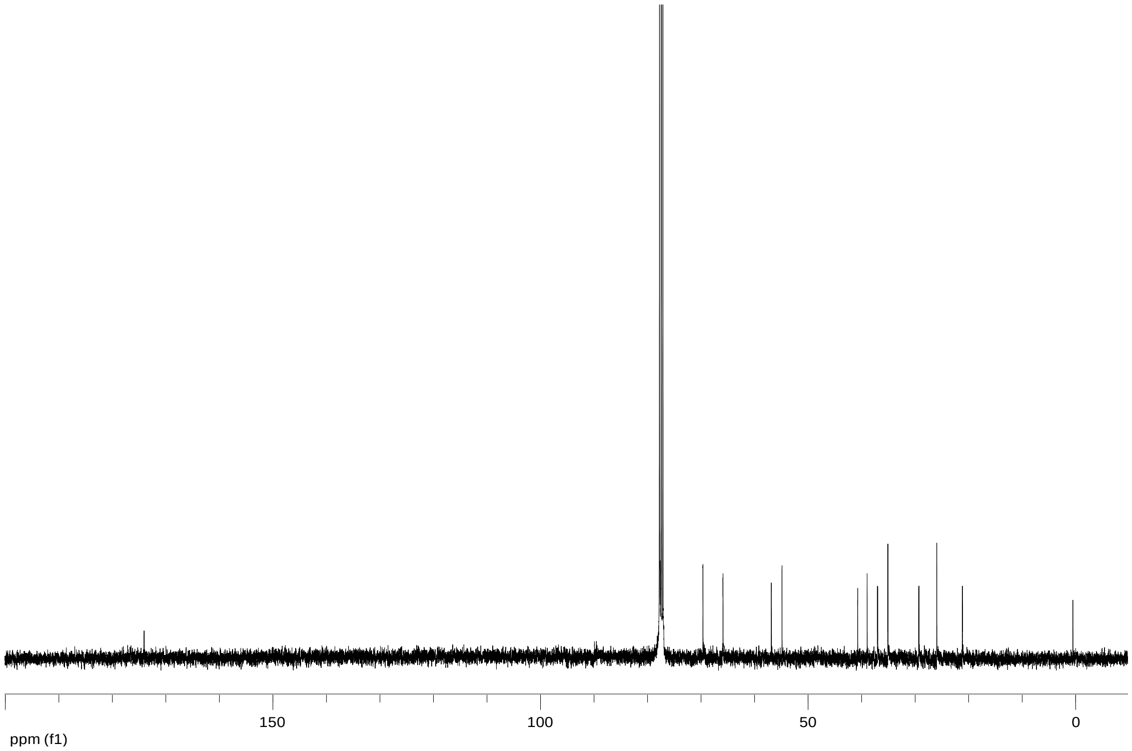

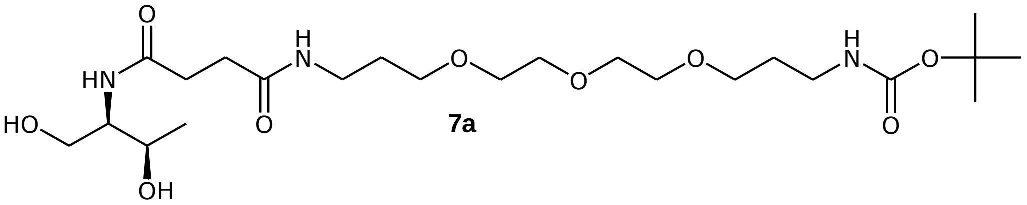

7a

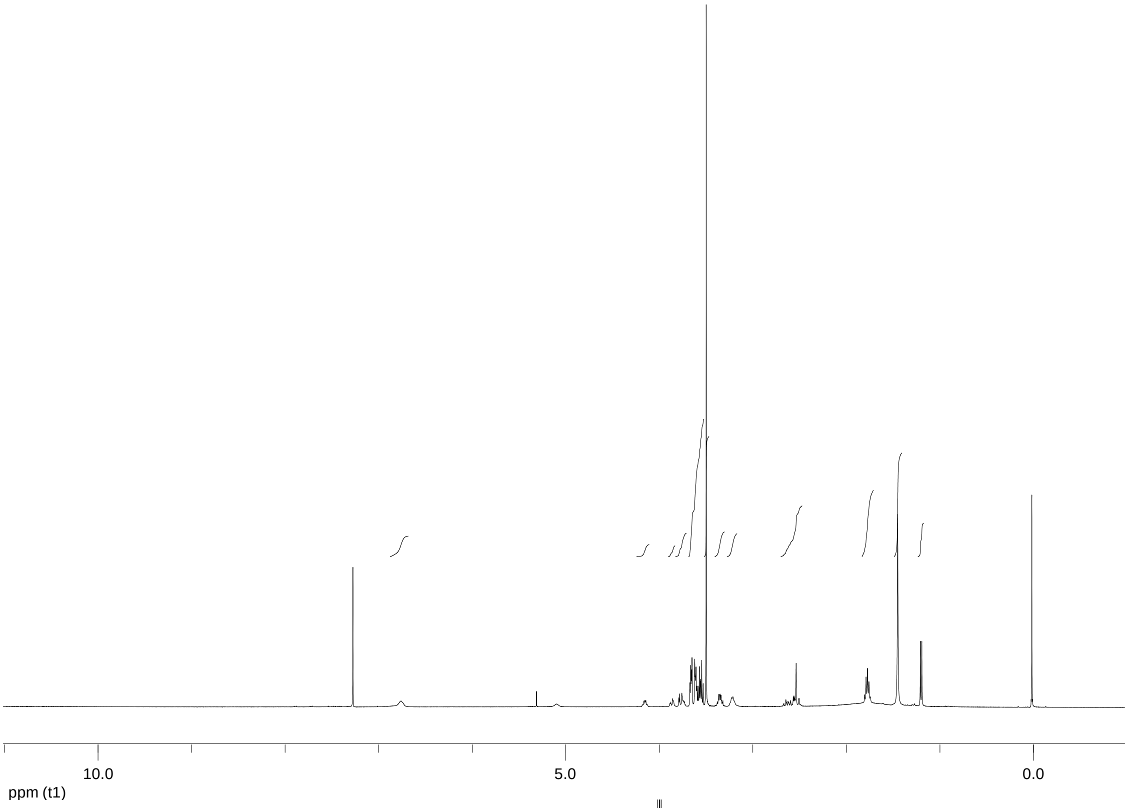

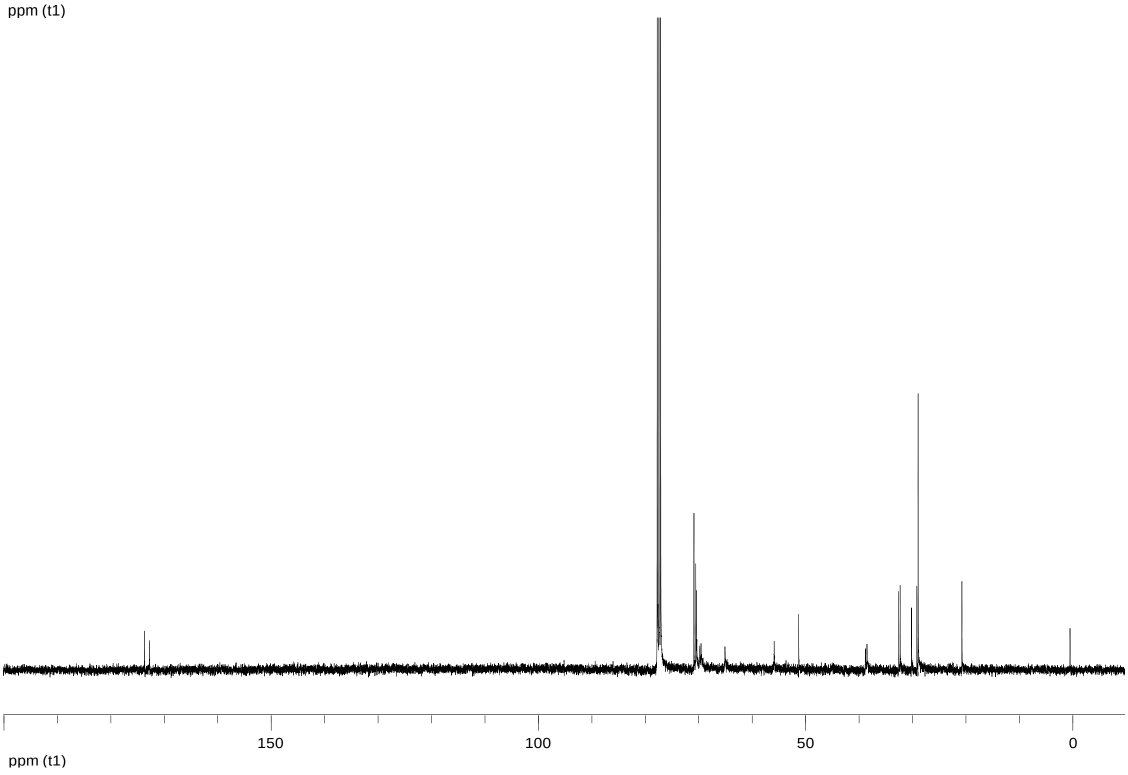

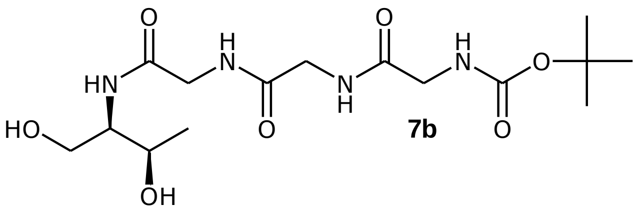

HN
HO
OH
O
H
N
O
O
N
H
H
N
O
O

7b

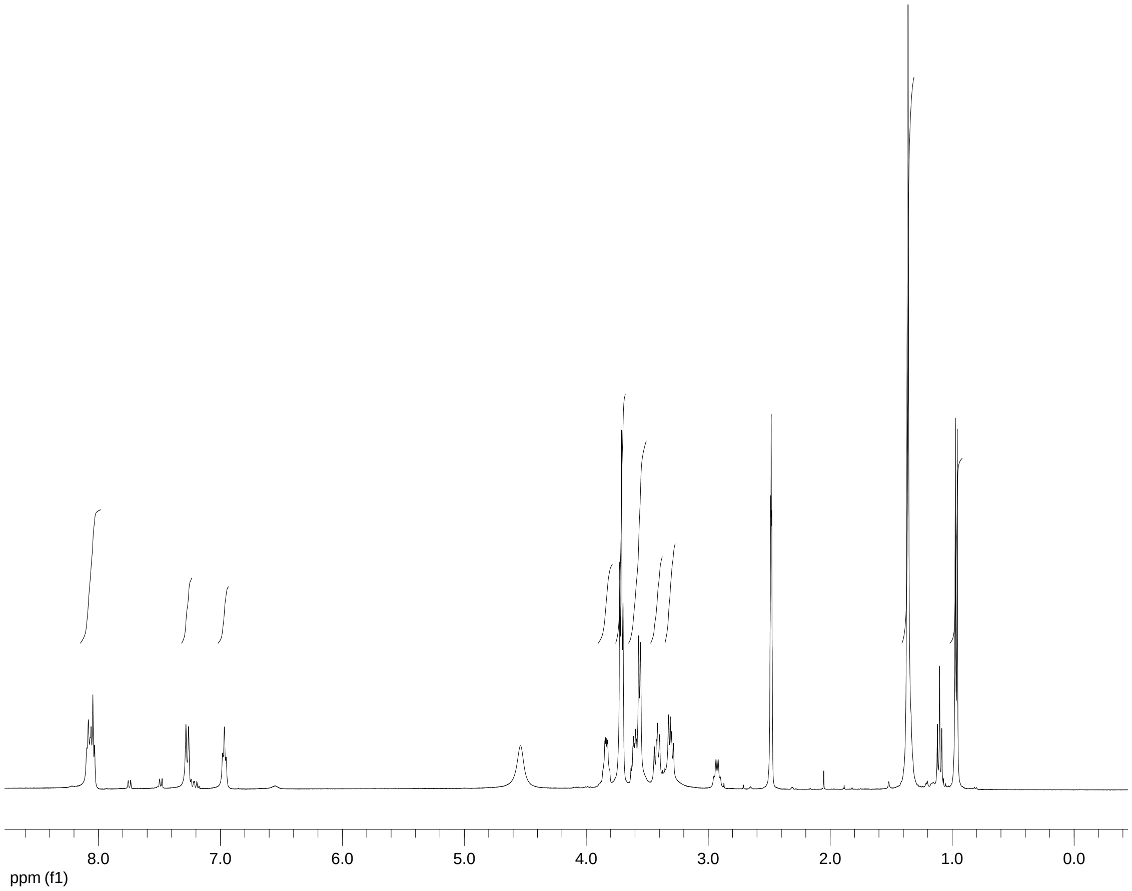

8.0
7.0
6.0
5.0
4.0
3.0
2.0
1.0
0.0
ppm (f1)

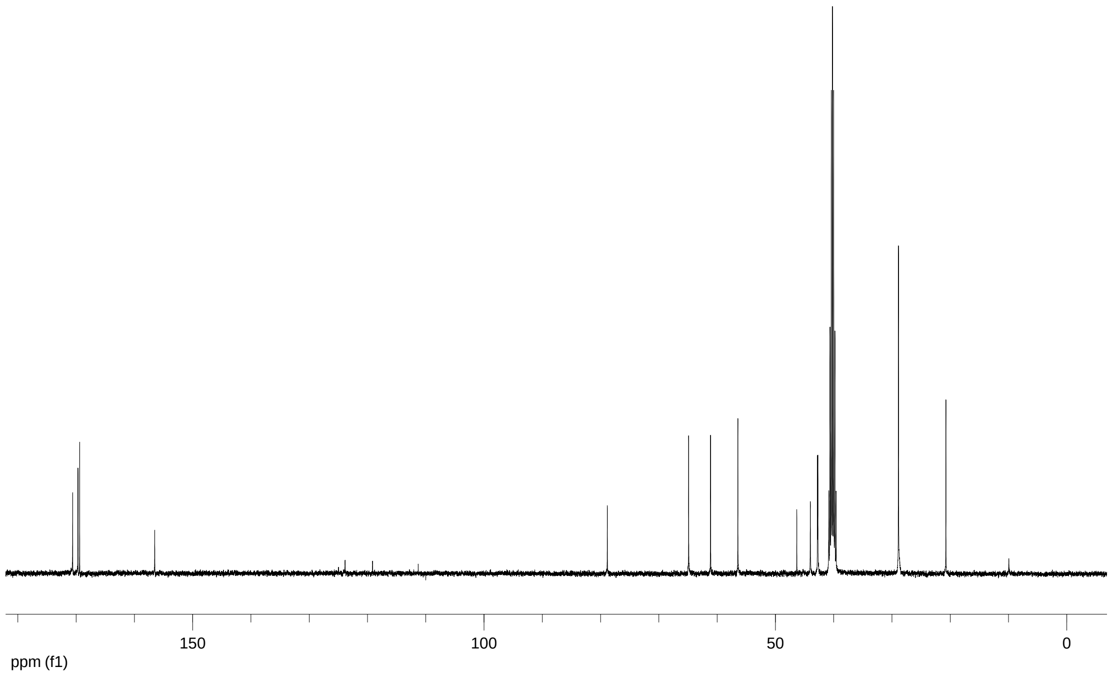

150
100
50
0
ppm (f1)

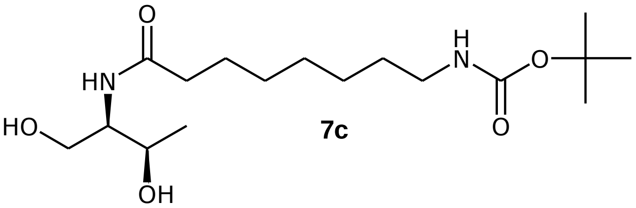

7c

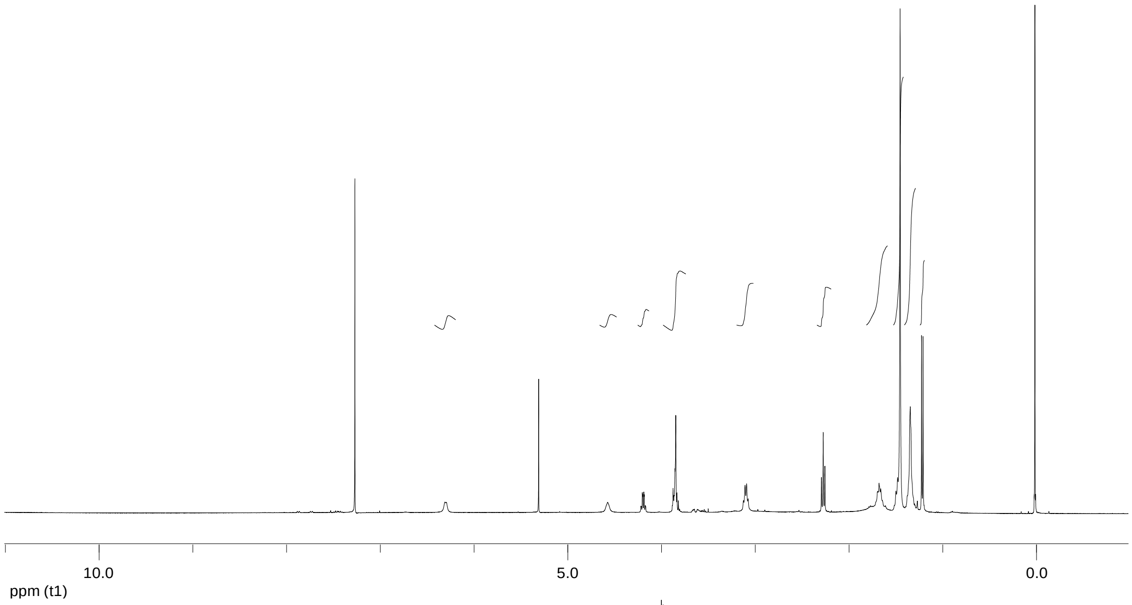

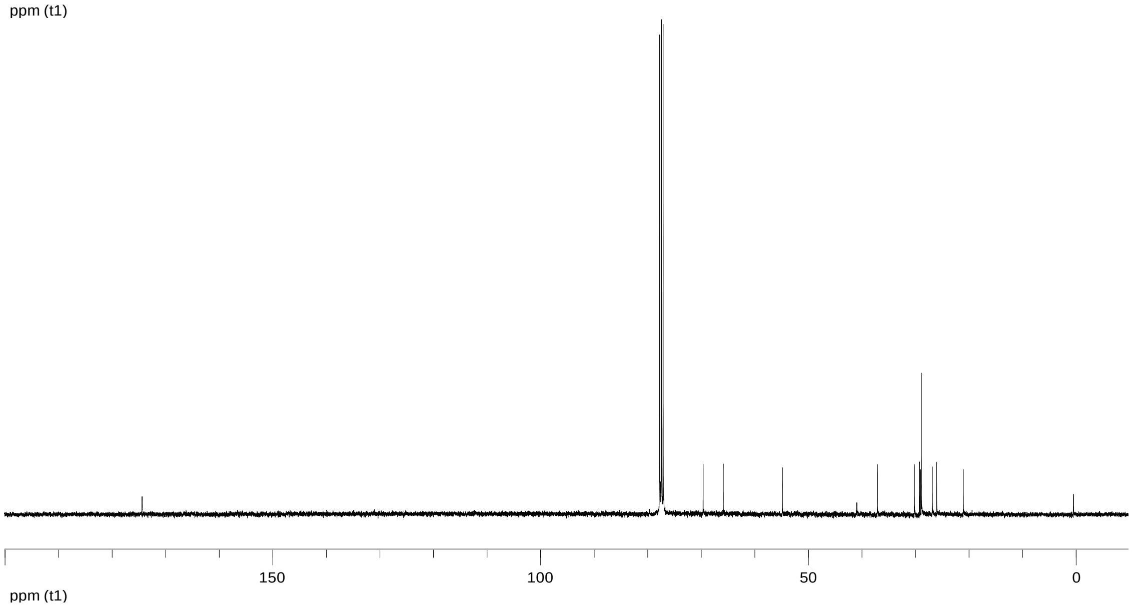

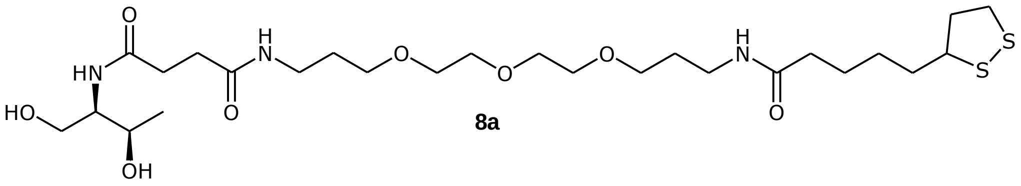
O
HN
HO
OH
O
H
N
O
O
O
H
N
O
S
S

8a

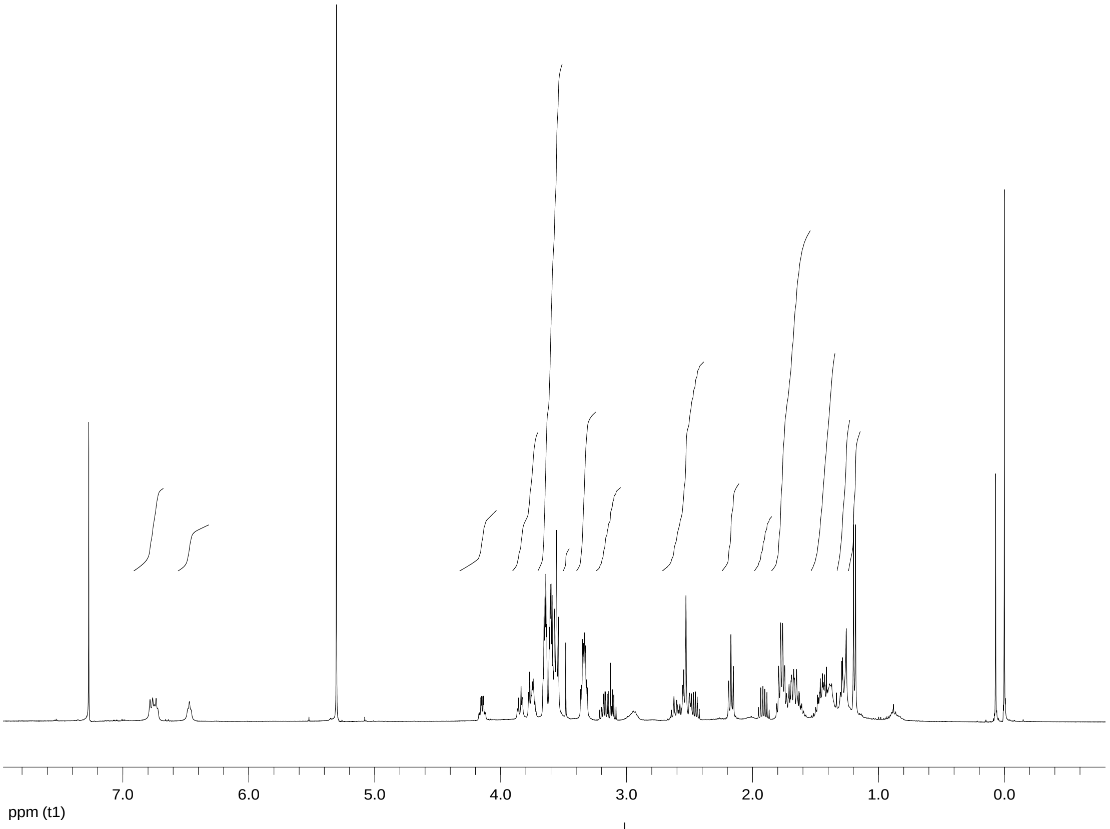
7.0
6.0
5.0
4.0
3.0
2.0
1.0
0.0
ppm (t1)

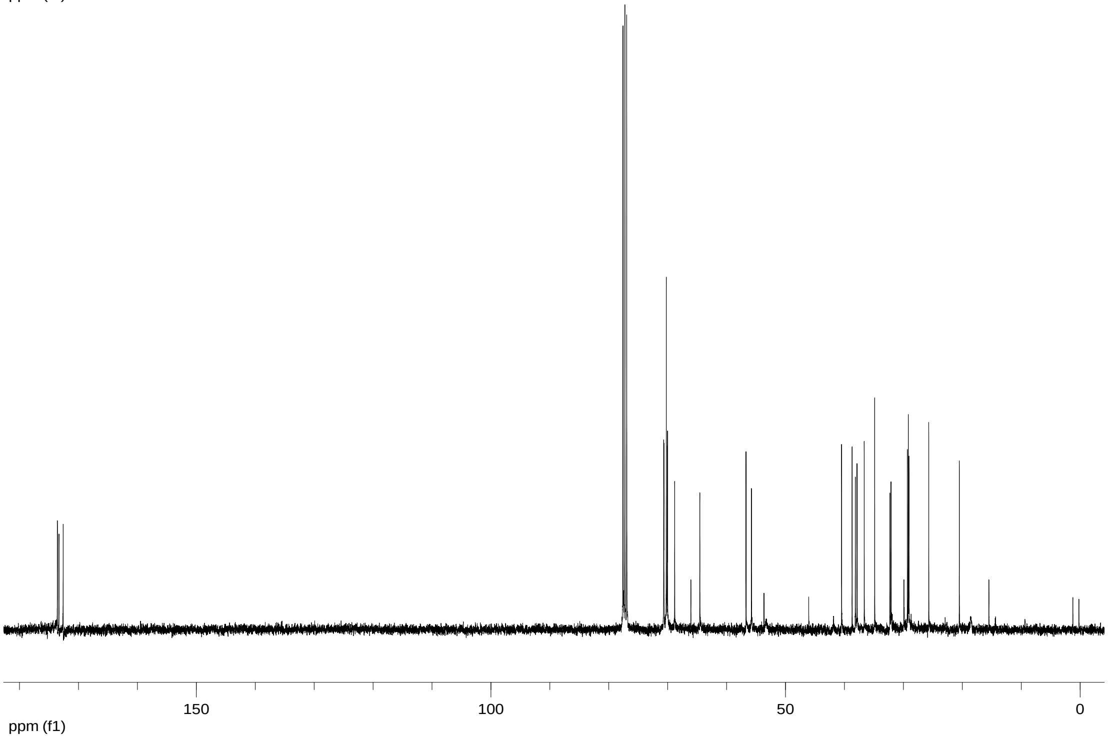
150
100
50
0
ppm (f1)

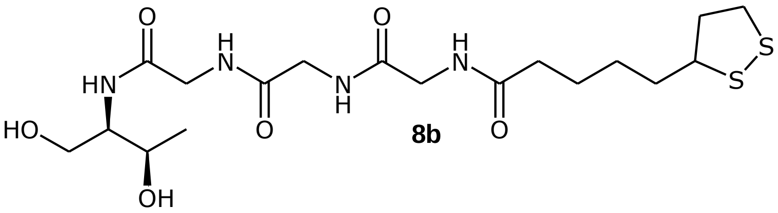

O
HN
H
N
O
N
H
O
H
N
O
S
S
HO
OH

8b

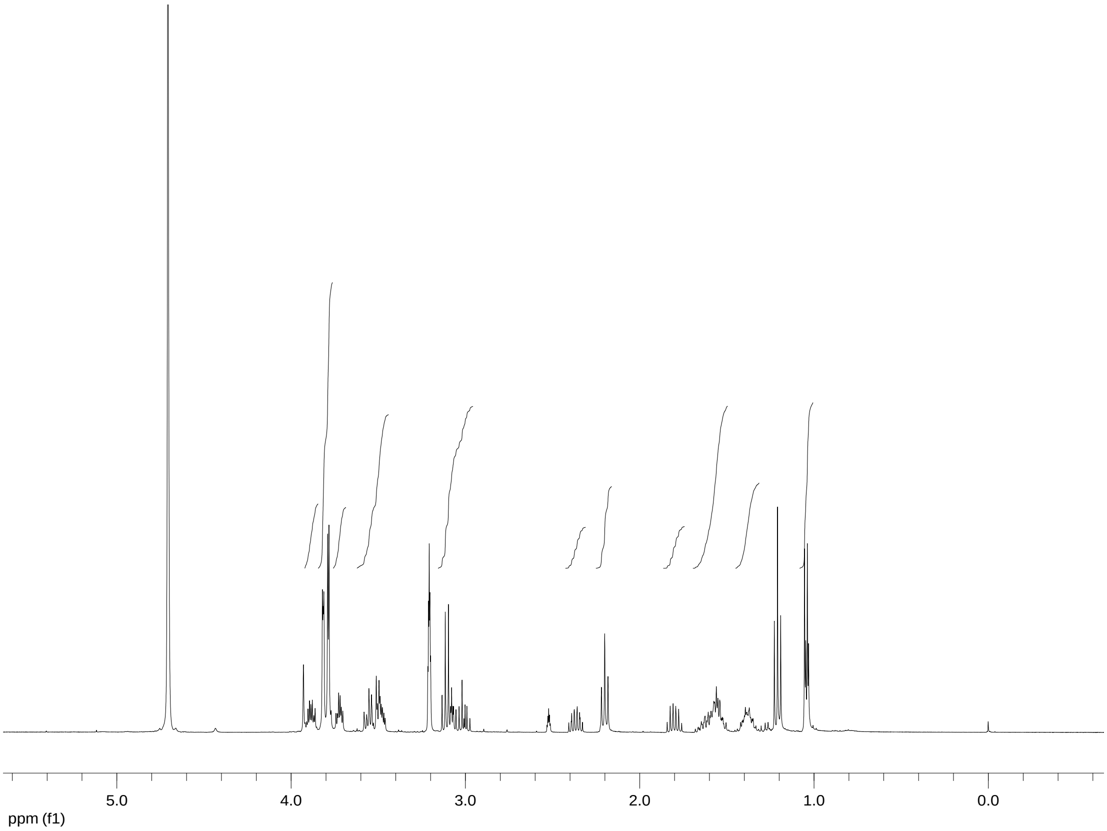

5.0
4.0
3.0
2.0
1.0
0.0
ppm (f1)

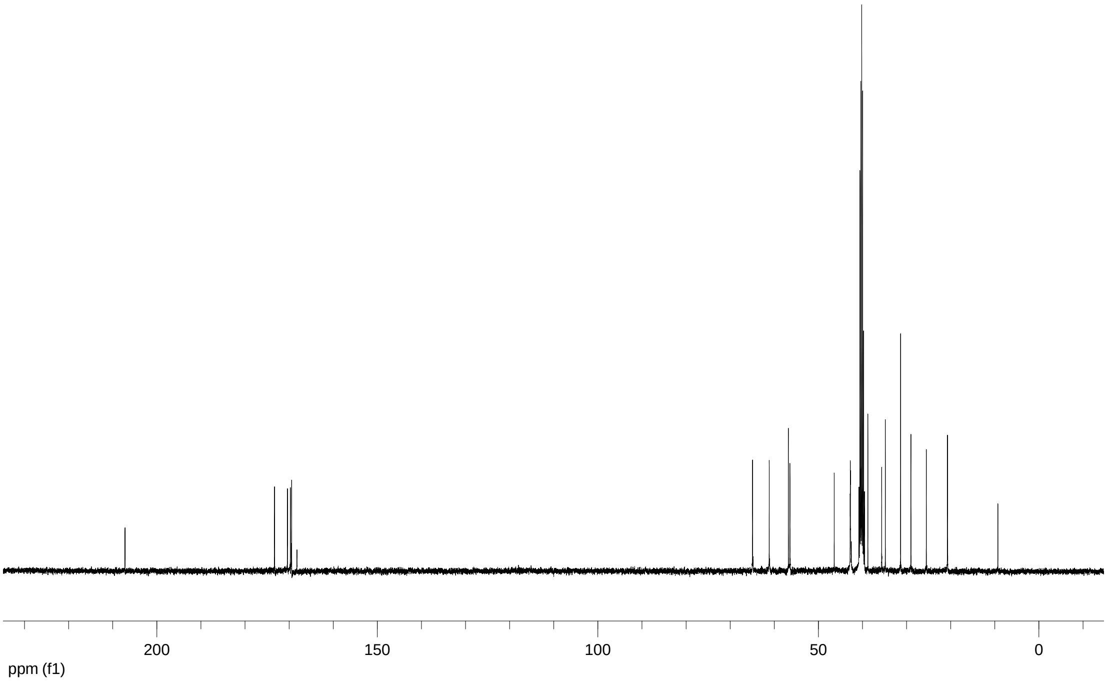

200
150
100
50
0
ppm (f1)

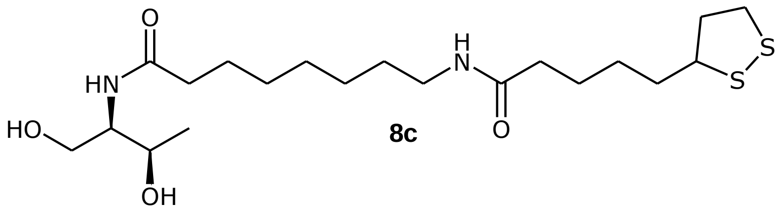

8c

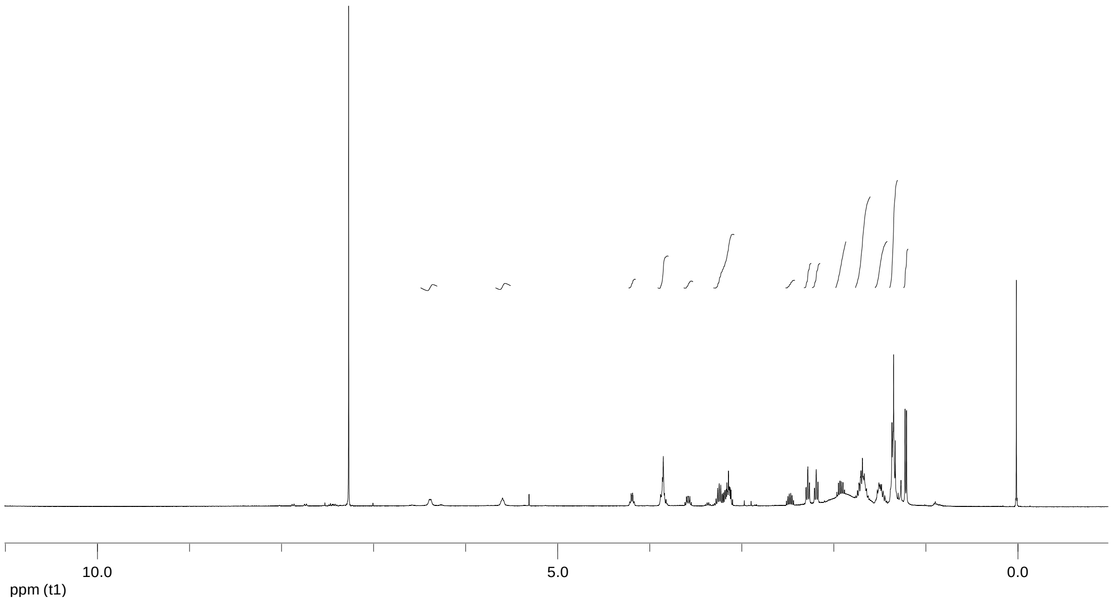

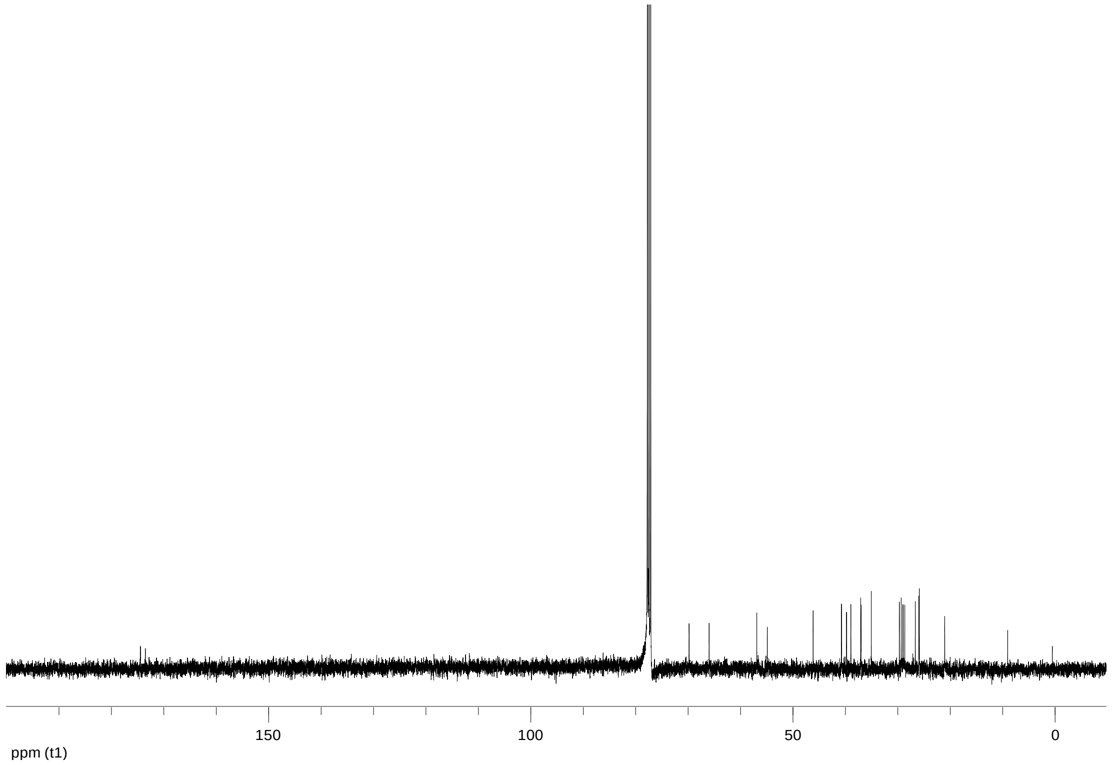

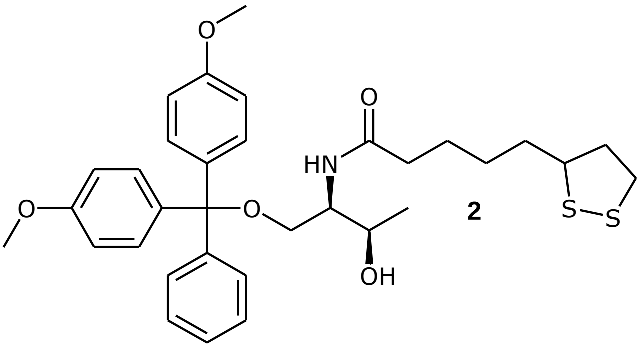

2

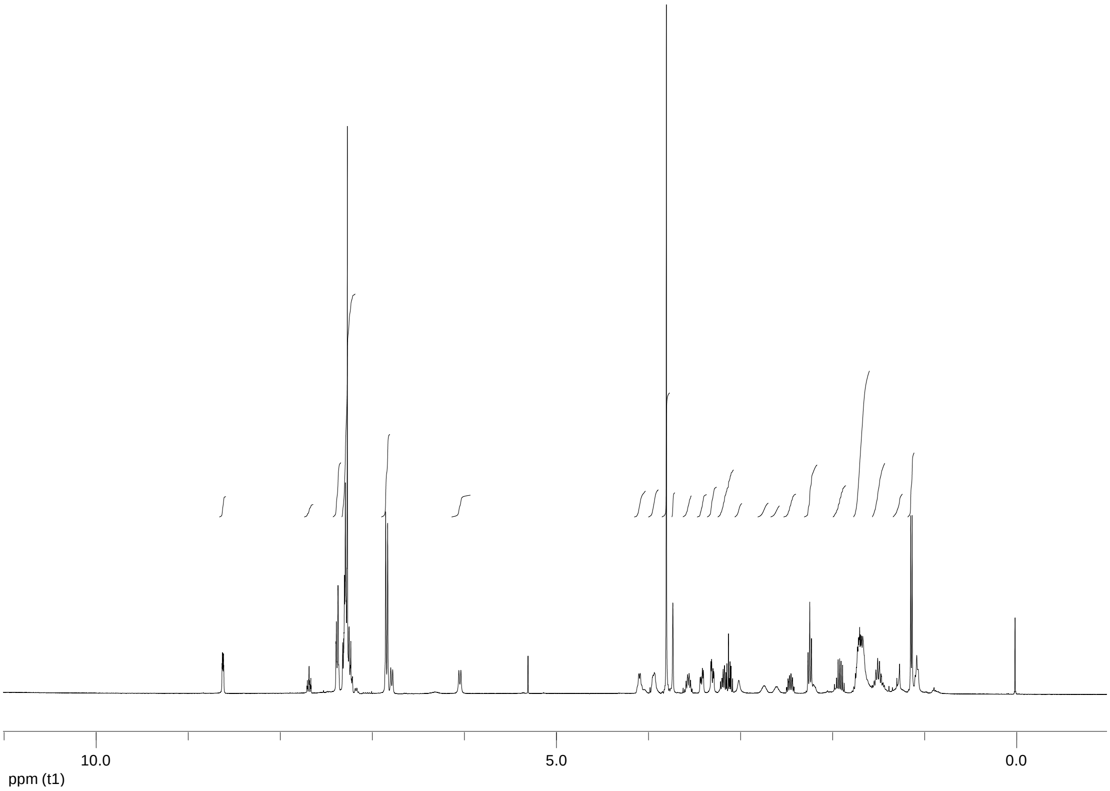

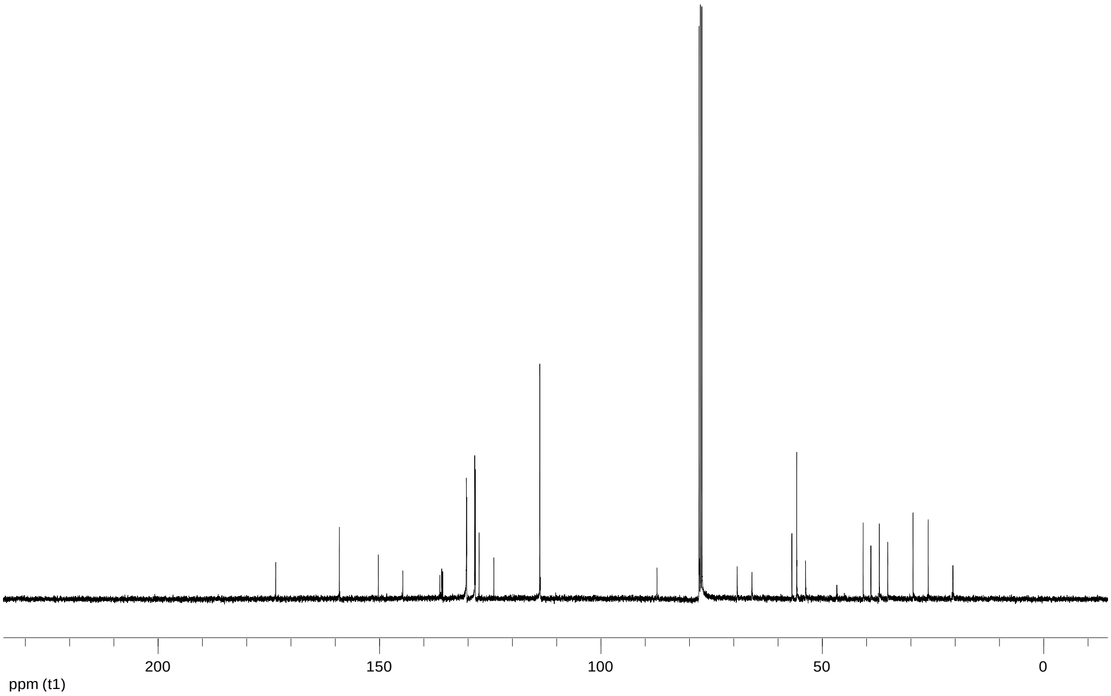

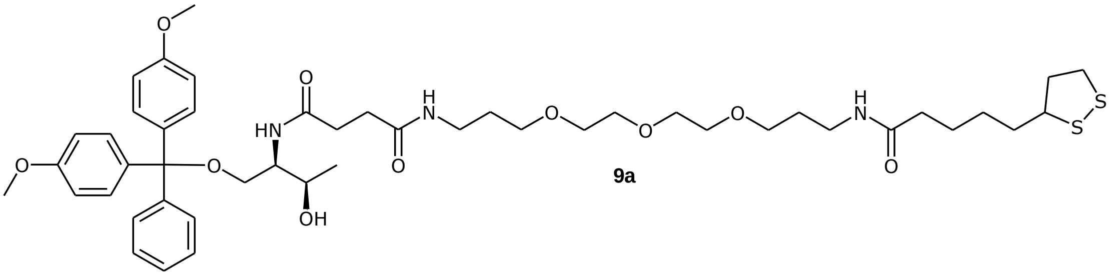
O
O
HN
O
H
N
O
O
O
O
H
N
O
S
S
OH

9a

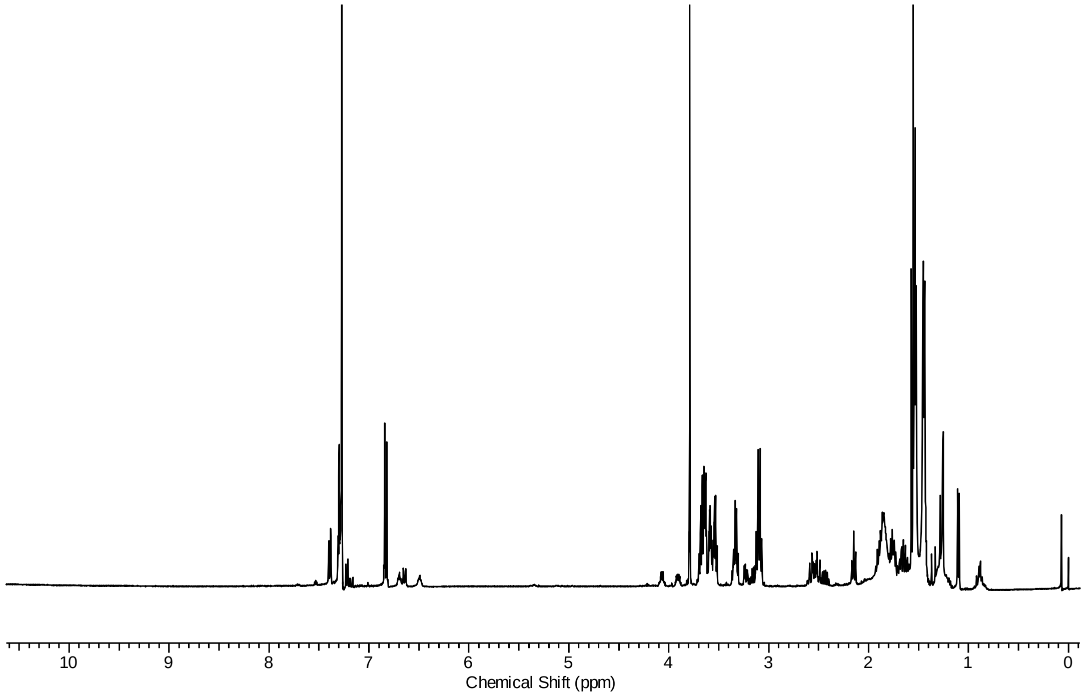
10
9
8
7
6
5
4
3
2
1
0
Chemical Shift (ppm)

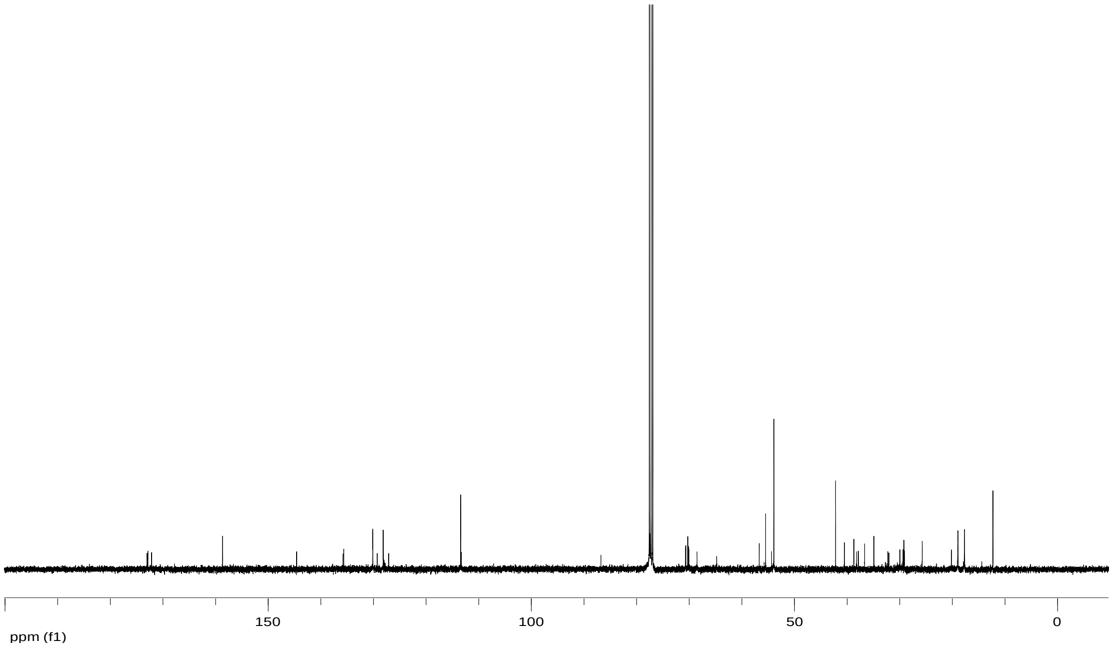
150
100
50
0
ppm (f1)

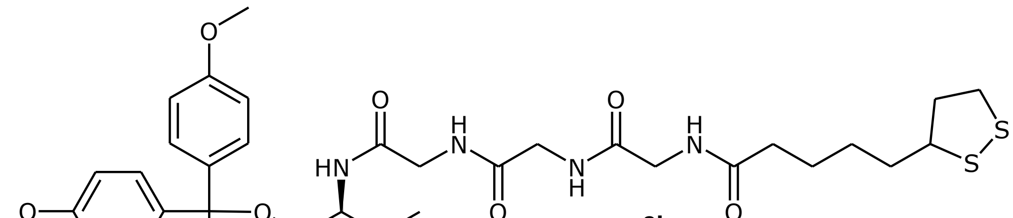
O
O
O
HN
OH
H
N
N
H
H
N
S
S

9b

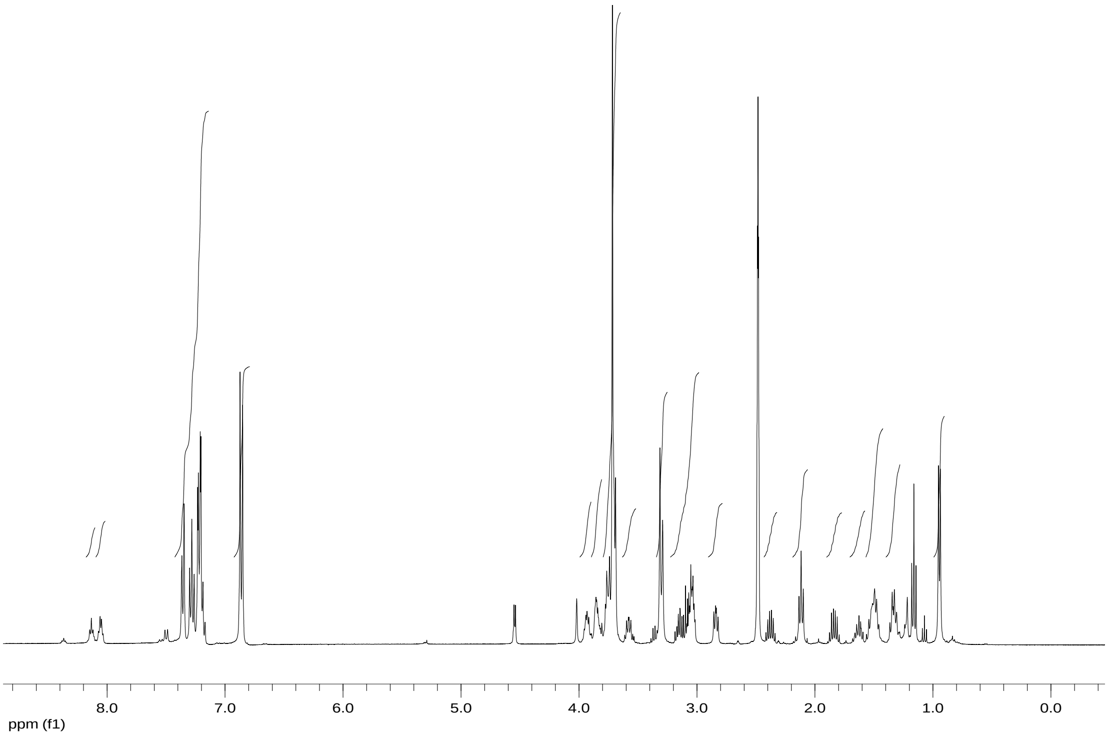
8.0
7.0
6.0
5.0
4.0
3.0
2.0
1.0
0.0
ppm (f1)

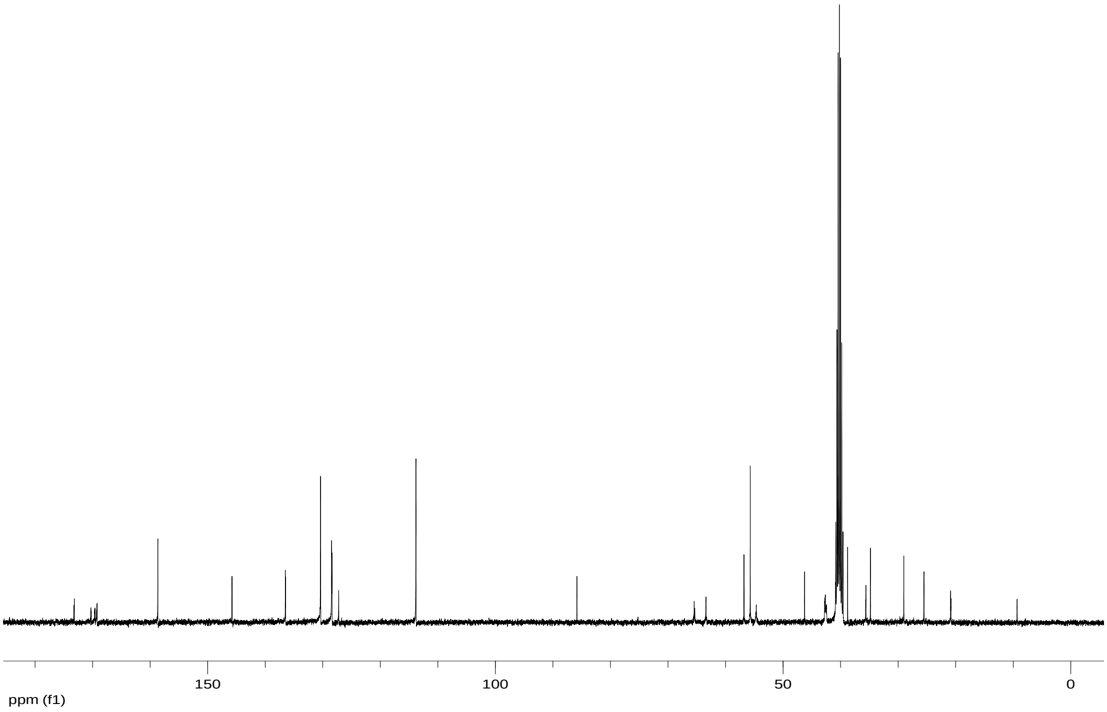
150
100
50
0
ppm (f1)

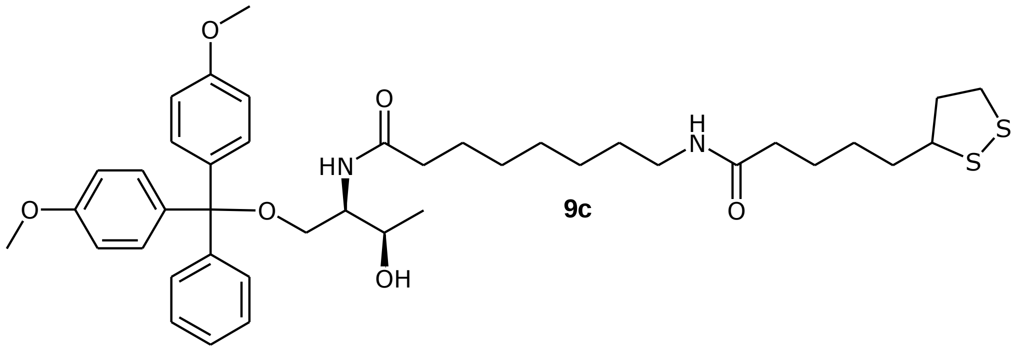
O
O
O
HN
O
OH
9c
H
N
O
S
S

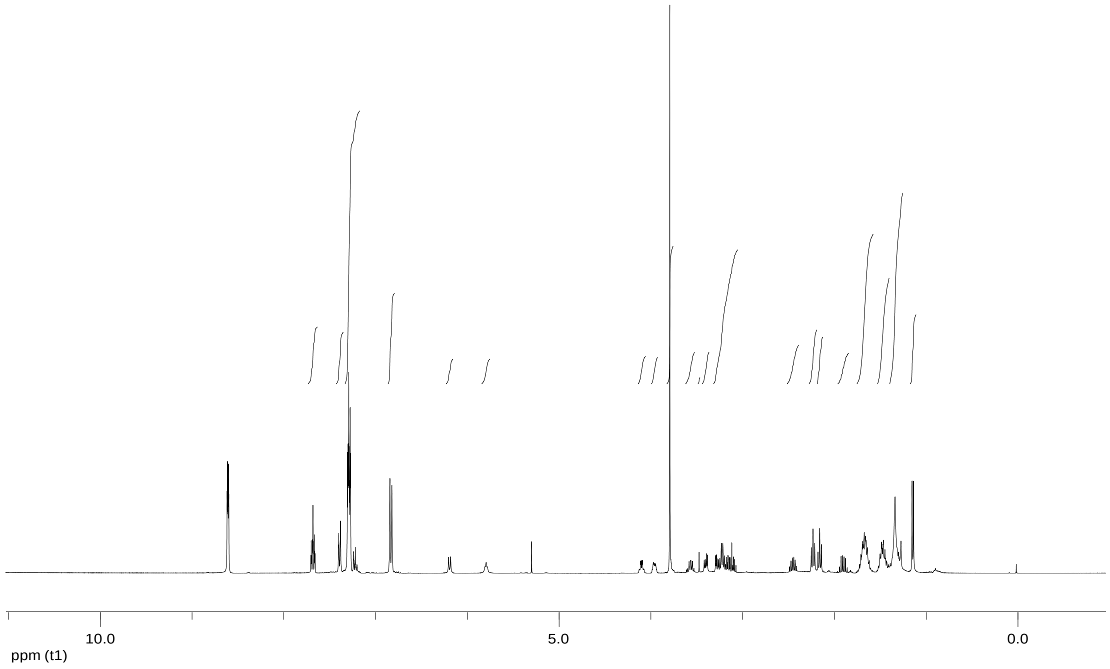
10.0
5.0
0.0
ppm (t1)

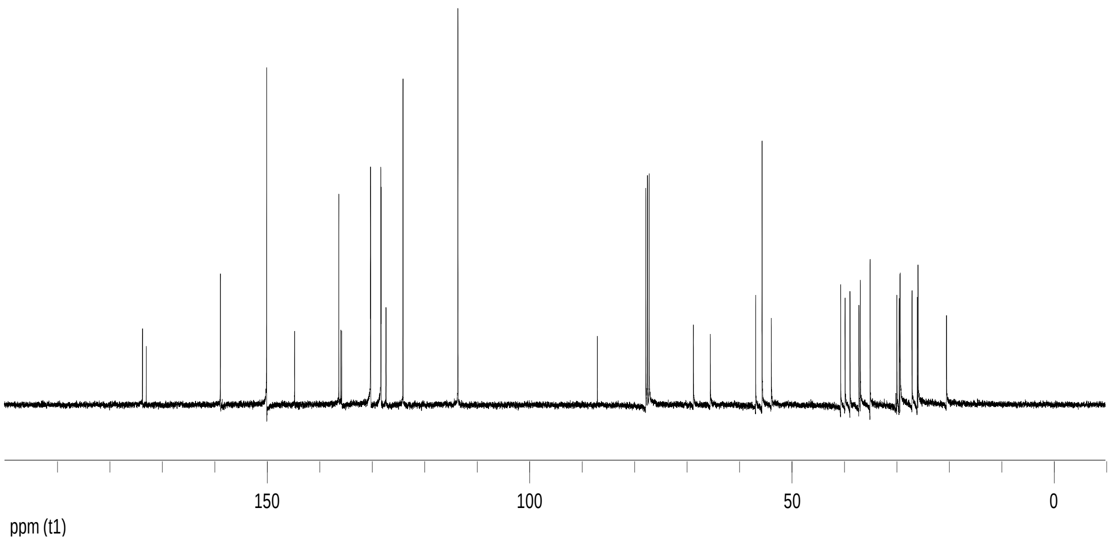
150
100
50
0
ppm (t1)

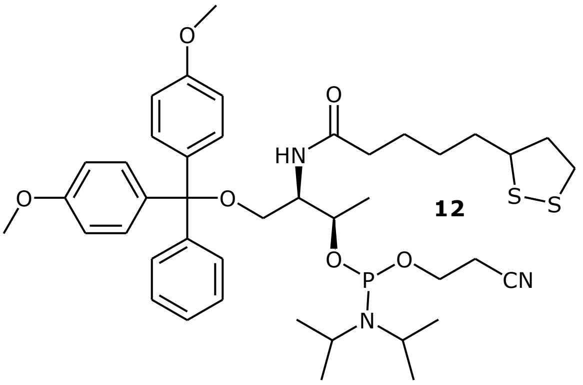

12

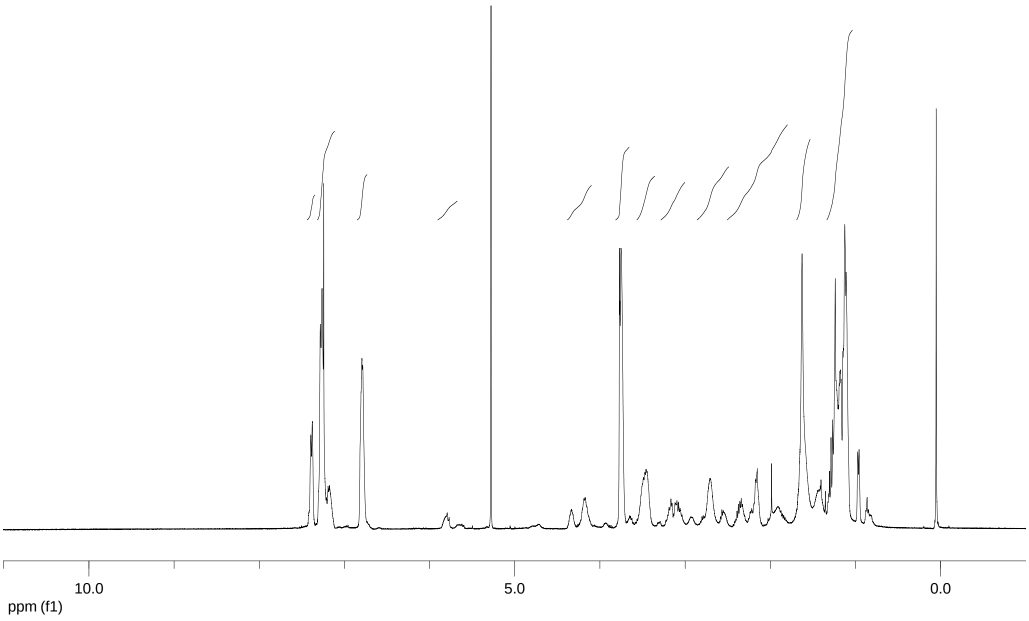

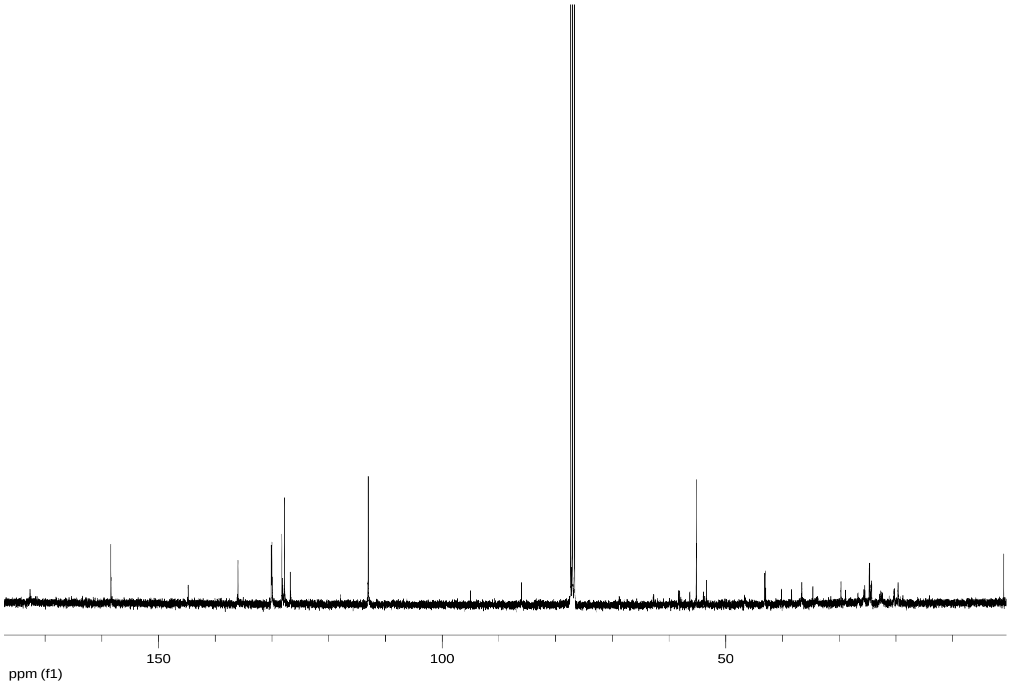

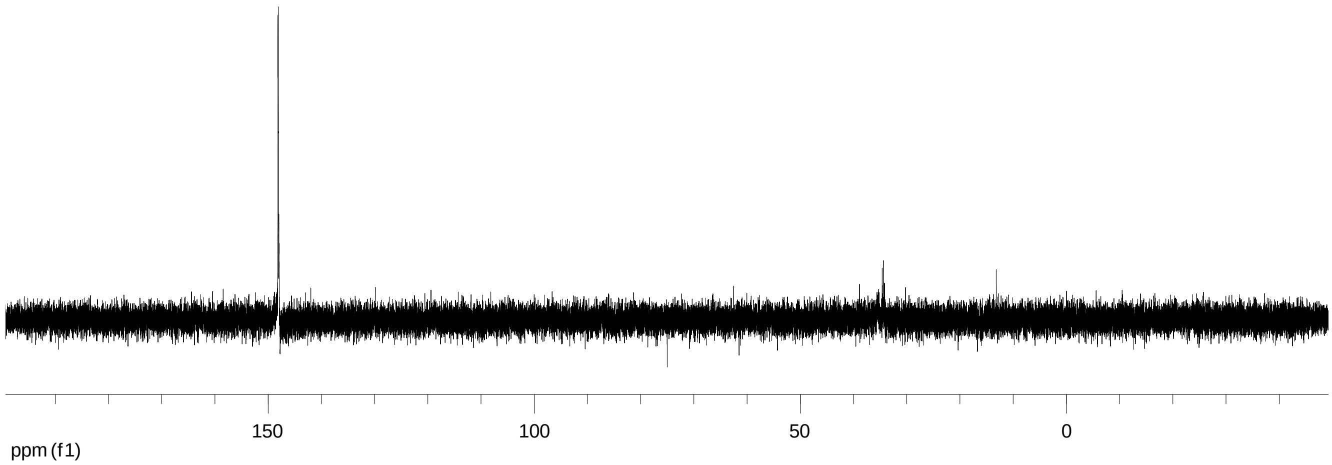
150
100
50
0
ppm (f1)
